# Two non-identical twins in one unit cell: characterization of 34π aromatic core-modified octaphyrins, their structural isomers and anion bound complexes†
†Electronic supplementary information (ESI) available: Experimental procedures and characterization of all new compounds and crystallographic data for compound **10** (1857151), **10**·2HClO_4_ (1857152), **10**·H_2_SO_4_ (1857153), **15** (1887399) and **15**·2HClO_4_ (1903728). CCDC 1857151–1857153, 1887399 and 1903728. For ESI and crystallographic data in CIF or other electronic format see DOI: 10.1039/c9sc01633c


**DOI:** 10.1039/c9sc01633c

**Published:** 2019-05-15

**Authors:** Arindam Ghosh, Syamasrit Dash, A. Srinivasan, Cherumuttathu H. Suresh, Tavarekere K. Chandrashekar

**Affiliations:** a School of Chemical Sciences , National Institute of Science Education and Research (NISER) , HBNI , Bhubaneswar-752050 , Odisha , India . Email: tkc@niser.ac.in; b Inorganic and Theoretical Chemistry Section , Chemical Sciences and Technology Division , CSIR-National Institute of Interdisciplinary Science and Technology , Trivandrum-695019 , Kerala , India

## Abstract

Four different core-modified planar 34π octaphyrins (**10**, **11**, **13**, and **15**) which exhibit rotational isomerism have been synthesized and characterized both in solution and solid states. Octaphyrins **10**, **11** and **13** show two inseparable isomers **A** and **B** which crystallize in the same unit cell. However, **15** forms two identical isomers of **A**. Structurally, the two isomers in **10**, **11** and **13** (**A** and **B**) are different only in the ring inversion of one of the thiophene or selenophene rings present in the terthiophene subunit of the macrocycle. In isomer **A**, the middle thiophene or selenophene rings are inverted, while in isomer **B**, the terminal thiophene rings are inverted. The ^1^H NMR spectrum of these macrocycles shows peaks assignable to protons of both the isomers in toluene D_8_. The single crystal structure analysis of **10** reveals the presence of both isomers **10A** and **10B** in a single unit cell with the *P*2_1_/*n* space group. Both the isomers exhibit aromatic behaviour in the freebase form. Protonation of pyrrole nitrogens leads to exclusive formation of isomer **B** for **10** and **11**. However, both the isomers are present upon protonation of **13** where the central heterocyclic ring of terthiophene subunits has thiophene and selenophene rings. Octaphyrin **15** crystallizes in the *P*2_1_/*c* space group and exclusively isomer **A** was formed in the reaction. Protonation of pyrrole nitrogens leads to significant increases in aromaticity as revealed by ^1^H NMR chemical shift data. The NICS values calculated for the individual heterocyclic rings before and after protonation support such a conclusion. The AICD plots exhibit clockwise orientation of current density vectors suggesting the presence of diatropic ring current in the octaphyrins. Energy calculations at the M06L/CC-pVTZ//M06L/6-31G** level qualitatively account for exclusive stabilization of a particular isomer relative to the other upon protonation. To the best of our knowledge **10** represents the first example in expanded porphyrin chemistry where two different structural isomers crystallize in a single unit cell.

## Abstract 2

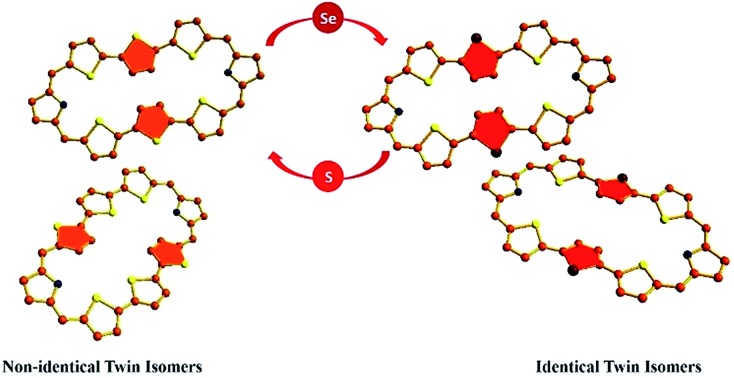
Two inseparable isomers **A** and **B** (*non-identical twins*) crystallize in a single unit cell. However, replacement of middle thiophene ring by selenophene ring results in crystallization of two molecules of isomer **A** (*identical twins*).

## 1. Introduction

An octaphyrin is an eight-pyrrole containing macrocycle in which pyrrole rings are connected in a cyclic fashion *via meso* carbon bridges and/or with some direct pyrrole–pyrrole links.^1^ Octaphyrins are known to be conformationally flexible and are shown to adopt twisted figure-eight,^2^ dumbbell,^3^ aromatic planar,^4^ and antiaromatic planar^5^ conformations. The attainment of a figure-eight conformation results in loss of aromaticity.^6^ Hence, to synthesize planar aromatic octaphyrins, various synthetic approaches have been adopted. We used an approach to substitute sterically bulky mesityl substituents at the *meso* positions to avoid twisting of the macrocycle to synthesize **1** (ref. 4) which is a 34π planar aromatic core-modified octaphyrin. Later Osuka and co-workers followed a bridging approach to tie a bridge across the *meso* carbons to avoid twisting and were successful in synthesizing **2a** which turned out to be non-aromatic.^7^ Later, we used a similar bridging approach to synthesize 34π aromatic bridged octaphyrin **2b**.^8^
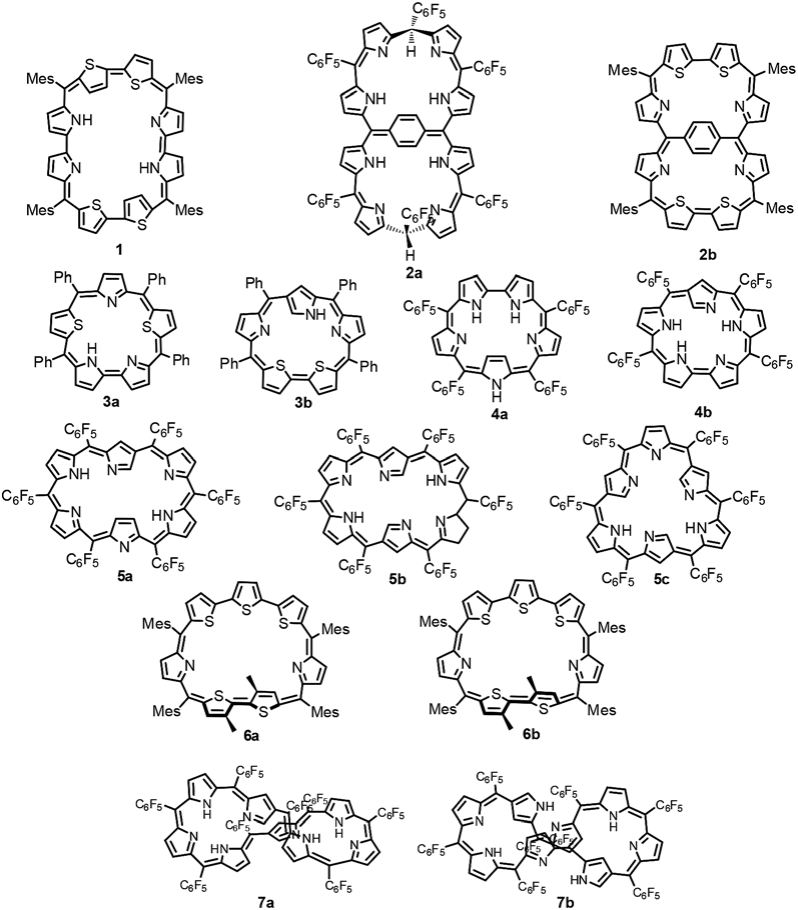


Structural isomers of porphyrins such as porphycenes,^9^ corrphycenes,^10^ hemiporphycenes^11^ and N-confused porphyrins^12^ and their diverse chemistry have been well documented in the literature.^13^–^15^ However, there are only limited reports on the structural isomers of expanded porphyrins in general and octaphyrins in particular. Latos-Grażyński and co-workers reported the first synthesis of core-modified sapphyrin **3a**.^16^ Later, Chandrashekar and coworkers reported synthesis of **3a** and along with Furuta demonstrated the synthesis of its N-confused isomer **3b**.^17^ In 2008, Furuta reported all aza N-confused sapphyrin **4b** (ref. 19), which is a structural isomer of **4a** (ref. 18) in which one of the pyrrole rings has a β-connectivity. The structural isomers of hexaphyrins, singly, doubly and triply N-confused hexaphyrins **5a**,^20^**5b** (ref. 21) and **5c** (ref. 22) respectively, were reported by Furuta and Xie in 2009. We have characterized two conformers of heptaphyrin **6a** and **6b**.^23^ Furthermore, the existence of two tautomers of **1** where the pyrrole NH proton is exchanging between imine and amine pyrrolic nitrogens was structurally characterized by our group.^4^ Recently, Furuta and Xie reported the synthesis of a neo-confused octaphyrin which is an isomer of all aza figure-eight octaphyrin.^24^ More recently, Ishida and Furuta reported the synthesis of a doubly N-confused 36π octaphyrin which exhibits isomerization between figure-eight **7a** and dumbbell **7b** structures.^3^ However, upon bis-metallation only the figure-eight structure is stabilized relative to the dumbbell structure. In all the above examples the structural isomers have been independently synthesized by various synthetic routes and their chemistry has been reported.

The brief literature survey described above clearly reveals that the characterization of structural isomers of expanded porphyrins in general and octaphyrins in particular is in its infancy and more studies are needed to understand the structural diversity of expanded porphyrins to exploit their rich and diverse chemistry.^25^ Recently, we and others have shown that aromatic octaphyrins are good NLO materials^26^ and they exhibit bicyclic Baird type aromaticity.^27^ Thus, in this article we wish to report the characterization of new structural isomers of 34π modified octaphyrins which exhibit rotational isomerization through C–C bond rotation. Three 34π aromatic modified octaphyrins have been synthesized and characterized by varying the heteroatom present in the expanded porphyrin core.

The sulphur analogue **10** exhibits two rotational isomers **10A** and **10B** in a 1 : 1 ratio. However, replacing one of the central thiophene rings by a selenophene ring in the terthiophene moiety gave two new isomers **13A** and **13B** in a 4 : 1 ratio. Replacement of both central thiophene rings by selenophene rings results in the formation of two identical isomers (**15A** and **15A′**) in which both the central selenophene rings are inverted. Characterization of all these isomers has been done using UV-VIS, ^1^H and 2D-NMR and single crystal X-ray structure analyses. It has been shown that isomers **10A** and **10B** crystallized in a single unit cell with the *P*2_1_/*n* space group which is unprecedented to the best of our knowledge.

## 2. Results and discussion

### 2.1. Syntheses

**Scheme 1:**
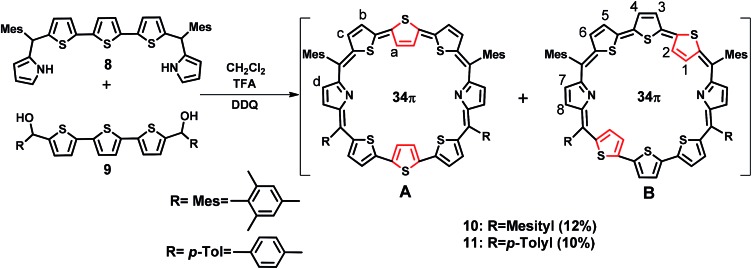
Synthesis of octaphyrins **10** and **11**.

The synthesis of a terthiophene based [34]π octaphyrin is outlined in Scheme 1. We have adopted a [5 + 3] acid-catalyzed MacDonald type condensation reaction. The required precursors 5,5′′-bis(mesityl(1*H*-pyrrol-2-yl)methyl)-2,2′:5′,2′′-terthiophene **8** and [2,2′:5′,2′′-terthiophene]-5,5′′-diylbis(arylmethanol) **9** were synthesized using our earlier reported procedures.^28^ Thus the condensation of **8** and **9** in the presence of 1 equiv. of trifluoroacetic acid (TFA) in dry CH_2_Cl_2_ followed by oxidation with 2,3-dichloro-5,6-dicyano-1,4-benzoquinone (DDQ) gave octaphyrins **10** and **11** in 10–12% yield.

The macrocycles **10** and **11** are stable and their composition was confirmed through ESI mass spectrometry (*m*/*z*, 1145.3190 for **10** and *m*/*z*, 1089.2703 for **11**) (Fig. S1 and S2, ESI†).

**Scheme 2:**
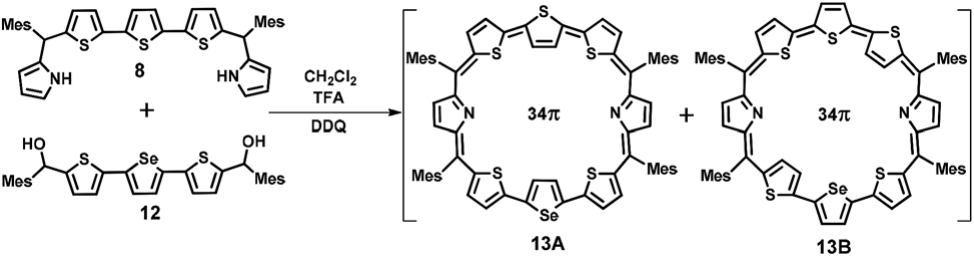
Synthesis of octaphyrin **13**.

For the synthesis of **13** in which one of the central thiophene rings of the terthiophene moiety is replaced by a selenophene ring, the required precursor **12** was synthesized by using a reported procedure.^29^ Thus, the condensation of **8** and **12** under acid-catalyzed conditions in CH_2_Cl_2_ followed by DDQ oxidation (Scheme 2) gave **13**, a dark blue band on a silica gel column (100–200 mesh) eluted with CH_2_Cl_2_/*n*-hexane (58 : 42 v/v) in 9% yield. Here also isomers **13A** and **13B** were inseparable and **13A** was a major isomer while **13B** is minor. The composition was confirmed through ESI mass spectrometry (*m*/*z*, 1193.2602 for **13**) (Fig. S3, ESI†).

**Scheme 3:**
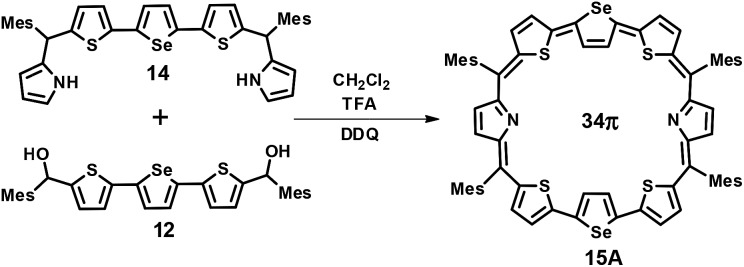
Synthesis of octaphyrin **15**.

The third octaphyrin **15** was synthesized by the [5 + 3] approach (Scheme 3). The required precursor **14** was synthesized using our earlier reported procedure.^28^ Acid-catalyzed condensation of **12** and **14** in CH_2_Cl_2_ followed by DDQ oxidation gave **15**. The compound was eluted with CH_2_Cl_2_/*n*-hexane (52 : 48 v/v) in 10% yield. The reaction afforded isomer **A** exclusively whereas isomer **B** was not formed. The composition was confirmed through ESI mass spectrometry (*m*/*z*, 1241.2308 for **15**) (Fig. S4, ESI†).

### 2.2. Spectral characterization

#### (a). UV-Visible

**Fig. 1:**
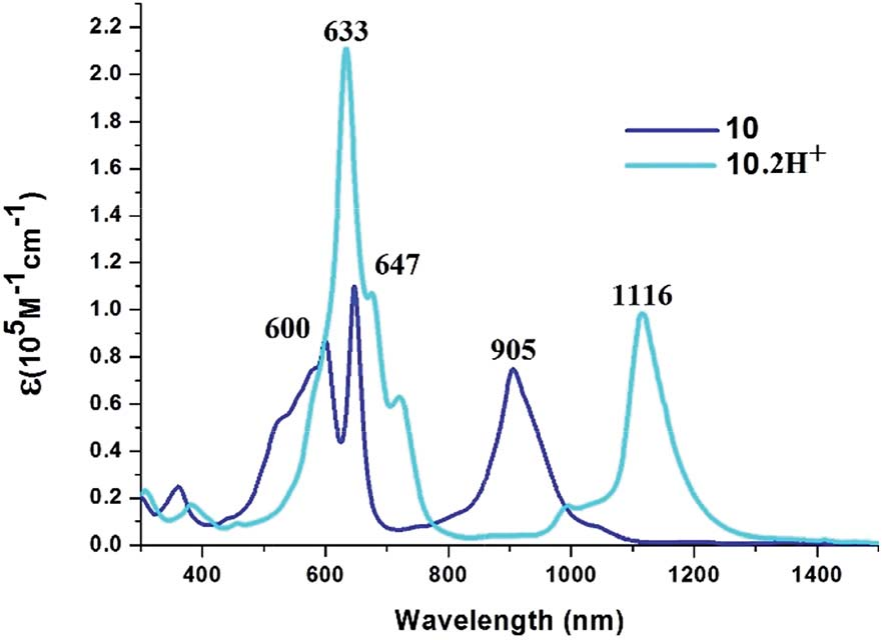
Electronic absorption spectra of **10** and **10**·2H^+^ in CH_2_Cl_2_.

The electronic absorption spectra of **10**, **11**, **13** and **15** were recorded in CH_2_Cl_2_ solution. In all the cases, a split Soret like band between 600 and 650 nm and a Q-type band from 900–920 nm were observed, suggesting the porphyrinic nature of the macrocycles. There are small changes in absorption maxima on varying solvent polarity (Fig. S5 and S9, ESI†), suggesting no conformational change. Upon protonation of pyrrolic nitrogens with dilute solution of TFA in CH_2_Cl_2_, the Soret like band is moderately red shifted and the Q-like band experiences a red shift of >200 nm with a two fold increase in the Soret type band intensity. The representative absorption spectra of **10** shown in Fig. 1 exhibit Soret like bands at 600 nm (*ε* = 8.65 × 10^4^ dm^3^ mol^–1^ cm^–1^) and 647 nm (*ε* = 1.10 × 10^5^ dm^3^ mol^–1^ cm^–1^) and a broad Q-like band at 905 nm (*ε* = 7.45 × 10^4^ dm^3^ mol^–1^ cm^–1^). Protonation leads to red shift of the Soret type band to 633 nm (*ε* = 2.10 × 10^5^ dm^3^ mol^–1^ cm^–1^) with a two fold increase in *ε* values and the Q-like band to 1116 nm (*ε* = 9.80 × 10^4^ dm^3^ mol^–1^ cm^–1^). These changes upon protonation are typical of *meso*-aryl expanded porphyrins.^30^ Careful titration of dilute solution of TFA in CH_2_Cl_2_ leads to quenching of absorption followed by shift of the absorption bands with the appearance of isosbestic points (Fig. S7 and S8, ESI† for **10**), suggesting the binding of TFA anions to the macrocycle. The binding constants evaluated is of the order of 10^3^ M^–1^, suggesting a moderate binding. Similar absorption spectral trends were observed in **11**, **13** and **15** (Fig. S10–S14, ESI†).

#### (b). ^1^H NMR studies

**Fig. 2:**
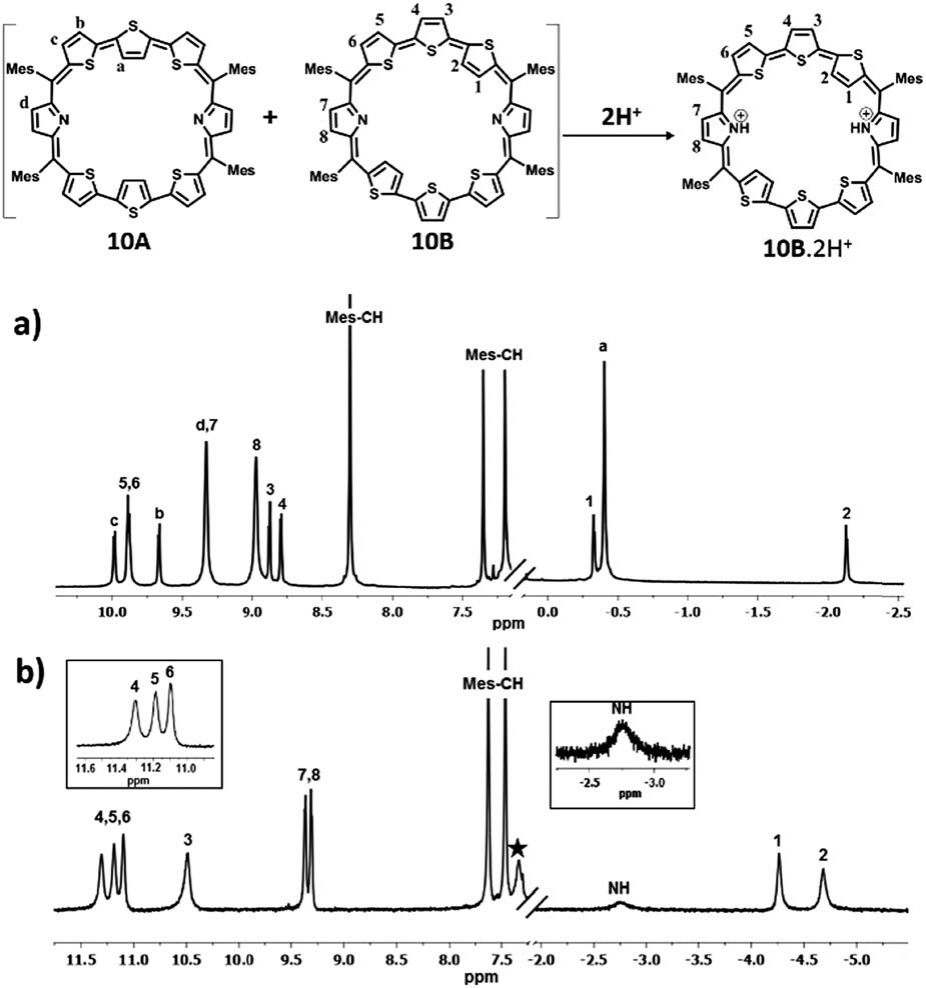
^1^H NMR spectrum: (a) **10** (213 K) and (b) **10**·2H^+^(298 K) in toluene D_8_ (* residual solvent peaks).

The solution structures of the various octaphyrins were proved by ^1^H and 2D NMR studies. The ^1^H NMR spectrum of **10** in toluene D_8_ (Fig. 2) shows more signals than expected suggesting the presence more than one isomer in solution. The assignment marked was based on ^1^H–^1^H COSY correlation experiments and the assignment of NH protons was confirmed by D_2_O exchange experiments. The central inverted thiophene ring β-CH protons of **10A** appear at 0.37 ppm (a) while the β-CH protons of terminally inverted thiophene rings of **10B** appear at 0.37 (1) and –1.26 (2) ppm at 298 K (Fig. S15 and S16, ESI†). These chemical shifts suggest that the β-CH protons of inverted thiophene rings are experiencing the diatropic ring current of the macrocycle. The normal thiophene β-CH signals of **10A** (b and c) and **10B** (3, 4, 5, and 6) and pyrrolic β-CH signals (d, 7, and 8) appear in the region from 8.49 ppm to 9.85 ppm. The *meso* mesityl CH protons and CH_3_ protons appear in the expected region. Variation of temperature (343 K to 193 K) did not show any significant changes in the chemical shift of various protons (Fig. S17–S21, ESI†). Even intensities did not change much upon temperature variation. These observations suggest that there is no interconversion between the isomers as well as no change in conformation in the temperature range studied. Attempts to study heteronuclear correlations and dipolar interactions using NOESY and ROSEY experiments failed because of the precipitation of the sample upon long standing in solution. ^1^H NMR of **11** also shows a similar behaviour and because of two different *meso* substituents, the symmetry is lowered and this is reflected in the increase in the number of signals. Overall the two inseparable isomers were observed in a 1 : 1 ratio (Fig. S27–S30, ESI†).

Protonation of pyrrolic nitrogens with dilute solution of TFA leads to stabilization of isomer **B** and the ^1^H NMR spectrum of **10B**·2H^+^ shown in Fig. 2b confirms this observation (Fig. S22–S26, ESI†). Specifically, the pyrrolic NH protons which are experiencing the diatropic ring current appear as a broad signal at –2.76 ppm and a D_2_O experiment confirms the assignment. The terminal inverted thiophene ring β-CH protons (1 and 2) are further shielded relative to the freebase form and appear at –4.29 ppm and –4.70 ppm. The β-CH protons of normal thiophene rings (3, 4, 5, and 6) are further deshielded and appear between 10.47 ppm and 11.28 ppm. The β-CH protons of the pyrrole ring (7 and 8) appear as a doublet at 9.29 ppm and 9.34 ppm. The CH protons of mesityl rings appear between 7.45 and 7.61 ppm and methyl signals between 2.01 and 2.75 ppm. Thus, comparison of proton NMR of freebase and protonated forms of **10** clearly suggests stabilization of **10B** upon protonation (Scheme S4, ESI†). **11** shows similar behaviour upon protonation and because of lowering of symmetry separate signals are observed for the two inverted thiophene protons (Fig. S31–S36, ESI†).

**Fig. 3:**
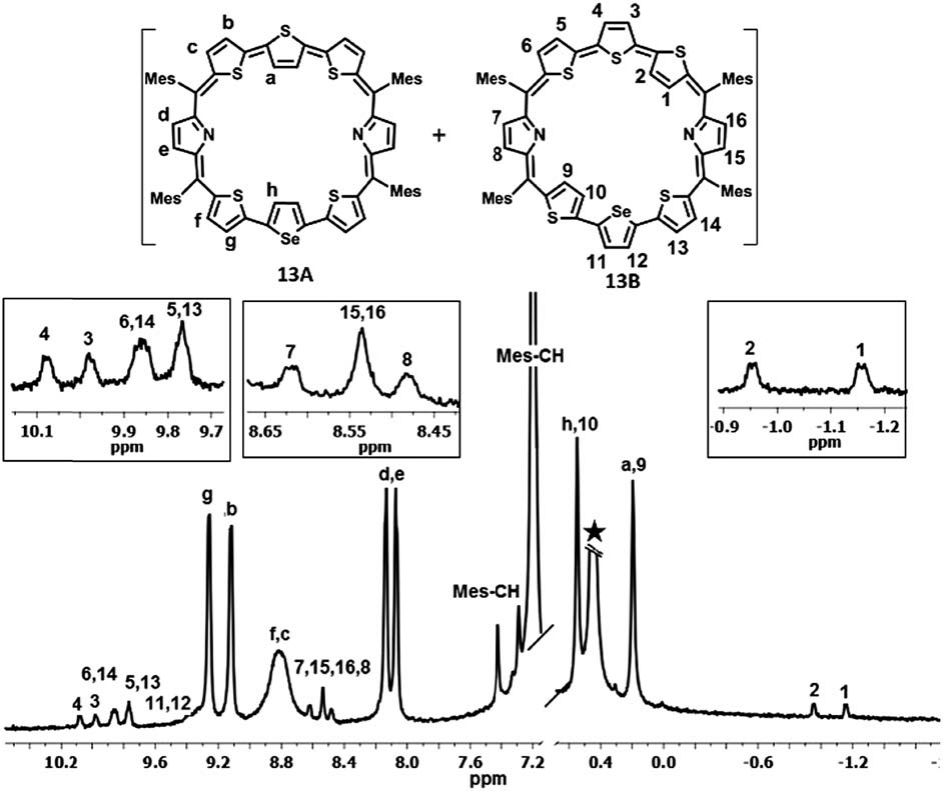
^1^H NMR spectrum of **13** (298 K) in toluene D_8_.

**Fig. 4:**
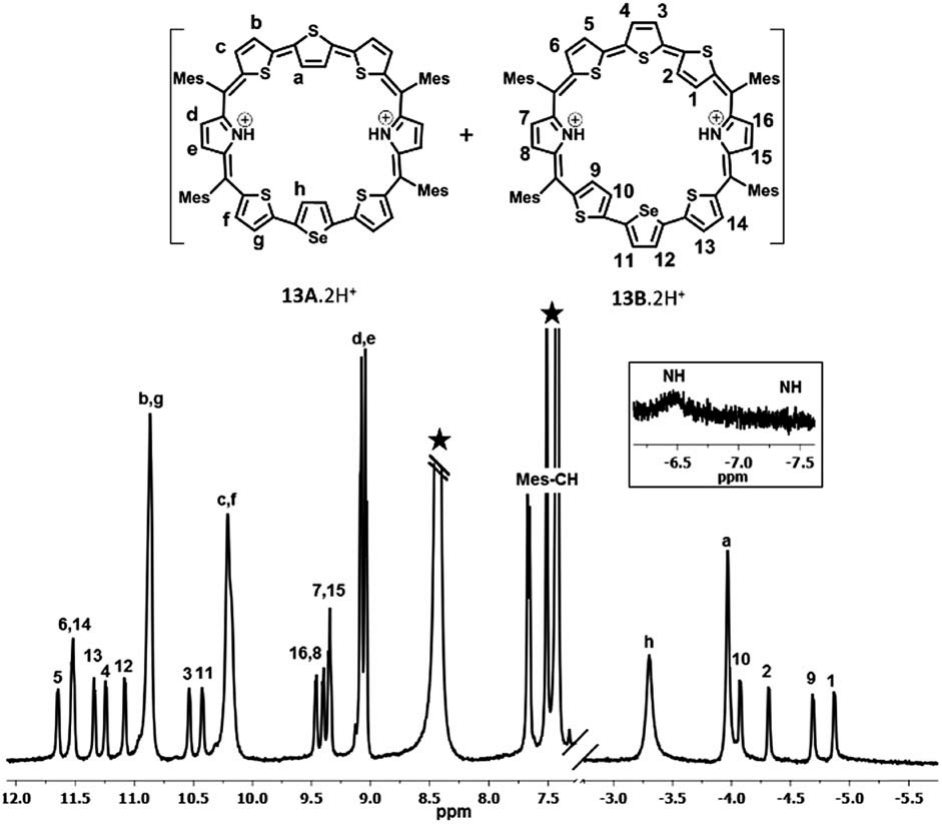
^1^H NMR spectrum of **13**·2H^+^ (298 K) in toluene D_8_.

The proton NMR spectra of freebase and protonated forms of **13** are shown in Fig. 3 and 4. The presence of two different heteroatoms (S and Se) lowers the symmetry of the macrocycle, where one would expect individual signals for the ring protons (Fig. S37–S40, ESI†). For isomer **13A**, the β-CH protons of inverted thiophene (a) and selenophene rings (h) appear at 0.14 ppm and 0.49 ppm respectively, while for **13B**, the terminally inverted thiophene ring protons (1, 2, 9 and 10) appear at 0.49 (10), 0.14 (9), –1.01 (2) and –1.22 (1), suggesting that these protons are experiencing the diatropic ring current of the macrocycle. The β-CH protons of normal thiophene rings of **13A** (c, b, f, and g) appear in the region of 8.73–9.22 ppm, while those of isomer **13B** (3, 4, 5, 6, 11, 12, 13 and 14) appear between 9.4 ppm and 10.03 ppm. The pyrrole β-CH protons of **13A** (d and e) appear in the region of 8.02–8.08 ppm, while those of **13B** (7, 8, 15 and 16) are slightly deshielded and appear in the region from 8.42 ppm to 8.56 ppm. Based on the spectral analysis, both the isomers (**13A** and **13B**) were found in a 4 : 1 ratio. The protonation of pyrrole protons leads to significant shielding and deshielding effects expected for an aromatic macrocycle (Fig. S41 and S42, ESI†). However, both the isomers **13A** and **13B** retain their identity unlike in **10**. The inverted thiophene (a) and selenophene (h) β-CH protons experience significant shielding upon protonation and appear at –3.35 ppm (h) and –4.02 ppm (a). The terminally inverted β-CH protons of thiophene rings (1, 2, 9 and 10) of **13B**·2H^+^ appear between –4.13 ppm and –4.92 ppm. The normal thiophene protons of **13B**·2H^+^ also experience the deshielding effect and appear between 10.47 and 11.6 ppm as eight doublets. The pyrrole β-CH protons of **13A**·2H^+^ (d and e) appear at 8.99–9.02 ppm as two doublets while those of **13B**·2H^+^ (7, 8, 15 and 16) appear in the region of 9.28–9.4 ppm. The pyrrole NH protons of **13**·2H^+^ appear as a broad signal at –6.5 ppm (**13A**·2H^+^) and –7.5 ppm (**13B**·2H^+^). The shielding and deshielding of different protons suggest the aromatic nature of the macrocycles.

**Fig. 5:**
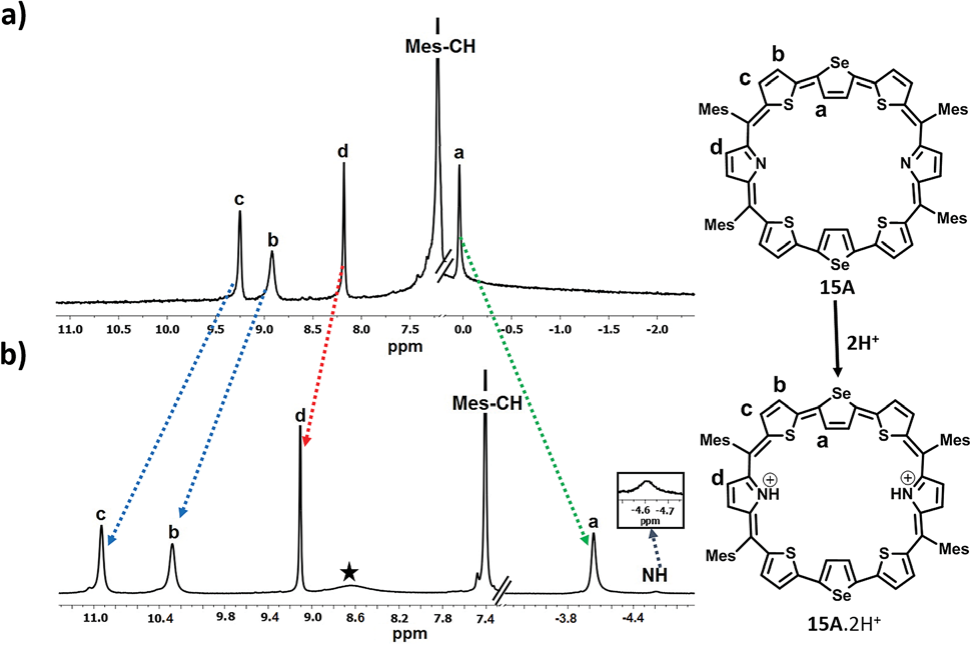
^1^H NMR spectrum: (a) **15A** (298 K) and (b) **15A**·2H^+^(298 K) in toluene D_8_.

In the case of **15**, since only one isomer, *i.e.* the centrally inverted symmetric isomer **A** was formed, and the ^1^H NMR spectrum was simple to interpret (Fig. S43 and S44, ESI†). Fig. 5 depicts the ^1^H NMR spectra of **15A** and its protonated derivative **15A**·2H^+^. The inverted selenophene β-CH protons resonate around 0.037 ppm confirming the inversion of the central selenophene rings. The thiophene ring protons (b and c) resonate as two doublets at 8.91 ppm and 9.25 ppm respectively, while the pyrrole β-CH protons appear as a singlet at 8.2 ppm. The mesityl –CH protons are at 7.22 ppm, while the methyl protons are in the expected region. Protonation of the pyrrole nitrogens leads to large shielding and deshielding of ring protons depending on the nature of the rings (Fig. S45 and S46, ESI†). The inverted selenophene β-CH protons experience a significant shielding of 4 ppm and appear at –4.04 ppm, suggesting that the selenophene rings come into the plane exposing the β-CH protons to the ring current of the macrocycle upon protonation.

The thiophene ring β-CH protons (b and c) experience deshielding (2.0 ppm for ‘c’ protons and 1.1 ppm for ‘b’ protons) and appear at 10.28 ppm and 10.93 ppm respectively. The pyrrole NH protons appear as a broad signal at –4.59 ppm, suggesting the aromatic nature of the macrocycle.

The difference in chemical shifts of most deshielded and most shielded ring protons (represented by Δδ) for various octaphyrin isomers listed in Table 1 gives some insight into the aromaticity of the macrocycles. The results are as follows: (i) **A** isomers have lower aromatic character relative to **B** isomers (Δδ = 8.97 *vs.* 12.11); (ii) protonated derivatives exhibit significantly larger Δδ values relative to freebase forms. The chemical shifts of inverted ring β-CH protons depend on the orientation of the inverted ring relative to the mean macrocyclic plane. In the **A** isomer, the centrally inverted heterocyclic rings are not completely planar due to which these protons experience only partial ring current. The terminally inverted rings in the **B** isomer are oriented in the plane of the macrocycle and hence these protons are more shielded relative to **A** isomer protons accounting for larger Δδ values. Upon protonation of pyrrole nitrogens, the inverted rings come into the plane of the macrocycle and the β-CH protons are completely exposed to the ring current of the macrocycle, which results in larger shielding of these protons. Hence, the protonated derivatives show larger aromaticity. Support for such a conclusion comes from the single crystal X-ray analysis of freebase and protonated derivatives of **10A** and **10B** (*vide infra*).

**Table 1:** Δδ values of octaphyrin isomers

Octaphyrin isomer	Δδ (ppm)	Octaphyrin isomer	Δδ (ppm)
**10A**	9.73	**13A**	9.06
**10B**	12.11	**13B**	11.25
**10B**·2H^+^	15.98	**13A**·2H^+^	17.36
		**13B**·2H^+^	18.14
**11A**	8.97		
**11B**	11.12	**15A**	9.21
**11B**·2H^+^	15.15	**15A**·2H^+^	15.52

### 2.3. NICS(0) calculations and AICD plots

**Fig. 6:**
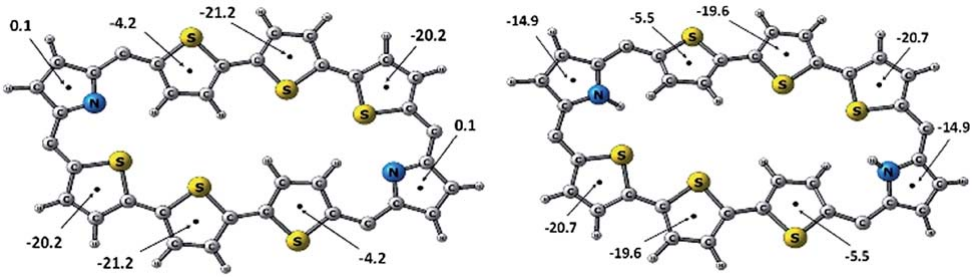
NICS(0) values (ppm) of (a) **10B** and (b) **10B**·2H^+^.

The NICS(0) values were calculated for all the individual heterocyclic rings of octaphyrins (10, 13 and 15) in freebase and protonated forms (Charts S1–S3, ESI†).^31^ It is observed in all the cases that the NICS(0) value for the pyrrole rings in the freebase form is 0.1 ppm, and those for inverted heterocyclic rings are from –3.3 ppm to –4.4 ppm, whereas those for the normal heterocyclic rings are between –18.1 ppm and –25.8 ppm, respectively, and the overall NICS(0) values are from –9.95 ppm to –12.04 ppm, suggesting the aromatic character in the freebase form. Upon protonation, the pyrrole rings have a higher NICS value as compared to the freebase form (–13.4 ppm to –15.2 ppm), whereas a nominal increase in that of the inverted heterocyclic rings (–4.2 ppm to –5.6 ppm) and marginal shift in that of the normal heterocyclic rings (–17.5 ppm to –20.6 ppm) with an overall increase in NICS values from –13.66 ppm to –15.18 ppm reveal that the protonated forms are more aromatic than the freebase form, justifying the conclusion drawn from ^1^H NMR chemical shift data. For example, the NICS value of **10B** and **10B**·2H^+^ is shown in Fig. 6 and found to be –11.4 ppm in **10B** and –15.17 ppm in **10B**·2H^+^, justifying that protonation enhances the aromatic character as compared to the freebase form.

Furthermore, the anisotropy induced current density plots (AICD plots) obtained at an isosurface value of 0.026 clearly suggest clockwise orientation of current density vectors indicating the presence of diatropic ring current in the octaphyrins (Charts S1–S3, ESI†).

### 2.4. Single crystal X-ray analysis

**Fig. 7:**
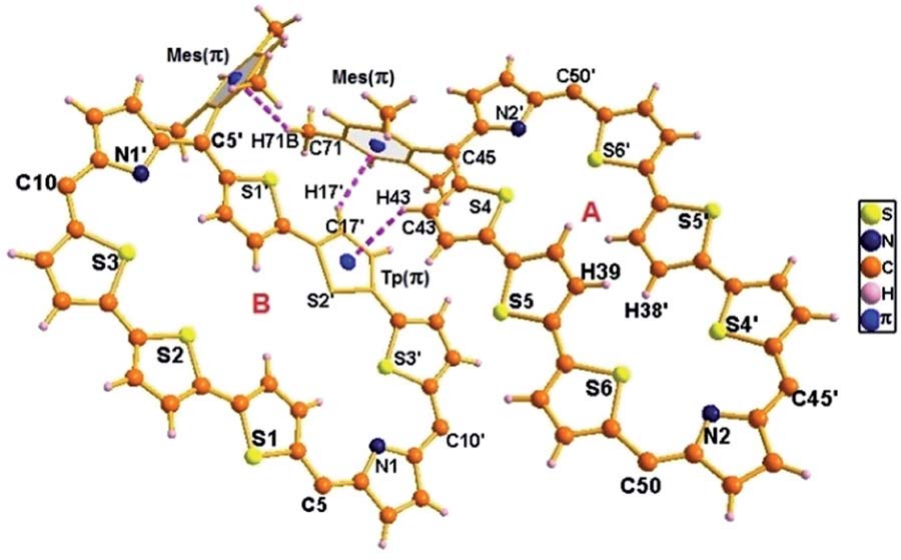
Single-crystal X-ray structure of **10**. The dotted line indicates intermolecular hydrogen bonding interactions. The *meso* mesityl substituents which are not involved in hydrogen bonding interactions are omitted for clarity.

The proposed structures of octaphyrin isomers were confirmed through single crystal X-ray analysis. The single crystal **10** obtained by slow diffusion of CH_2_Cl_2_ solution in *n*-hexane crystalizes in a monoclinic crystal lattice with the *P*2_1_/*n* space group.^32^ (Fig. S47–S49, Table S1, ESI†). As reflected in ^1^H NMR spectral analysis, both the isomers with the same empirical formula exist in the crystal lattice. Both the molecules contain two terthiophene units connected with two pyrrole units *via meso* carbon bridges. The only difference is in the inversion of one of the thiophene rings in the terthiophene units. The middle thiophene is inverted in **10A**, whereas in **10B**, one of the terminal rings is inverted (Fig. 7). The difference in the dihedral angle between the two planes (C5, C10, C5′ and C10′ *vs.* C45, C50, C45′ and C50′) is 38.84° (Fig. S47, ESI†). As reflected by the Δδ values (9.73 *vs.* 12.11) in Table 1, the terminally inverted thiophene rings (**10B**) are deviated by 16.27°, whereas the middle inverted thiophene rings are deviated by 21.23° (**10A**), confirming that the terminal rings are moving towards the plane and experiencing an effective ring current as compared to the middle thiophene rings. The maximum deviation of the middle thiophene ring is reflected in the steric repulsion between the inner core hydrogen atoms (H39···H38′). The *meso* aryl rings in **10** are almost perpendicular to the mean plane of the macrocycle (for **10A** 79.89°, 83.03°, 79.89°, and 83.03° and for **10B** 86.37°, 81.10°, 86.37°, and 81.10°) as observed in other *meso*-aryl expanded porphyrinoids.^33^ The crystal analyses of **10** revealed three different intermolecular hydrogen bonding interactions between (i) π-clouds of the inner thiophene (S2′) of **10B** and C43–H43 of **10A**, (ii) π-clouds of the *meso*-mesityl unit of **10A** and C17′–H17′ of **10B** and (iii) π-clouds of the *meso*-mesityl unit of **10B** and C71–H71B of **10A** and the bond distances and angles of S2′(π)···C43–H43, Mes(π)···C17′–H17′ and Mes(π)···C71–H71B are 2.83 Å & 136.14° and 2.71 Å & 137.01° and 3.15 Å & 150.91° respectively (Fig. S50 and S51, ESI†).

**Fig. 8:**
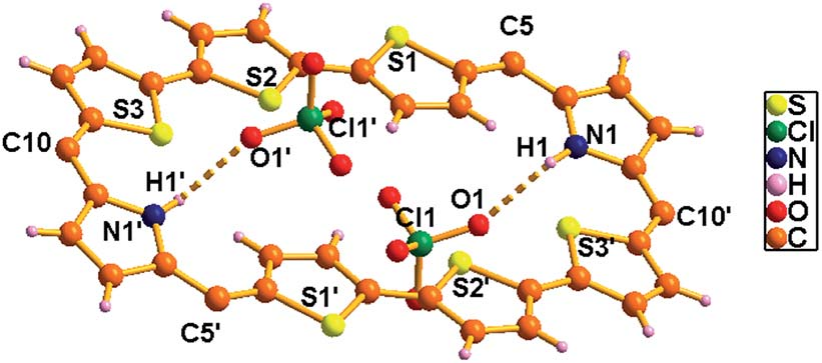
Single-crystal X-ray structure of **10B**·2H^+^. The dotted line indicates intermolecular hydrogen bonding interactions with ClO_4_^–^ ions. *Meso* mesityl substituents are omitted for clarity.

The exclusively stabilized **10B**·2H^+^ isomer upon protonation was unambiguously confirmed by single crystal X-ray analysis (Fig. 8). The molecule crystallizes in a triclinic lattice with the *P*1[combining macron] space group (Fig. S52–S54, Table S1, ESI†).^31^ Two perchlorate anions (O1/O1′) are involved in intermolecular hydrogen bonding interaction with the protonated imine NH (N1–H1/N1′–H1′) and the bond distance and angle of N1–H1···O1/N1′–H1′···O1′ are 2.33 Å and 118.41° respectively. These N–H···O bond length and bond angles compare well with those of a previously reported octaphyrin–TFA complex.^34^ The hydrogen bonding interactions generate a zig-zag one dimensional array in the solid state (Fig. S55, ESI†). The terminal thiophene unit (S1/S1′) is hardly deviated (12.99°) from the mean macrocyclic plane (C5, C10, C5′, and C10′) and maintains the planarity as observed in the freebase form of **10**. The deviation is lower as compared to **10B** (16.27°), proving the higher aromatic ring current in the terminal thiophene unit of the protonated form compared to the freebase form, as reflected by the higher Δδ value (15.98 *vs.* 12.11) in Table 1. By replacing counter anion from ClO_4_^–^ to SO_4_^2–^ ion, the crystal lattice contains **10B**·2H^+^ with one SO_4_^2–^ ion, where the SO_4_^2–^ ion generates zig-zag 1d array with intermolecular hydrogen bonding interaction with distance and angle of 2.17 Å and 129.55° (Fig. S56 and S57, ESI†).

**Fig. 9:**
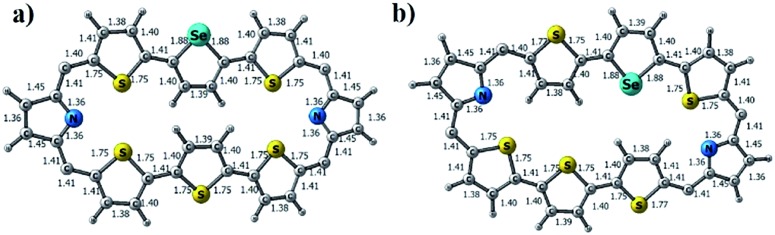
M06L/6-31G** level optimized geometry of (a) **13A** and (b) **13B**.

Repeated attempts to crystallize **13** failed in our hands. Hence, the structures of **13A** and **13B** were optimized at the M06L/6-31G** level. Fig. 9a and b show the optimized structures of the isomers of **13**. For **13A**, the middle selenophene and thiophene rings are inverted, where in **13B** the terminal thiophene rings are inverted, which are consistent with the ^1^H NMR data (Fig. S37–S40 in the ESI†), where similar ring inversions were observed. Upon protonation, both **13A**·2H^+^ and **13B**·2H^+^ were formed and the optimized structures of the protonated derivatives are given in Chart S2 in the ESI.†

**Fig. 10:**
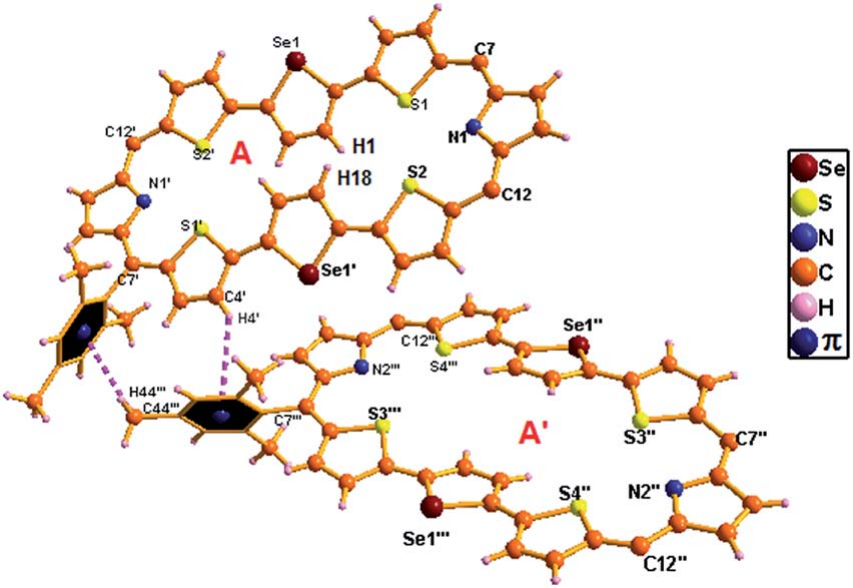
Single-crystal X-ray structure of **15A** with intermolecular hydrogen bonding interactions. The *meso* mesityl substituents which are not involved in hydrogen bonding interactions are omitted for clarity.

Octaphyrin **15** crystalizes in a monoclinic crystal lattice with the *P*2_1_/*c* space group (Fig. S58 and S59, Table S2, ESI†). As observed in **10**, two molecules of **15** are present in the single unit cell; however both are identical isomers in which only the middle selenophene ring is inverted (Fig. 10). The terminally inverted isomer **B** was not formed in the reaction. The difference in the dihedral angle between the two molecular planes (C7, C12, C7′, and C12′ *vs.* C7 C7ʺ, C12′′, C7′′′, and C12′′′) is 21.41°. The middle selenophene unit in , C12′′, C7′′′, and C12′′′) is 21.41°. The middle selenophene unit in **15A** is tilted by around 20.10° from the mean macrocyclic plane, whereas, the rest thiophene and pyrrolic rings are more or less planar with the mean molecular plane. The marginal difference in deviation (20.10° *vs.* 21.23°) of the middle selenophene unit over the middle thiophene unit (**10A**) adopts a similar trend to that observed in Δδ values (9.21 *vs.* 9.73) as shown in Table 1. Like **10A**, here also the repulsion between the inverted selenophene β-hydrogens (H1···H18) is responsible for maximum deviation. The *meso* mesityl groups are nearly perpendicular to the mean macrocyclic plane (78.30°, 76.37°, 78.30° and 76.37°). The structural analysis of **15A** revealed two weak intermolecular hydrogen bonding interactions between the two molecules, (i) Mes(π)···C4′–H4′ and (ii) Mes(π)···C44′′′–H44′′′ and the bond distances and angles were (i) 3.30 Å and 132.35° and (ii) 3.19 Å and 123.57° respectively (Fig. S60 and S61, ESI†).

**Fig. 11:**
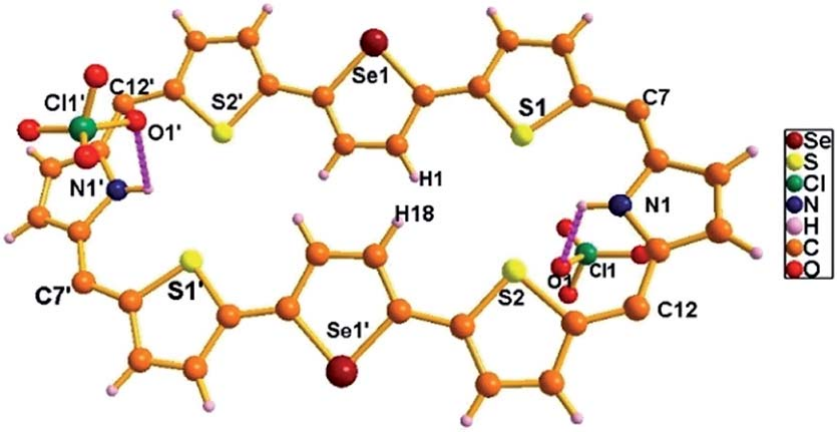
Single-crystal X-ray structure of **15A**·2H^+^. The dotted line indicates intermolecular hydrogen bonding interactions with two ClO_4_^–^ ions. *Meso* mesityl substituents are omitted for clarity.

The single crystal X-ray structure of **15A**·2H^+^ is shown in Fig. 11. The crystal was grown by slow evaporation of toluene in methanol at room temperature. The compound crystallizes in a triclinic crystal lattice with the *P*1[combining macron] space group and contains two ClO_4_^–^ ions (Fig. S62 and S63, Table S2, ESI†). The structural analysis reveals that the middle selenophene rings of 2,5-di(thiophen-2-yl)selenophene are inverted as in the freebase form. The inverted selenophene units deviate by 17.92° from the mean macrocyclic plane (C7, C12, C7′, and C12′) due to the steric repulsion between the β-CH protons (H1···H18) of the inverted selenophene moieties. The deviation in **15A**·2H^+^ is 3.48° less as compared to its freebase **15A**, further confirming the 4 ppm upfield shift of the inverted β-CH protons of **15A**·2H^+^ as revealed in ^1^H NMR spectroscopy. As reflected in **15A** the remaining heterocyclic rings are nearly coplanar (9.96° and 14.55°), whereas the *meso* mesityl substituents are nearly perpendicular (63.50°, 77.99°, 63.50° and 77.99°) to the mean macrocyclic plane. The two ClO_4_^–^ ions are located above and below the plane of the macrocycle with intermolecular hydrogen bonding interaction with the protonated imine NH's (N1–H1/N1′–H1′) and the bond distance and angle of N1–H1···O1/N1′–H1′···O1′ are 2.54 Å and 107.83° respectively.

### 2.5. DFT calculations

The exclusive formation of a particular isomer relative to the other suggests a small energy difference between the isomers. Any small external perturbation can alter this difference in energy and favour one isomer over the other. Keeping this in mind, we have calculated single point energies on the optimized structures of isomers in freebase and protonated forms using the M06L/CC-pVTZ//M06L/6-31G** level of theory. Table S3† summarizes the energies calculated. For example, the table shows that **10A** is more stable than **10B** by 2 kcal mol^–1^. However, **10B**·2H^+^ is found to be more stable than **10A**·2H^+^ by 0.5 kcal mol^–1^. Furthermore the protonation is found to have a more stabilizing effect on **10B**·2H^+^ relative to **10A**·2H^+^ due to increased aromaticity. In addition, the calculations do not take into account the effect of counter anions and crystal packing forces. Taken together these observations explain the stabilization of **10B**·2H^+^ relative to **10A**·2H^+^. In the case of **13**, **13A** is more stable by 3.4 kcal mol^–1^ relative to **13B**. Upon protonation, these energy differences between the two isomers remain the same and protonation effects are similar in both **A** and **B** isomers, explaining the formation of both isomers **13A**·2H^+^ and **13B**·2H^+^ upon protonation. In the case of **15**, the calculations showed that **15A** is more stable than **15B** by 4.8 kcal mol^–1^, supporting the formation of only isomer **15A**. Even in the protonated state **15A**·2H^+^ is found to be more stable than **15B**·2H^+^ by 5.9 kcal mol^–1^, justifying the formation of **15A**·2H^+^ upon protonation.

## 3. Conclusions

In conclusion, syntheses and spectral and structural characterization of four 34π core modified octaphyrins have been described. Spectral and X-ray structural studies indicate that octaphyrins exhibit rotational isomers and the structure of the isomer depends on the nature of the heteroatom present in the core of the macrocycle. The octaphyrins are aromatic both in freebase and protonated forms. In the protonated form, two counter anions (ClO_4_^–^ and SO_4_^2–^) bind to the macrocycle through N–H···O hydrogen bonding interaction and anions are found above and below the macrocyclic plane. The NICS(0) values and AICD plots satisfactorily explain the aromaticity and the presence of diatropic ring current in both freebase and protonated forms. DFT calculations support the exclusive formation of a particular isomer upon protonation in terms of stabilization energies. Further studies on their excited state properties and nonlinear optical behaviour by the two photon absorption technique are in progress to exploit their diverse chemistry.

## Conflicts of interest

There are no conflicts to declare.

## Supplementary Material

Supplementary information

Crystal structure data
