# Supplementary material for: Two non-identical twins in one unit cell: characterization of 34π aromatic core-modified octaphyrins, their structural isomers and anion bound complexes
Source: Chem Sci. 2019 May 15;10(23):5911–9. doi: 10.1039/c9sc01633c (PMC6582758; doi:10.1039/c9sc01633c)
Supplement: Supplementary file 1 [file SC-010-C9SC01633C-s001.pdf]

Electronic Supplementary Material (ESI) for Chemical Science.
This journal is © The Royal Society of Chemistry 2019

Supporting Information

for

Two Non-identical twins in one Unit Cell: Characterization of 34π Aromatic Core-modified Octaphyrins, their structural isomers and anion bound complex

Arindam Ghosh,[a‡] Syamasrit Dash,[a‡] A. Srinivasan,[a] Cherumuttathu H. Suresh,[b] and Tavarekere K. Chandrashekar[*,a]

[a]School of Chemical Sciences, National Institute of Science Education and Research (NISER), Homi Baba National Institute (HBNI), Bhubaneswar-752050, Odisha, India.

E-mail: tkc@niser.ac.in

[b]Inorganic and theoretical chemistry section, Chemical Sciences and Technology Division, CSIR-National Institute of Interdisciplinary Science and Technology, Trivandrum - 695019, Kerala, India

‡These authors contributed equally.

Table of Contents:

1. General Information : 2-3
2. Syntheses : 4-8
3. Mass spectral analysis of **10**, **11**, **13** and **15** : 9-11
4. Electronic spectral analysis of **10**, **10**.$2H^+$, **11**, **11**.$2H^+$,**13**, **13**.$2H^+$ and **15**, **15**.$2H^+$: 12-19
5. NMR spectral analysis of **10**, **10**.$2H^+$, **11** and **11**.$2H^+$, **13**, **13**.$2H^+$ and **15**, **15**.$2H^+$: 20-38
6. DFT, NICS (0) and AICD plots of **10**, **10**.$2H^+$, **13**, **13**.$2H^+$ and **15**, **15**.$2H^+$: 39-43
7. Single crystal X-ray structure and analysis of **10**, **10**.$2H^+$, **15** and **15**.$2H^+$: 44-58
8. Energy table and Cartesian coordinates of optimized geometries : 59-120

General Information:

The solvents required for the synthesis, such as Tetrahydrofuran, Dichloromethane, *n*-Hexane were purified by using standard procedure. Deuterated NMR solvent (Toluene-D_8 and CD_2Cl_2) were used as received. All NMR spectra were recorded with Bruker 400 MHz and 500 MHz spectrometer in solvent Toluene-D_8 using tetramethylsilane (TMS) as an internal standard. Chemical shifts are expressed in parts per million (ppm) units relative to TMS. Electron spray ionization (ESI) mass spectra were recorded on Bruker, micrOTOF-QII mass spectrometer. Electronic spectra were recorded with Perkin Elmer – Lambda 750 UV – winlab software package. X-ray quality crystals for the compounds were grown by the slow diffusion of acetonitrile over $CHCl_3$ (**10**) and $CHCl_3$ over hexane (**10B**.$2H^+$). Single crystal X-ray diffraction data were collected on a Bruker KAPPA APEX-II, four angle rotation system and MoKα radiation (0.71073 Å).

DFT Studies: NICS (0) and AICD plots

Density functional theoretical (DFT) studies have been conducted using the M06L/6-31G** level of theory[1] using Gaussian09 suite of programs.[2] All the octaphyrins in the free base and doubly protonated forms are optimized using the DFT method. Further optimized geometries are confirmed as energy minima by vibrational frequency calculation (all show zero imaginary frequency). Moreover, in order to obtain more accurate energy values, single point energy calculation is done on optimized structures using M06L/CC-pVTZ level of theory. Thus all the reported energy values are at M06L/CC-pVTZ//M06L/6-31G** level. Nucleus independent chemical shift values at the ring center (NICS(0))[3] is calculated using the Gauge-Independent Atomic Orbital (GIAO) method as implemented in Gaussian09.[4] Anisotropy of the current (induced) density (AICD) plots are calculated using the AICD program developed by Herges and Geuenich[5].

[1]. Y. Zhao, D. G. Truhlar, *J. Chem. Phys*. **2006**, *125*, 194101.

[2]. Gaussian 09, Revision D.01, M. J. Frisch, G. W. Trucks, H. B. Schlegel, G. E.Scuseria, M. A. Robb, J. R. Cheeseman, G. Scalmani, V. Barone, B. Men-nucci, G. A. Petersson, H. Nakatsuji, M. Caricato, X. Li, H. P. Hratchian, A. F. Izmaylov, J. Bloino, G. Zheng, J. L. Sonnenberg, M. Hada, M. Ehara, K. Toyota, R. Fukuda, J. Hasegawa, M. Ishida, T. Nakajima, Y. Honda, O. Kitao, H. Nakai, T. Vreven, J. A. Montgomery, Jr., J. E. Peralta, F. Ogliaro, M. Bearpark, J. J. Heyd, E. Brothers, K. N. Kudin, V. N. Staroverov, R. Ko-bayashi, J. Normand, K. Raghavachari, A. Rendell, J. C. Burant, S. S. Iyen-gar, J. Tomasi, M. Cossi, N. Rega, J. M. Millam, M. Klene, J. E. Knox, J. B. Cross, V. Bakken, C. Adamo, J. Jaramillo, R. Gomperts, R. E. Stratmann, O. Yazyev, A. J. Austin, R. Cammi, C. Pomelli, J. W. Ochterski, R. L. Martin, K. Morokuma, V. G. Zakrzewski, G. A. Voth, P. Salvador, J. J. Dannenberg, S. Dapprich, A. D. Daniels, Ö. Farkas, J. B. Foresman, J. V. Ortiz, J. Cio-slowski, D. J. Fox, Gaussian, Inc., WallingfordCT, 2009

[3]. P. von R. Schleyer, C. Maerker, A. Dransfeld, H. Jiao, N. J. R. van Eikema Hommes, *J. Am. Chem. Soc.* **1996**, *118*, 6317.

[4]. K. Wolinski, J. F. Hilton, P. Pulay, *J. Am. Chem. Soc*., **1990**, 112, 8251.

[5]. R. Herges, D. Geuenich, *J. Phys. Chem. A* **2001**, *105*, 3214.

Syntheses:

Scheme 1:

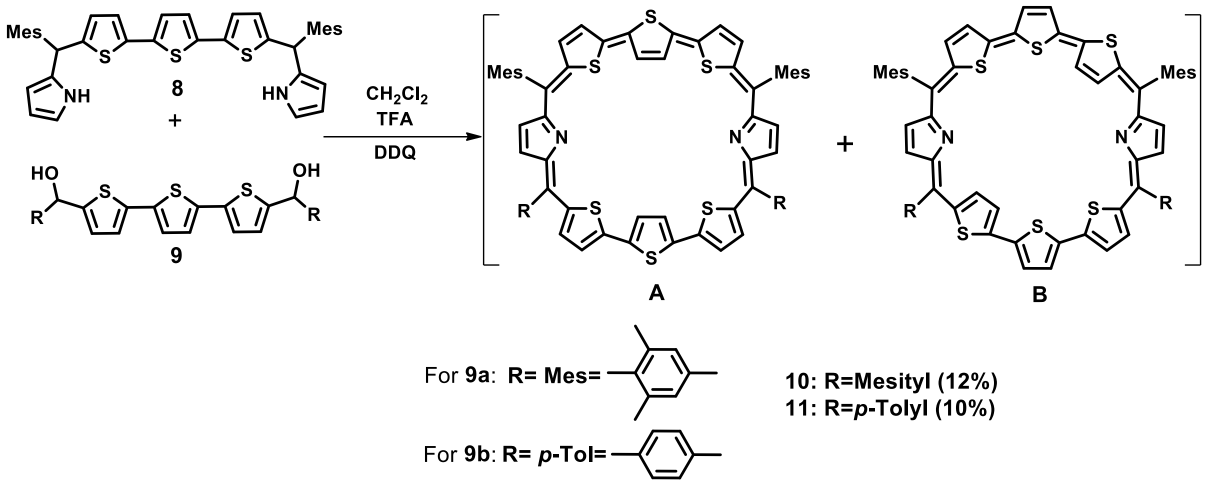

A mixture of terthiophene pentapyrrane (200 mg, 0.31mmol) (**8**) and terthiophene mesityl diol (169 mg, 0.31 mmol) (**9a**) were dissolved in 200 ml of dry CH_2Cl_2 and the resulting solution was stirred under nitrogen atmosphere for 15 min. Trifluoroacetic acid (24 μl, 0.31 mmol) was added and the resulting solution was stirred for 1h. The progress of the reaction was monitored by TLC. DDQ (212 mg, 0.93 mmol) was added and the resulting solution was stirred for another 1h. The solvent was evaporated in rotary evaporator. The residue was purified by basic alumina followed by silica gel (100-200 mesh) column chromatography. The dark blue color band was eluted with CH_2Cl_2/*n*-hexane (55:45, v/v) and identified as octaphyrin (**10**) in 12% yield. Recrystallization with CH_2Cl_2/CH_3OH gave bronze color crystalline product.

Compound 10: ^{1}H NMR (400 MHz, Toluene-d_8) δ (in ppm) = 9.85 (d, 1H), 9.83 (d, 1H), 9.76 (d, 1H), 9.21 (s, 5H), 8.60 (s, 1H), 8.58 (d, 1H), 8.49 (d, 1H), 8.06 (s, 4H), 7.39 (s, 2H), 7.25 (s, 2H), 7.16 (s, 8H), 2.60 (s, 3H), 2.53 (s, 3H), 2.46 (s, 12H), 2.34 (s, 6H), 2.16 (s, 6H), 2.12 (s, 12H), 0.37 (s, 3H), -1.26 (s, 1H); **10**.2H$^+$: ^{1}H NMR (400 MHz, Toluene-d_8) δ (in ppm) = 11.28 (d, 1H), 11.16 (d, 1H), 11.07 (d, 1H), 10.47 (d, 1H), 9.34 (d, 1H), 9.29 (d, 1H), 7.61 (s, 2H), 7.45 (s, 2H), 2.75 (s, 3H), 2.67 (s, 3H), 2.59 (s, 2H), 2.31 (s, 6H), 2.01 (s, 4H), -2.76 (s,

1H, NH), -4.29 (d, 1H), -4.70 (d, 1H). **10**: UV/Vis (CH_2Cl_2): λ_{max} in nm (ε in $dm^3mol^{-1}cm^{-1}$) = 600 (8.65×10^4), 647 (1.10×10^5), 905 (7.45×10^4); **10·2H⁺** (TFA/CH_2Cl_2): λ_{max} in nm (ε in $dm^3mol^{-1}cm^{-1}$) = 633 (2.10×10^5), 1116 (9.80×10^4).

Synthesis of 11

Terthiophene mesityl pentapyrrane (200 mg, 0.34 mmol) (**8**), terthiophene tolyl diol (185 mg, 0.34 mmol) (**9b**) and Trifluoroacetic acid (26 µl, 0.34 mmol) were dissolved in 200 ml of dry CH_2Cl_2 under similar condition as mentioned in **10**. After DDQ (232 mg, 1.02 mmol) oxidation, the crude residue was purified by basic alumina followed by silica gel (100-200 mesh) column chromatography. The dark blue color band was eluted with CH_2Cl_2/*n*-hexane (58:42, v/v) and identified as octaphyrin (**11**) in 10% yield. Recrystallization with CH_2Cl_2/CH_3OH gave bronze color crystalline product.

Compound 11: ^1H NMR (400 MHz, Toluene-d_8) δ (in ppm) = 9.88-9.80 (m, 3H), 9.31 (s, 2H), 9.21 (s, 2H), 8.74 (d, 1H), 8.63 (d, 1H), 8.53 (s, 1H), 8.34 (d, 1H), 8.27 (s, 2H), 8.13 (s, 3H), 7.88 (s, 4H), 7.59 (d, 2H), 7.44 (d, 3H), 7.34 (s, 4H), 7.24 (s, 4H), 2.54 (s, 6H), 2.45 (s, 6H), 2.22 (s, 12H), 2.13 (s, 25H), 0.48 (m, 4H), -1.10 (d, 1H), -1.24 (d, 1H). **11.2H⁺**: ^1H NMR (400 MHz, Toluene-d_8) δ (in ppm) = 11.06-10.83 (m, 5H), 10.61 (s, 1H), 10.26 (d, 1H), 10.12 (d, 1H), 9.19 (d, 2H) 9.16 (d, 1H), 9.09 (d, 2H), 8.99 (d, 2H), 8.88 (d, 2H), 8.69 (d, 1H), 8.59 (d, 1H), 8.44 (d, 1H), 7.88 (d, 2H), 7.76 (d, 2H), 7.63 (s, 1H), 7.59 (s, 2H), 7.44 (s, 2H), 7.34 (s, 1H), 2.73 (s, 3H), 2.67 (s, 6H), 2.63 (s, 3H), 2.30 (s, 6H), 2.03 (s, 6H), -1.12 (s, 1H, NH), -3.12 (d, 1H), -3.27 (s, 1H, NH), -3.73 (d, 1H), -3.86 (d, 1H), -4.09 (d, 1H). **11**: UV/Vis (CH_2Cl_2): λ_{max} in nm (ε in $dm^3mol^{-1}cm^{-1}$) = 602 (8.58×10^4), 650 (1.03×10^5), 917 (7.32×10^4); **11·2H⁺** (TFA/CH_2Cl_2): λ_{max} in nm (ε in $dm^3mol^{-1}cm^{-1}$) = 634 (2.07×10^5), 1153 (8.95×10^4).

Scheme 2:

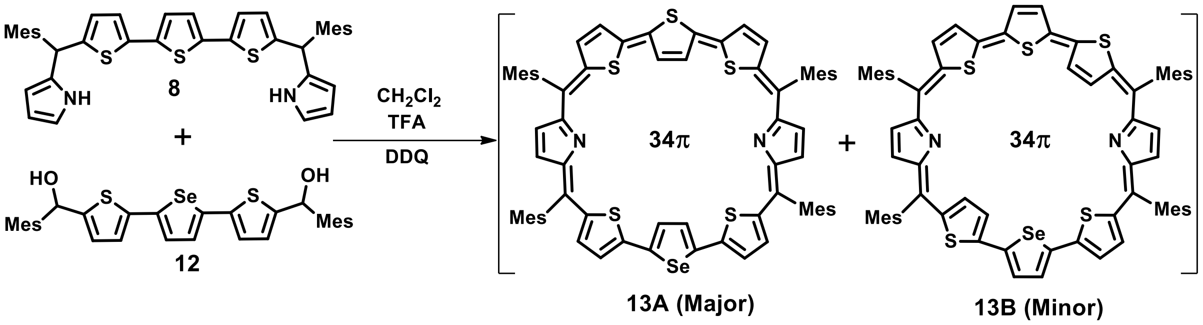

Pentapyrrane (200 mg, 0.31 mmol) (**8**), diol (184 mg, 0.31 mmol) (**12**) and Trifluoroacetic acid (24μl, 0.31 mmol) were dissolved in 200 ml of dry CH_2Cl_2 under similar condition as mentioned in **11**. After DDQ (213 mg, 0.93 mmol) oxidation, the crude residue was purified by basic alumina followed by silica gel (100-200 mesh) column chromatography. The dark blue color band was eluted with CH_2Cl_2/*n*-hexane (58:42, v/v) and identified as Octaphyrin (**13**) in 9% yield. Recrystallization with CH_2Cl_2/CH_3OH gave bronze color crystalline product. **Compound 13**: ^1H NMR (400 MHz, Toluene-d_8) δ (in ppm) = 10.03 (d, 1H), 9.93 (d, 1H), 9.81 (d, 2H), 9.71 (d, 2H), 9.20 (d, 2H), 9.06 (d, 2H), 8.78-8.73 (m, 4H), 8.56 (d, 1H), 8.48 (d, 2H), 8.42 (d, 1H), 8.08 (d, 2H), 8.02 (d, 2H), 7.37 (s, 4H), 7.23 (s, 4H), 7.15 (s, 8H), 2.58 (s, 3H), 2.52 (s, 3H), 2.45 (s, 12H), 2.32 (s, 6H), 2.11 (s, 12H), 0.49 (s, 3H), 0.14 (s, 3H), -1.01 (s, 1H), -1.22 (s, 1H); **13**.$2H^+$: ^1H NMR (400 MHz, Toluene-d_8) δ (in ppm) = 11.60 (d, 1H), 11.47-11.45 (m, 2H), 11.29 (d, 1H), 11.19 (d, 1H), 11.03 (d, 1H), 10.82-10.80 (m, 4H), 10.47 (d, 1H), 10.37 (d, 1H), 10.16-10.13 (m, 4H), 9.40 (d, 1H), 9.35 (d, 1H), 9.30-9.28 (m, 2H), 9.02 (d, 2H), 8.99 (d, 1H), 7.62 (s, 2H), 7.60 (s, 2H), 7.46 (s, 4H), 7.37 (s, 8H), 2.76 (s, 6H), 2.68 (s, 3H), 2.63 (s, 12H), 2.25 (s, 6H), 2.07 (s, 12H), 2.00 (s, 6H), -3.35 (s, 4H), -4.02 (s, 4H), -4.13 (d, 1H), -4.36 (d, 1H), -4.73 (d, 1H), -4.92 (d, 1H), -6.54 (NH, br). **13**: UV/Vis (CH_2Cl_2): λ_{max} in nm (ε in $dm^3mol^{-1}cm^{-1}$) = 601 (8.54×10^4), 651 (9.48×10^4), 910 (7.65×10^4); **13·2H⁺** (TFA/CH_2Cl_2): λ_{max} in nm (ε in $dm^3mol^{-1}cm^{-1}$) = 637 (2.35×10^5), 1118 (1.30×10^5),

Scheme 3:

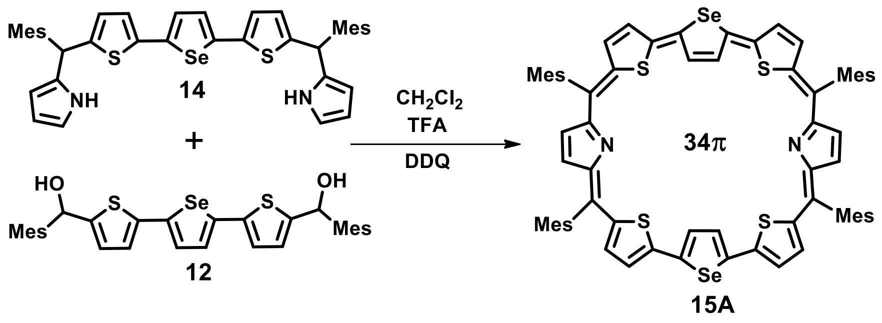

Pentapyrrane (200 mg, 0.29mmol) (**14**), diol (171 mg, 0.29mmol) (**12**) and Trifluoroacetic acid (22µl, 0.29mmol) were dissolved in 200 ml of dry CH_2Cl_2 under similar condition as mentioned in **13**. After DDQ (165 mg, 0.80 mmol) oxidation, the crude residue was purified by basic alumina followed by silica gel (100-200 mesh) column chromatography. The dark blue color band was eluted with CH_2Cl_2/*n*-hexane (52:48, v/v) and identified as Octaphyrin (**15**) in 10% yield. Recrystallization with CH_2Cl_2/CH_3OH gave bronze color crystalline product.

Compound 15: ^{1}H NMR (400 MHz, Toluene-d_8) δ (in ppm) = 9.25 (d, 4H), 8.91 (d, 4H), 8.18 (s, 4H), 7.22 (s, 8H), 2.52 (s, 12H), 2.16 (s, 24H), 2.12 (s, 12H), 0.037 (s, 3H); **15**.2H$^+$: ^{1}H NMR (400 MHz, Toluene-d_8) δ (in ppm) = 10.93 (d, 4H), 10.28 (d, 4H), 9.11 (s, 4H), 7.41 (s, 8H), 2.67 (s, 12H), 1.97 (s, 24H), -4.04 (s, 4H), -4.59 (NH, br). **15**: UV/Vis (CH_2Cl_2): λ_{max} in nm (ε in dm^{3}mol^{-1}cm^{-1}) = 605 (8.48×10^4), 654 (7.74×10^4), 913 (6.18×10^4); **15·2H$^+$** (TFA/CH_2Cl_2): λ_{max} in nm (ε in dm^{3}mol^{-1}cm^{-1}) = 642 (2.09×10^5), 1116 (1.24×10^5).

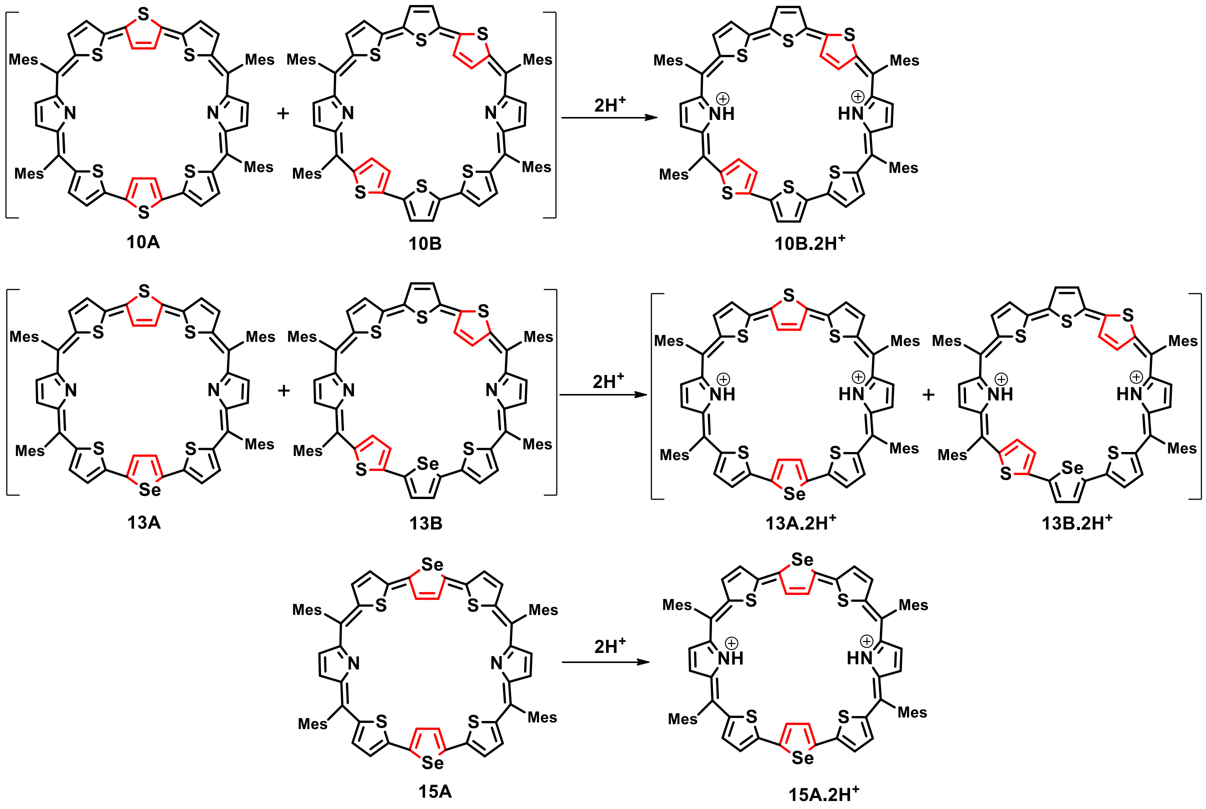

Scheme S4: Proton triggered stabilization of octaphyrin isomers **10**, **13** and **15**

Mass spectral analysis of **10**, **11, 13 and 15**

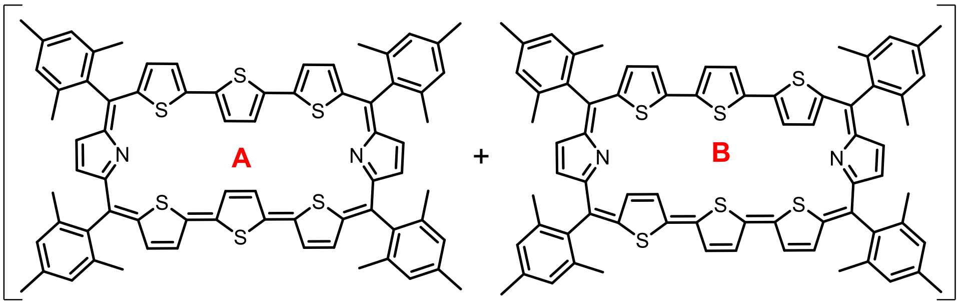

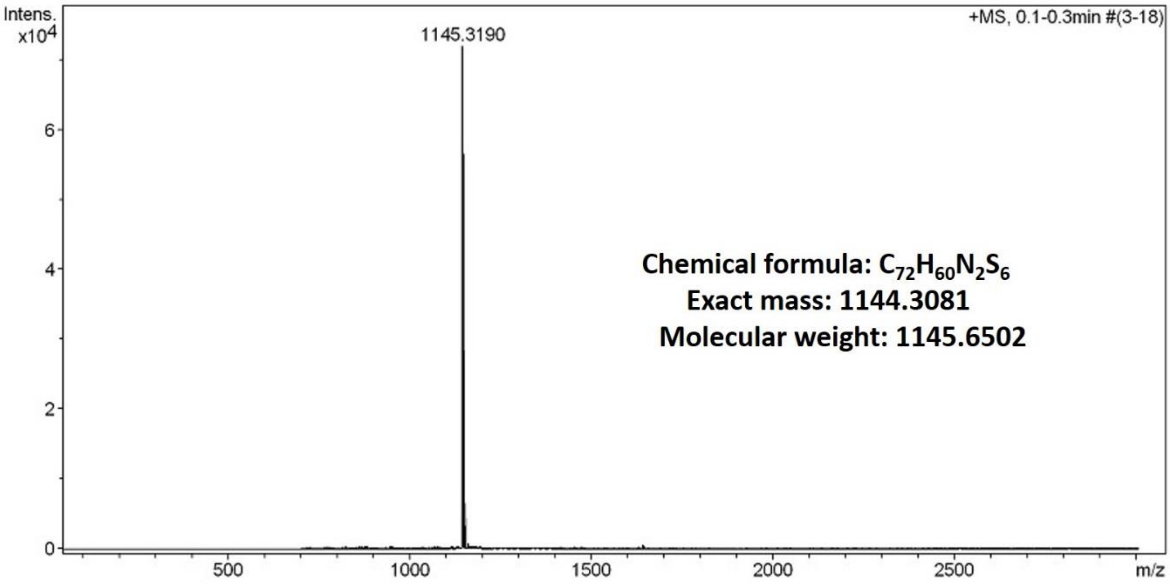

Figure S1: ESI-Mass spectrum of **10**.

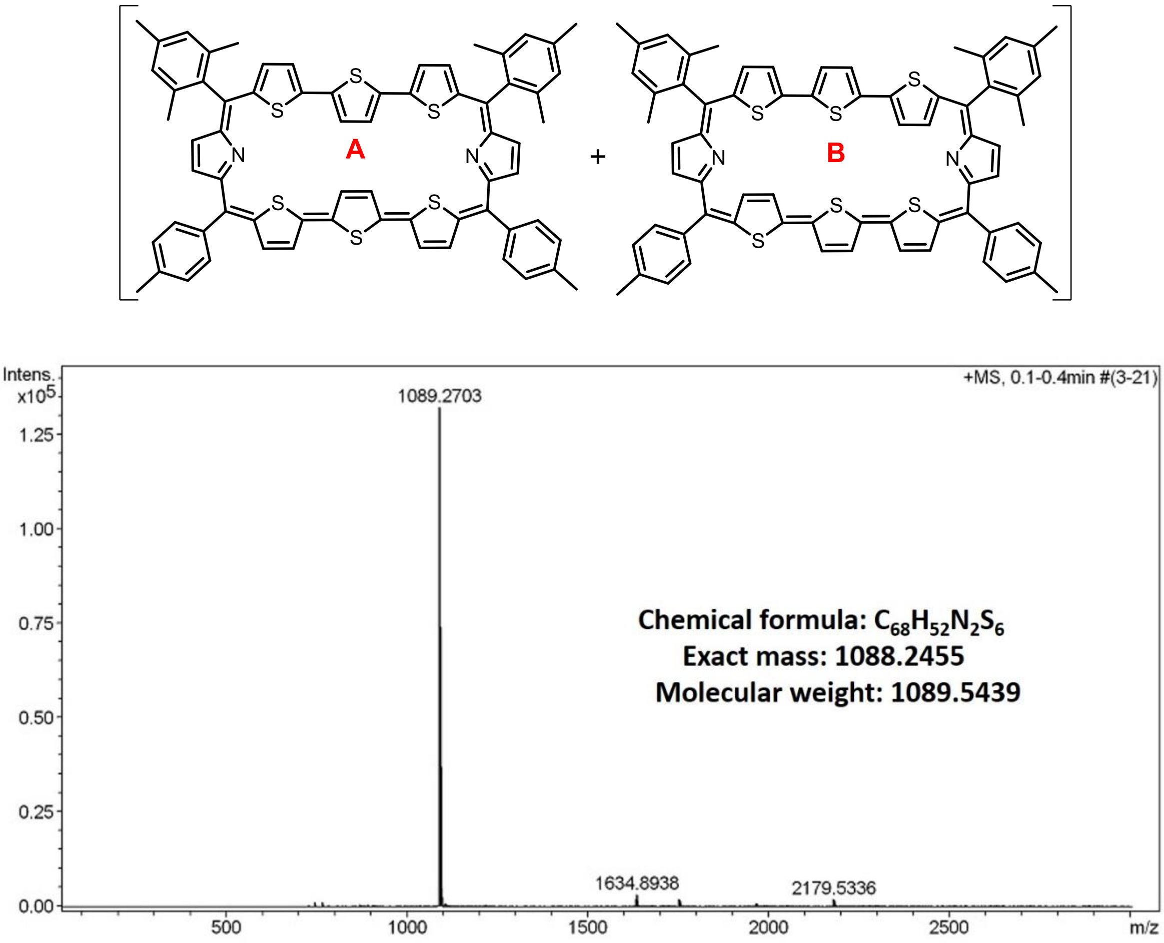

Figure S2: ESI-Mass spectrum of **11**.

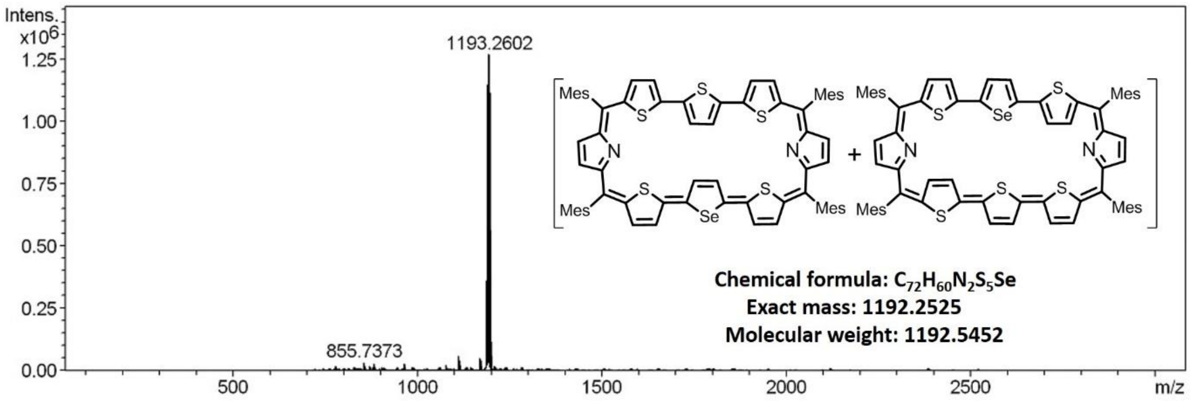

Figure S3: ESI-Mass spectrum of **13**.

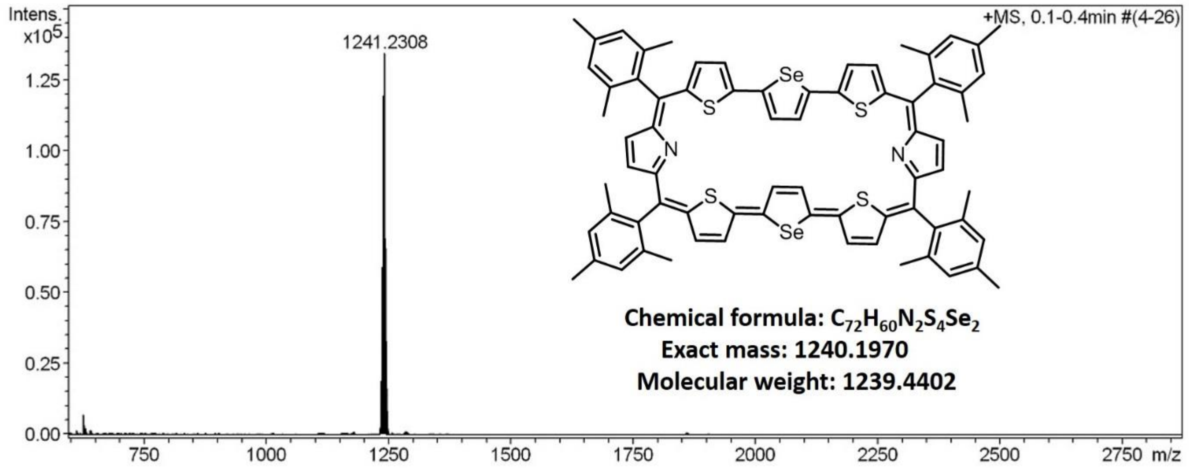

Figure S4: ESI-Mass spectrum of **15**.

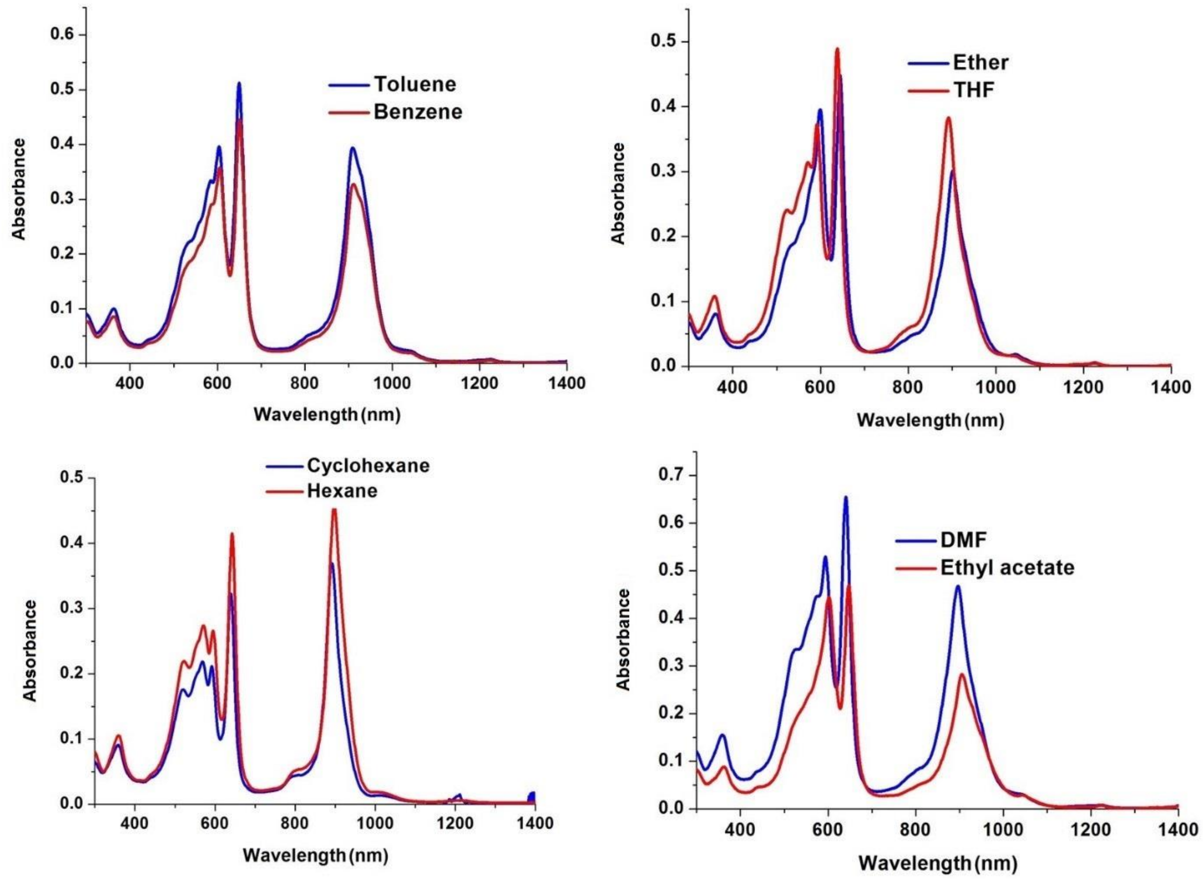

Figure S5: Electronic absorption spectra of **10** in various solvents.

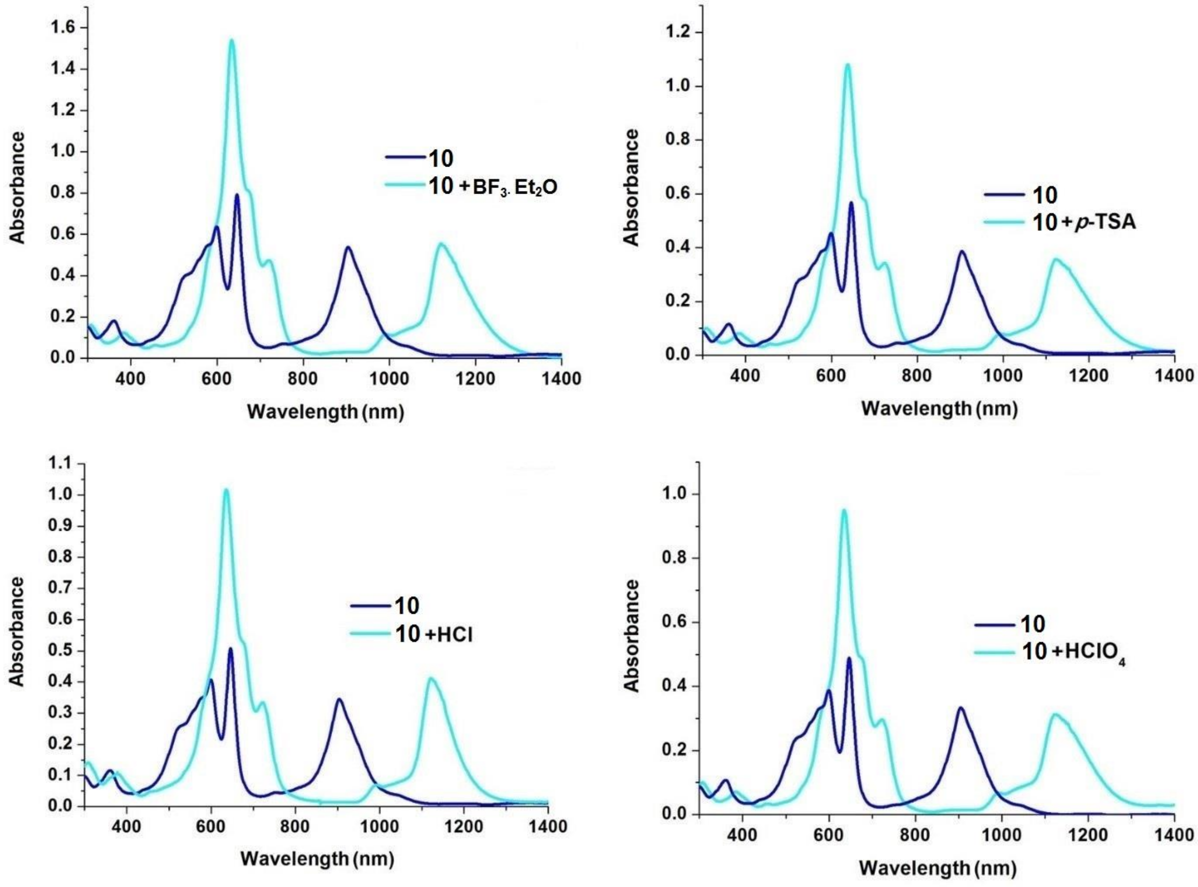

Figure S6: UV-Vis spectrum of **10** in various acids.

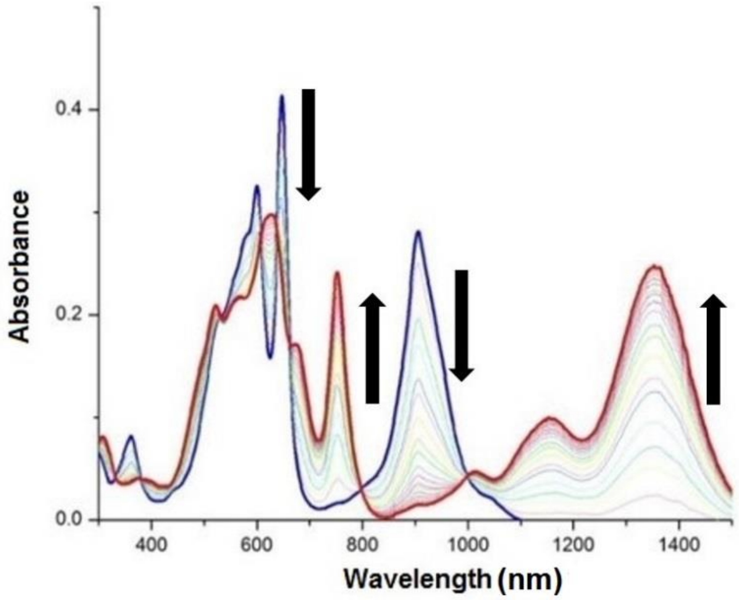

Figure S7: The electronic absorption spectra of **10** in CH_2Cl_2 with less acid concentration from 10^{-8} M to 10^{-6} M.

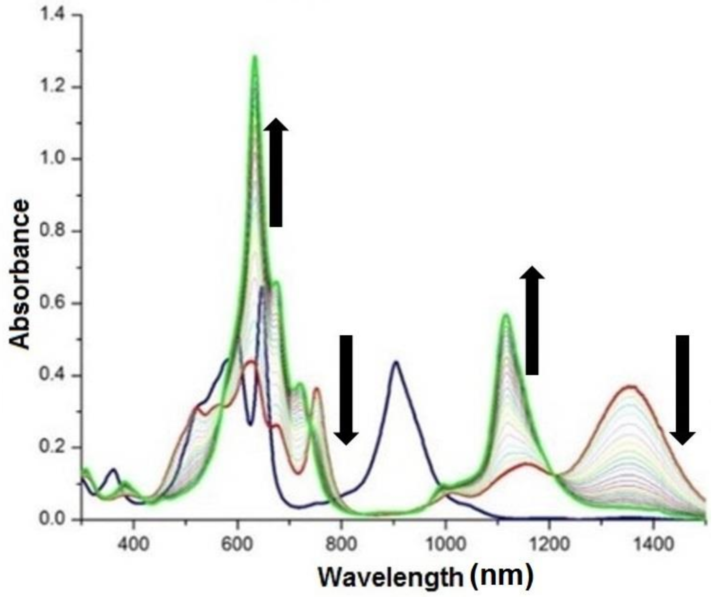

Figure S8: The electronic absorption spectra of **10** in CH_2Cl_2 with high acid concentration from 10^{-6} M to 10^{-2} M.

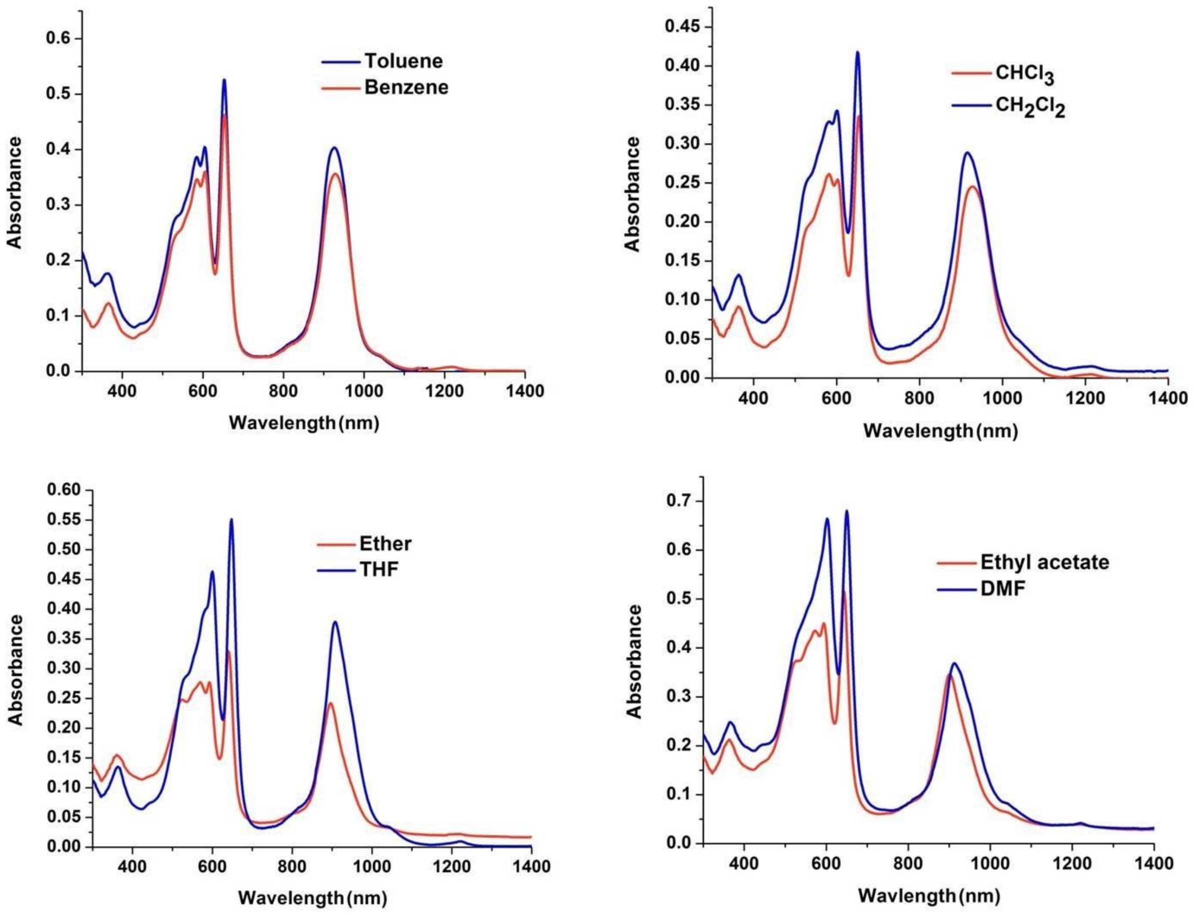

Figure S9: Electronic absorption spectra of **11** in various solvents.

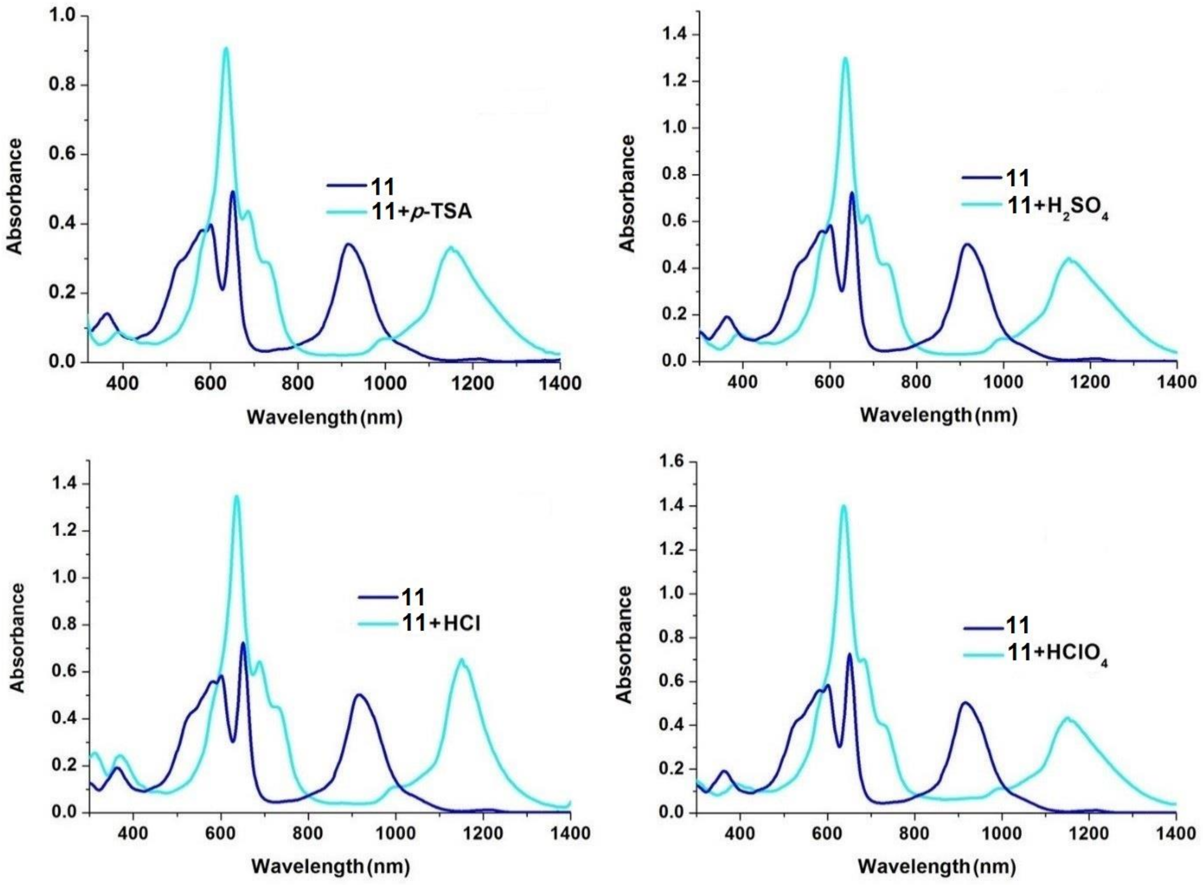

Figure S10: UV-Vis spectrum of **11** in various acids.

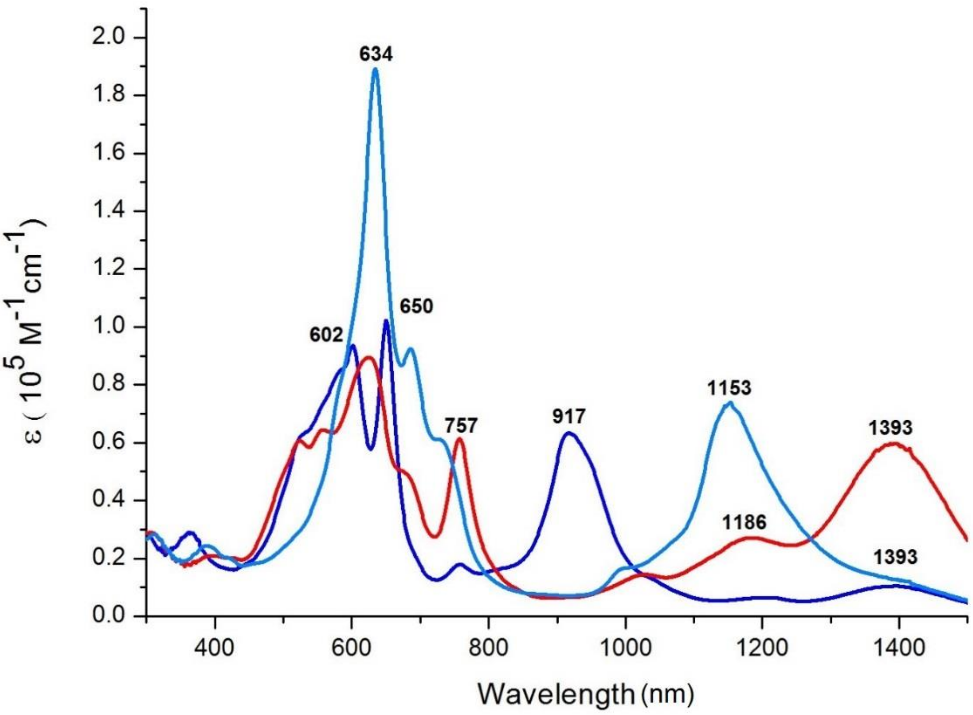

Figure S11: The electronic absorption spectra of **11** and **11**.$2H^{+}$in CH_2Cl_2.

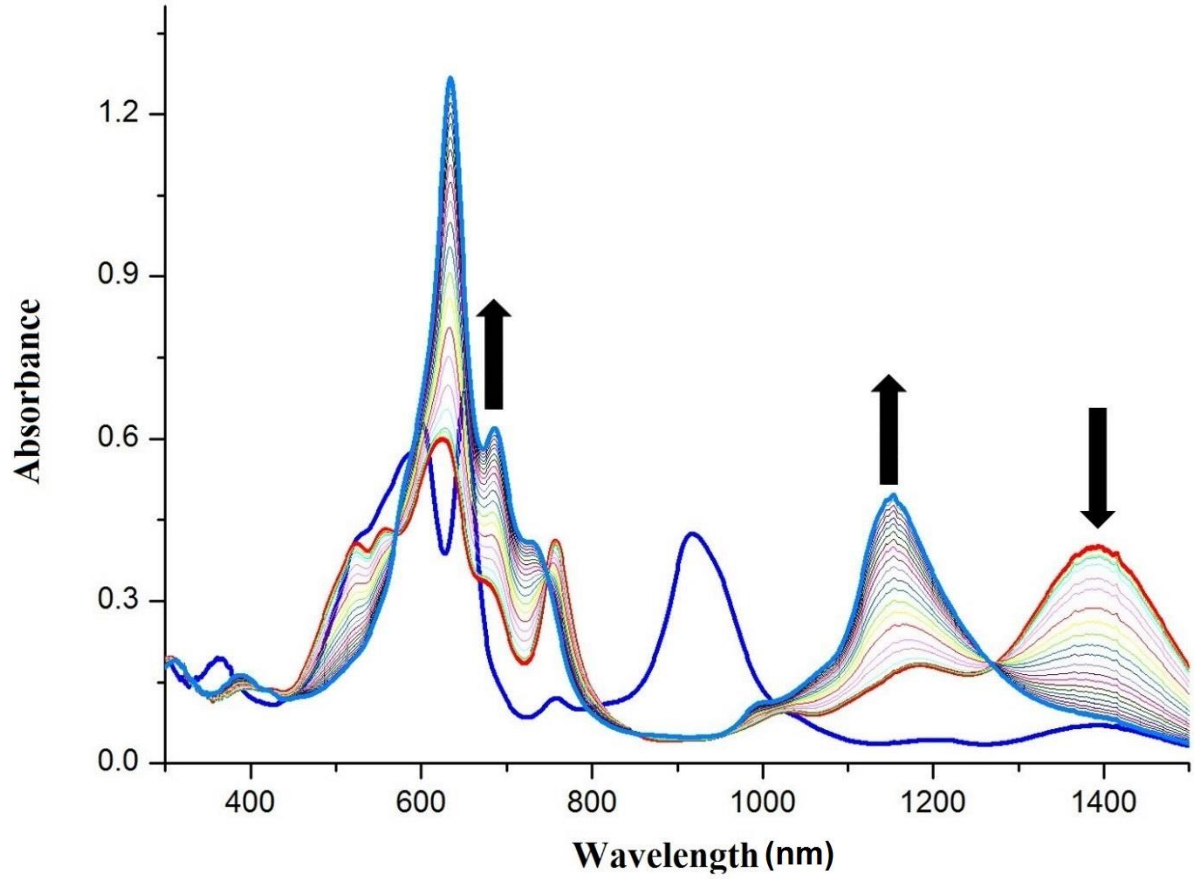

Figure S12: The electronic absorption spectra of **11** in CH_2Cl_2 with varying concentration of TFA (10^{-8} M to 10^{-2} M).

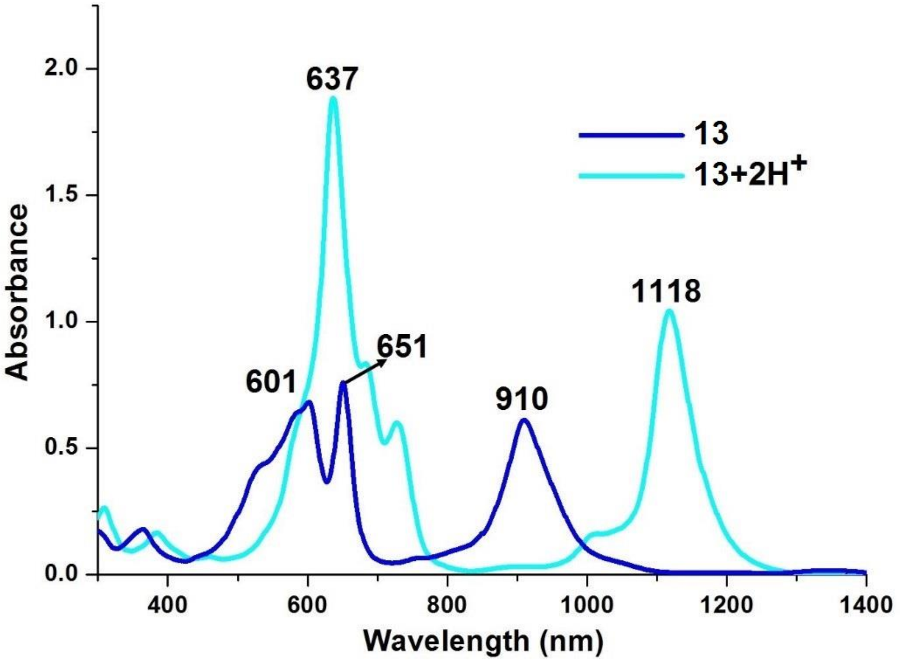

Figure S13: The electronic absorption spectra of **13** and **13**.2H$^+$in CH_2Cl_2.

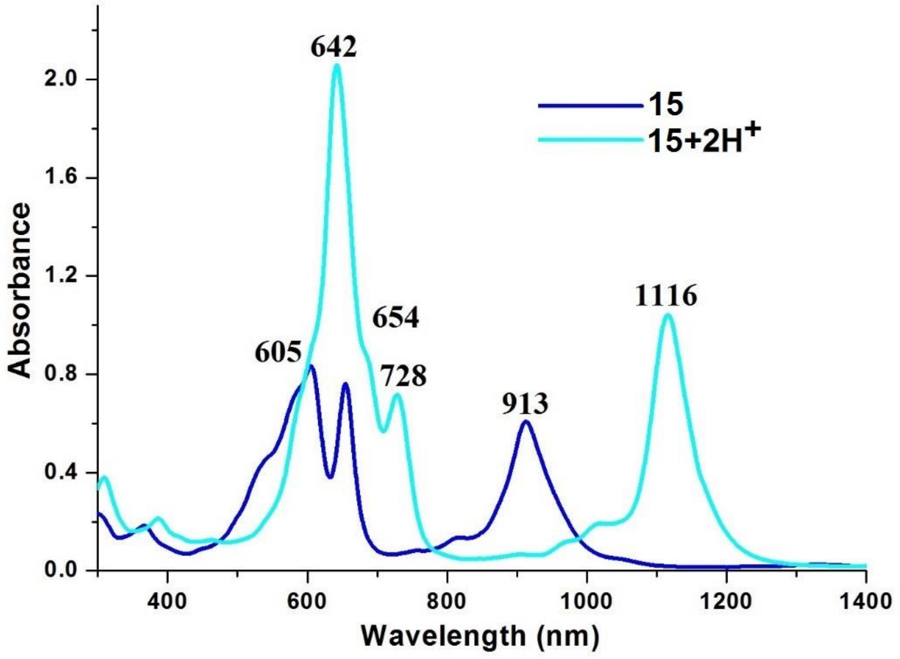

Figure S14: The electronic absorption spectra of **15** and **15**.2H$^+$in CH_2Cl_2.

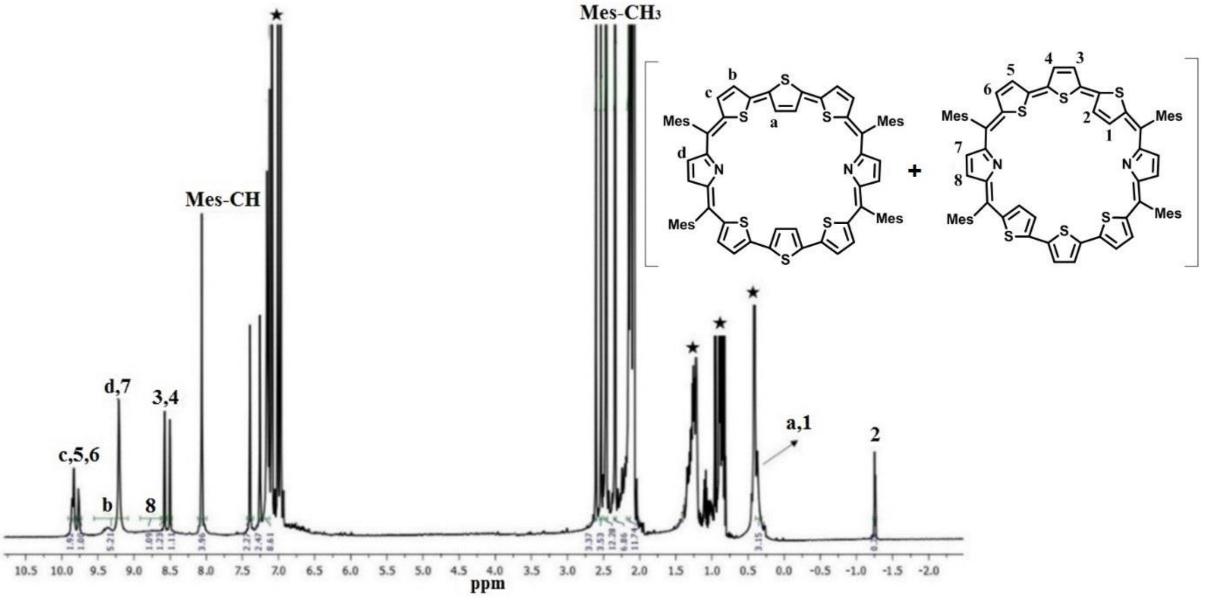

Figure S15: ^{1}H NMR spectra of **10** in Toluene-D_8 at 298K (* residual solvent peaks).

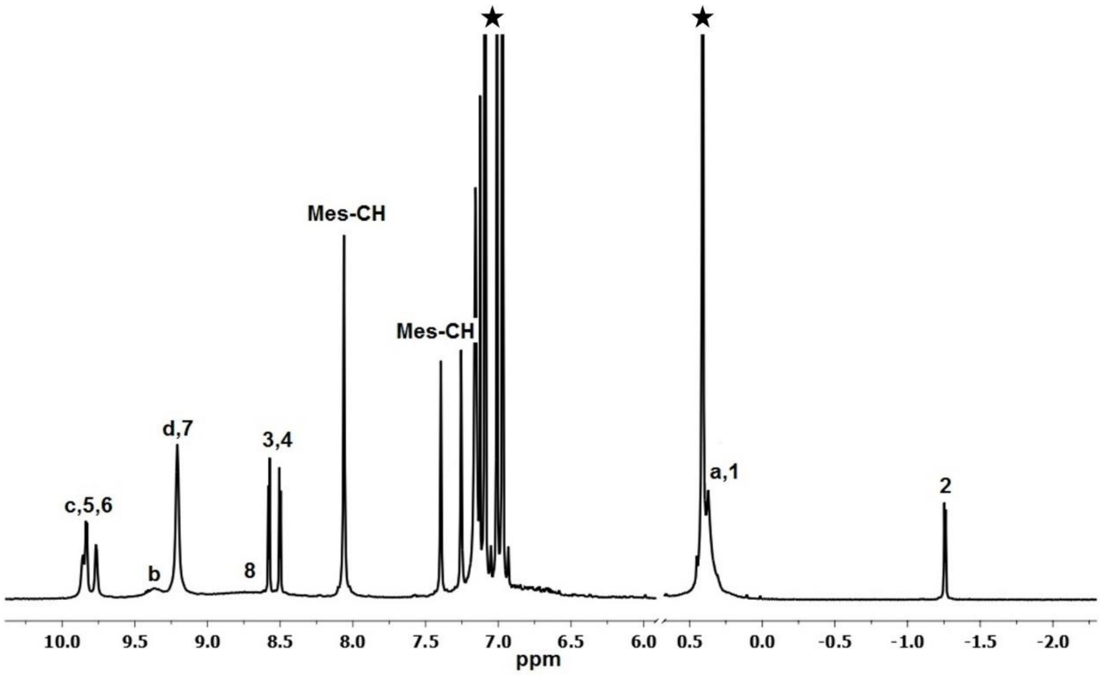

Figure S16: ^{1}H NMR spectra of **10** in Toluene-D_8 at 298K with expansion in aromatic region (* residual solvent peaks).

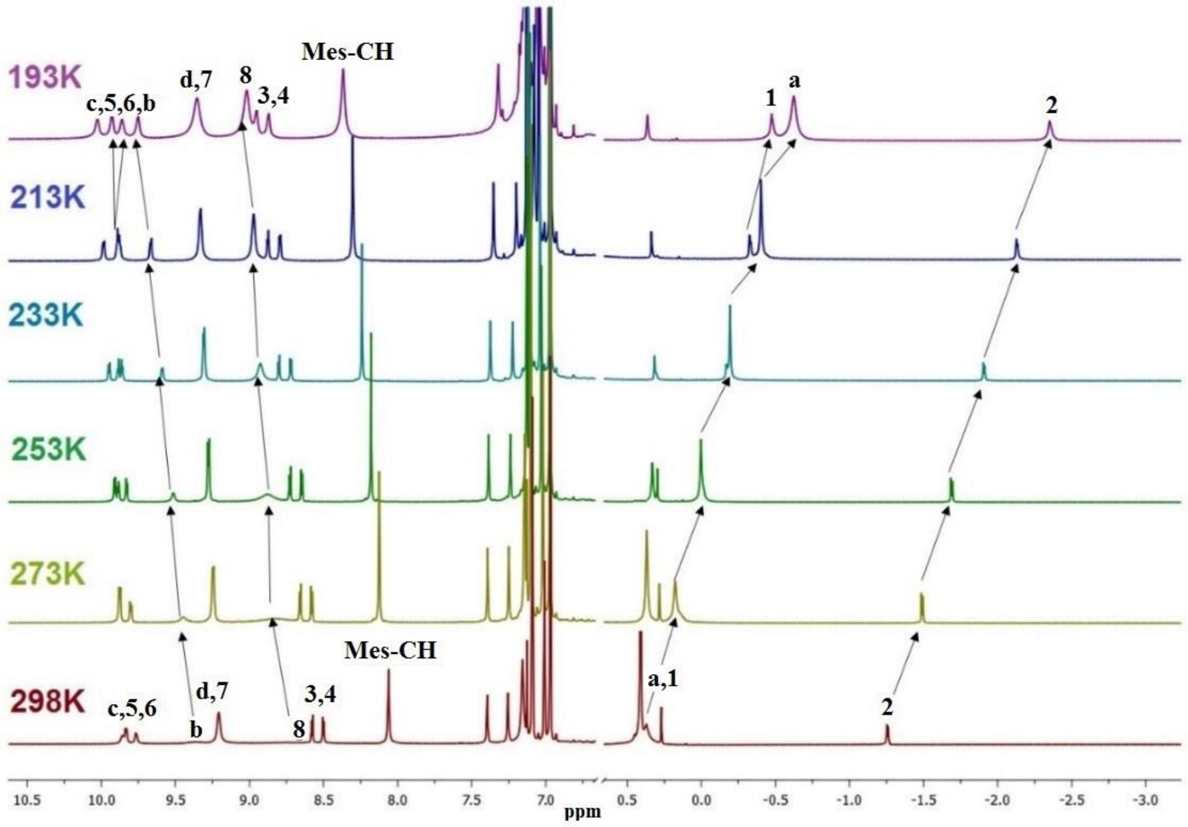

Figure S17: Low temperature (298K-193K) ^{1}H NMR spectrum of **10** in Toluene-D$_8$.

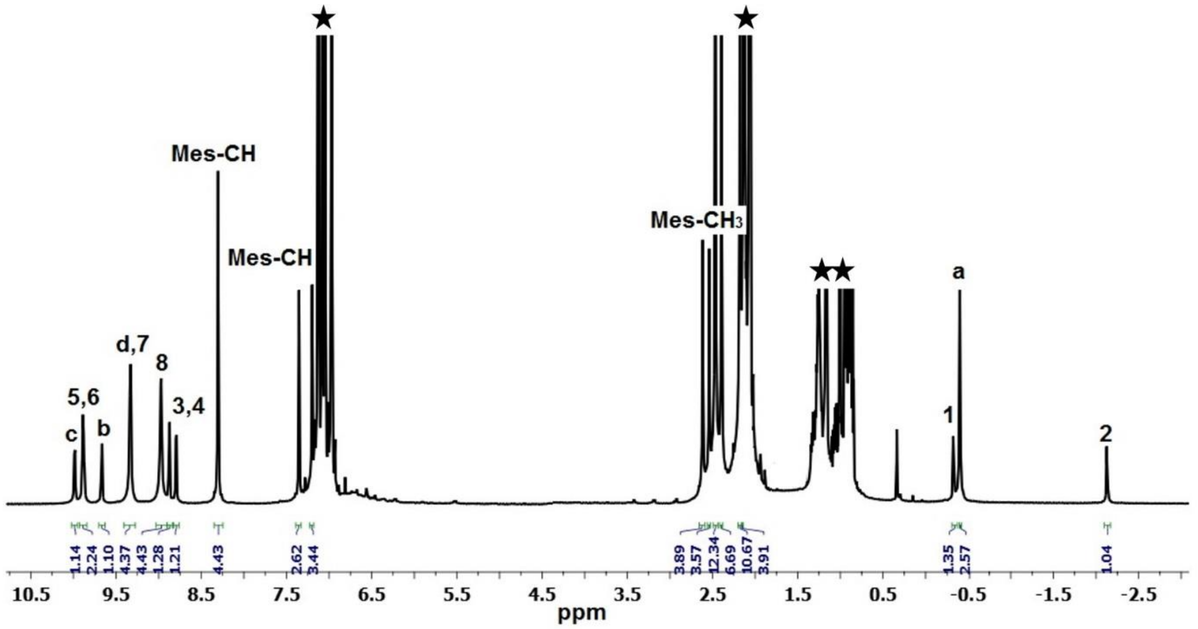

Figure S18: ^{1}H NMR spectrum of **10** in Toluene-D$_8$ at 213K (* residual solvent peaks).

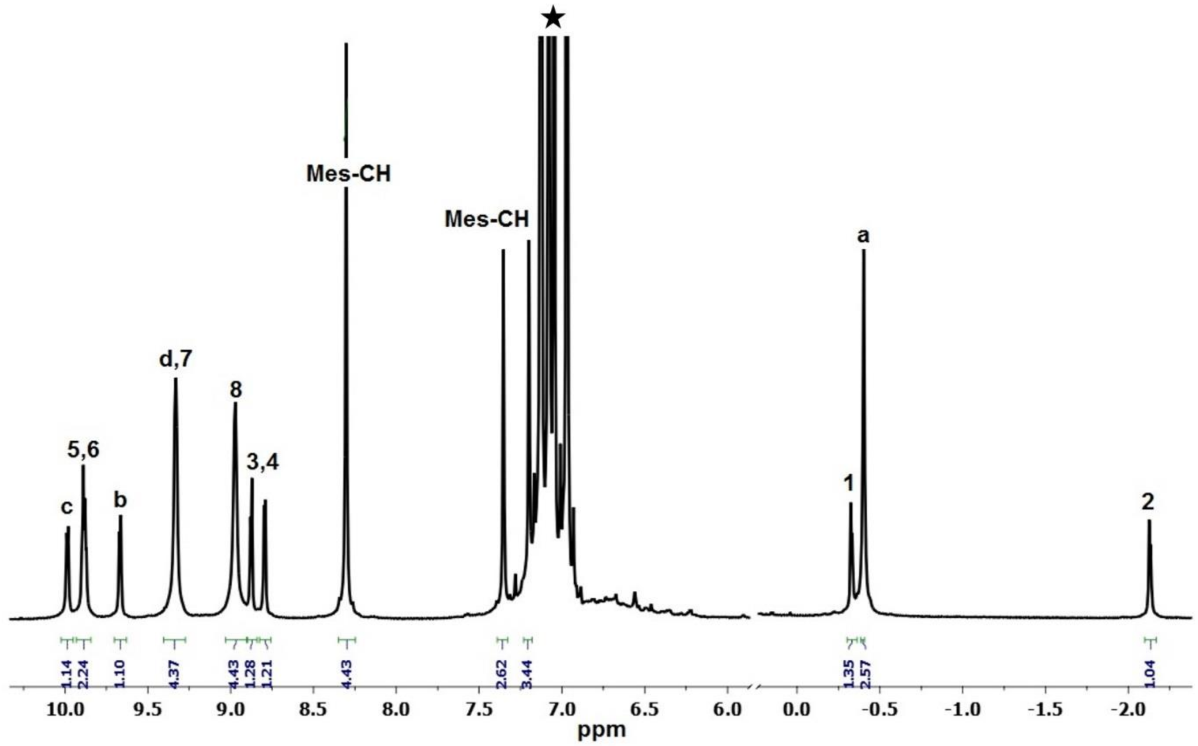

Figure S19: ^{1}H NMR spectrum of **10** in Toluene-D_8 at 213K with expansion in aromatic region (* residual solvent peaks).

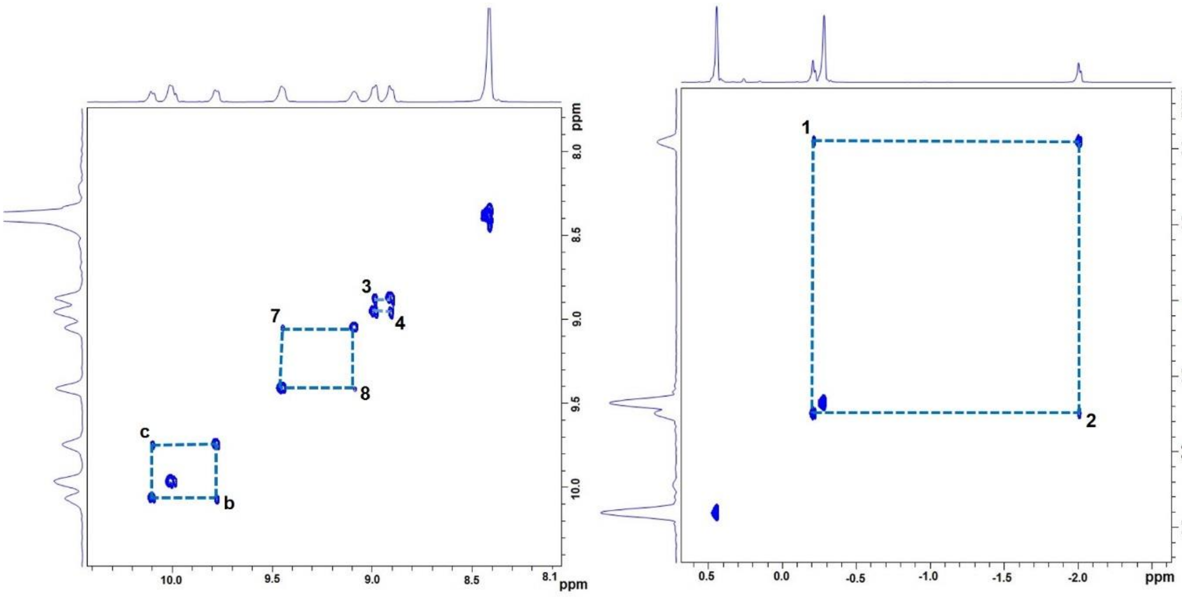

Figure S20: ^{1}H-^{1}H COSY correlation spectrum of **10** at 213K in Toluene-D_8.

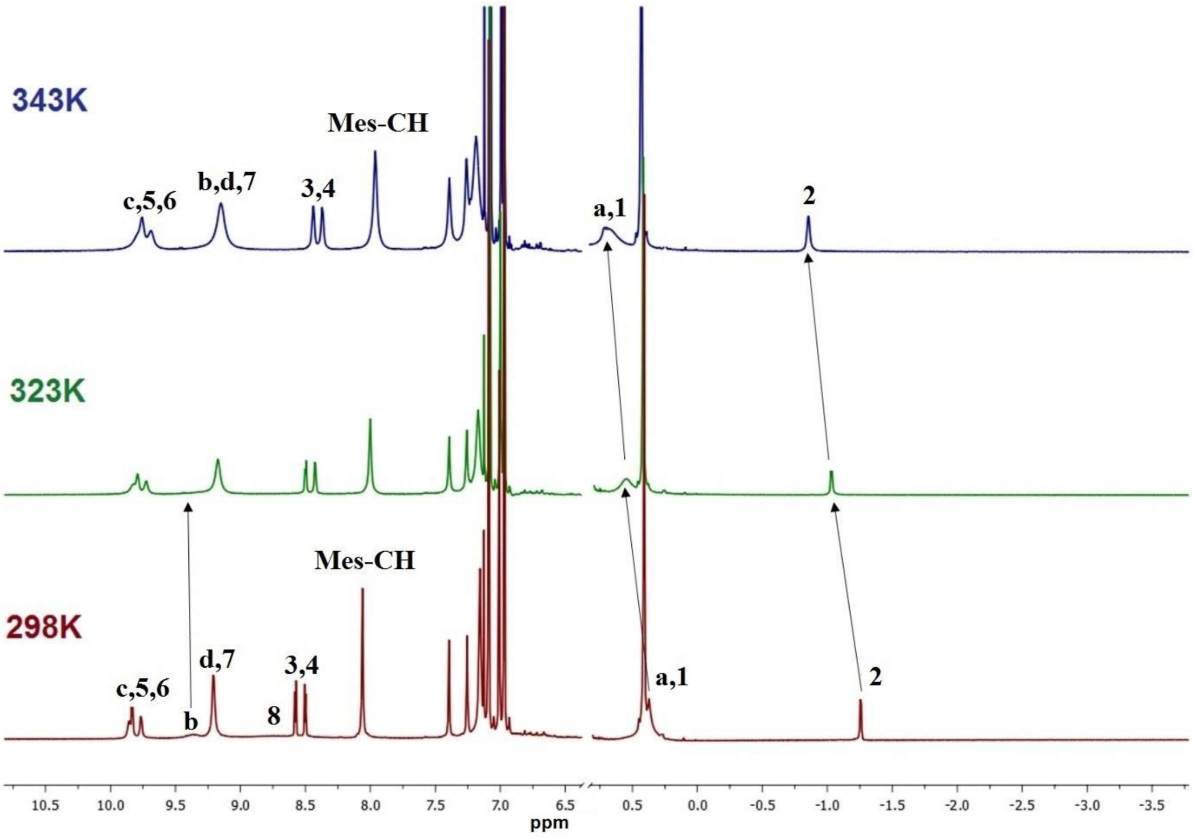

Figure S21: High temperature (298K-343K) ^{1}H NMR spectrum of **10** in Toluene-D$_8$.

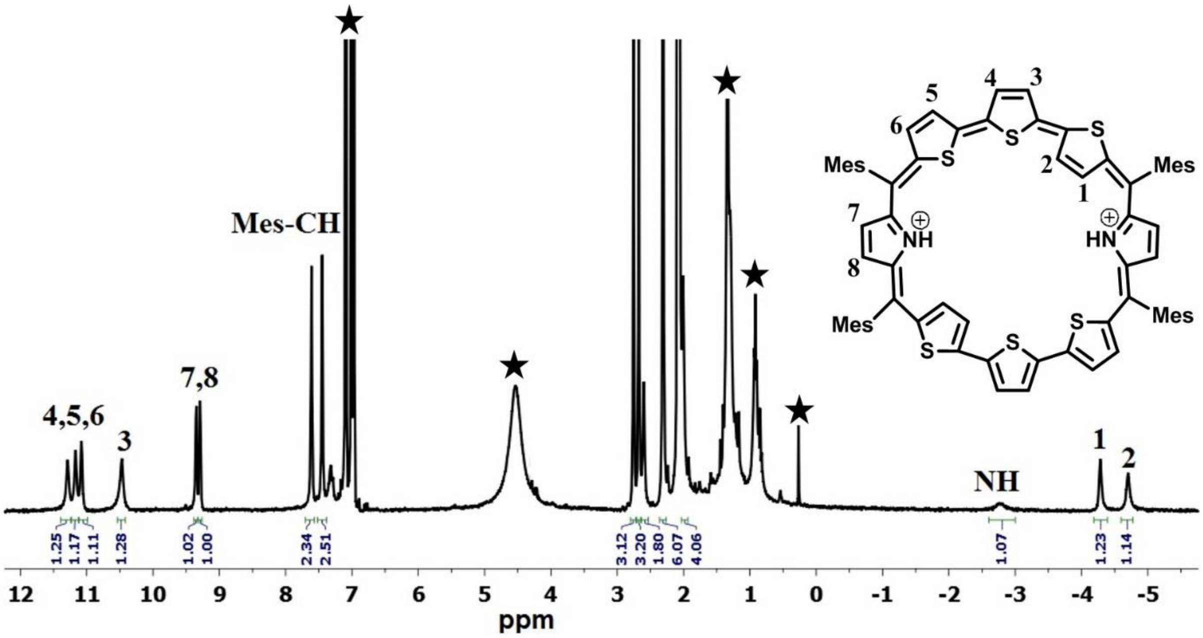

Figure S22: ^{1}H NMR spectrum of **10**.$2H^{+}$ in Toluene-D_8 at 298K (* residual solvent peaks).

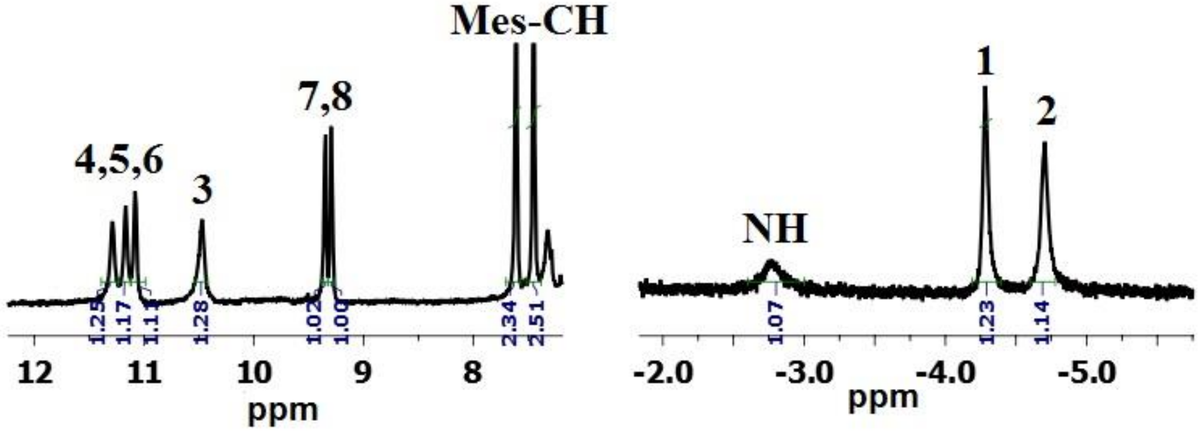

Figure S23: ^{1}H NMR spectra of **10**. $2H^{+}$ in Toluene-D_8 at 298K with expansion in Aromatic region (* residual solvent peaks).

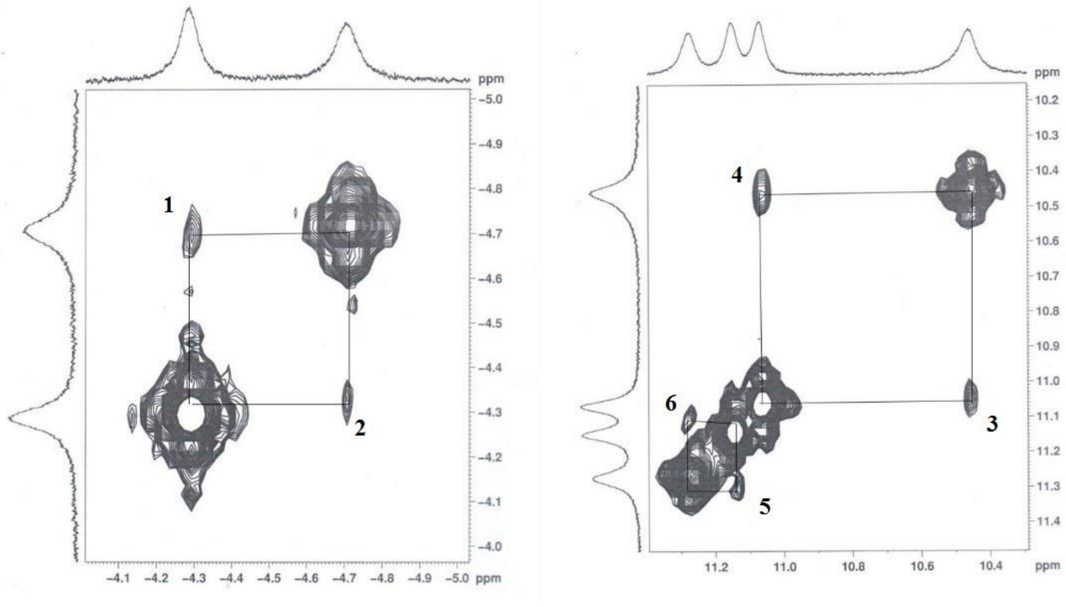

Figure S24: ^{1}H-1H COSY correlation spectrum of **10**.2H$^+$ at 298K in Toluene-D$_8$.

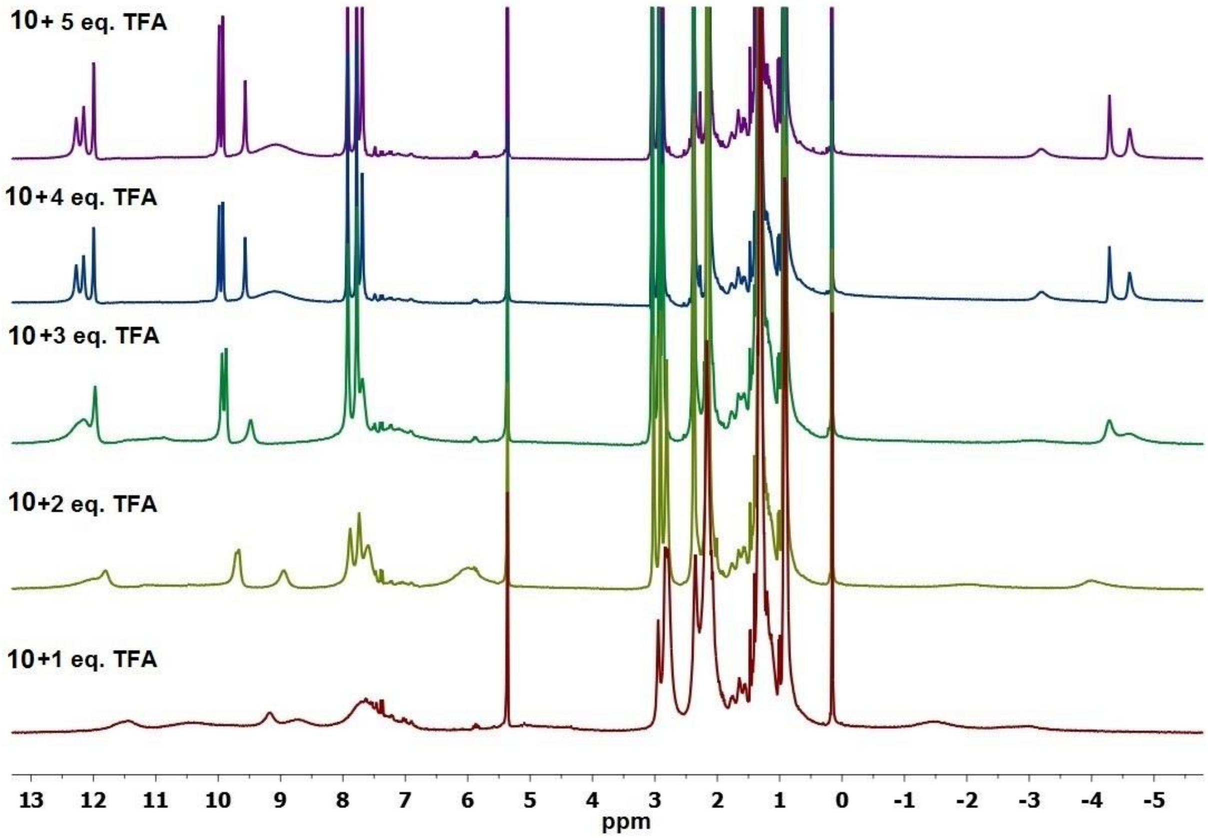

Figure S25: ^{1}H NMR spectra of **10** with varying concentration of TFA in CH_2Cl_2 at 298K.

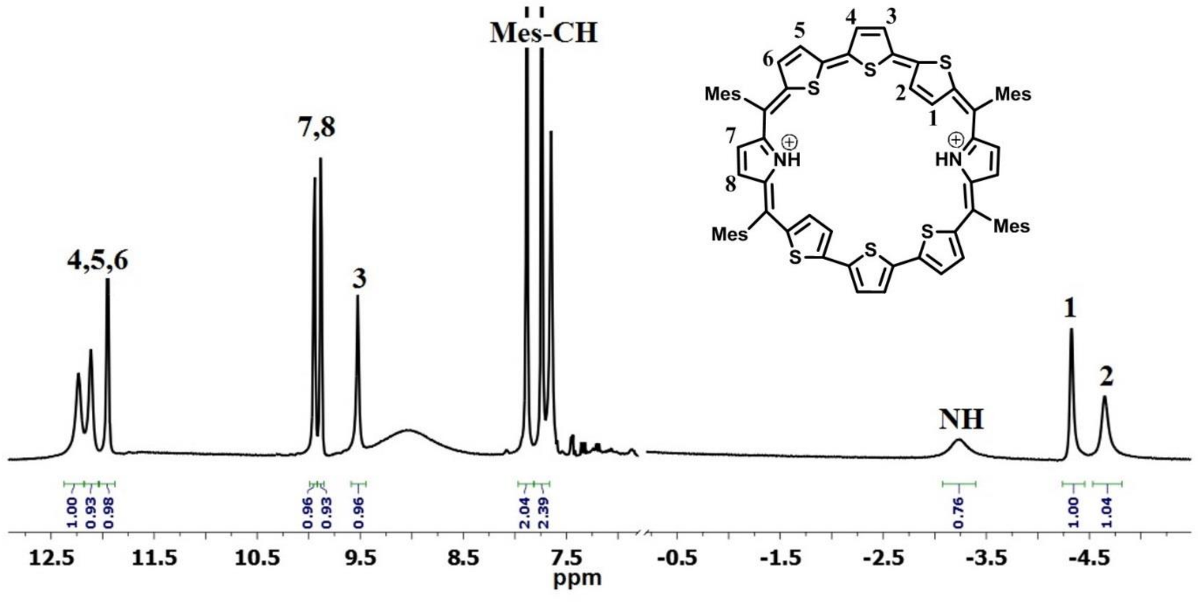

Figure S26: ^{1}H NMR spectrum of **10**.2H$^+$ in CH_2Cl_2 at 298K with expansion of Aromatic region.

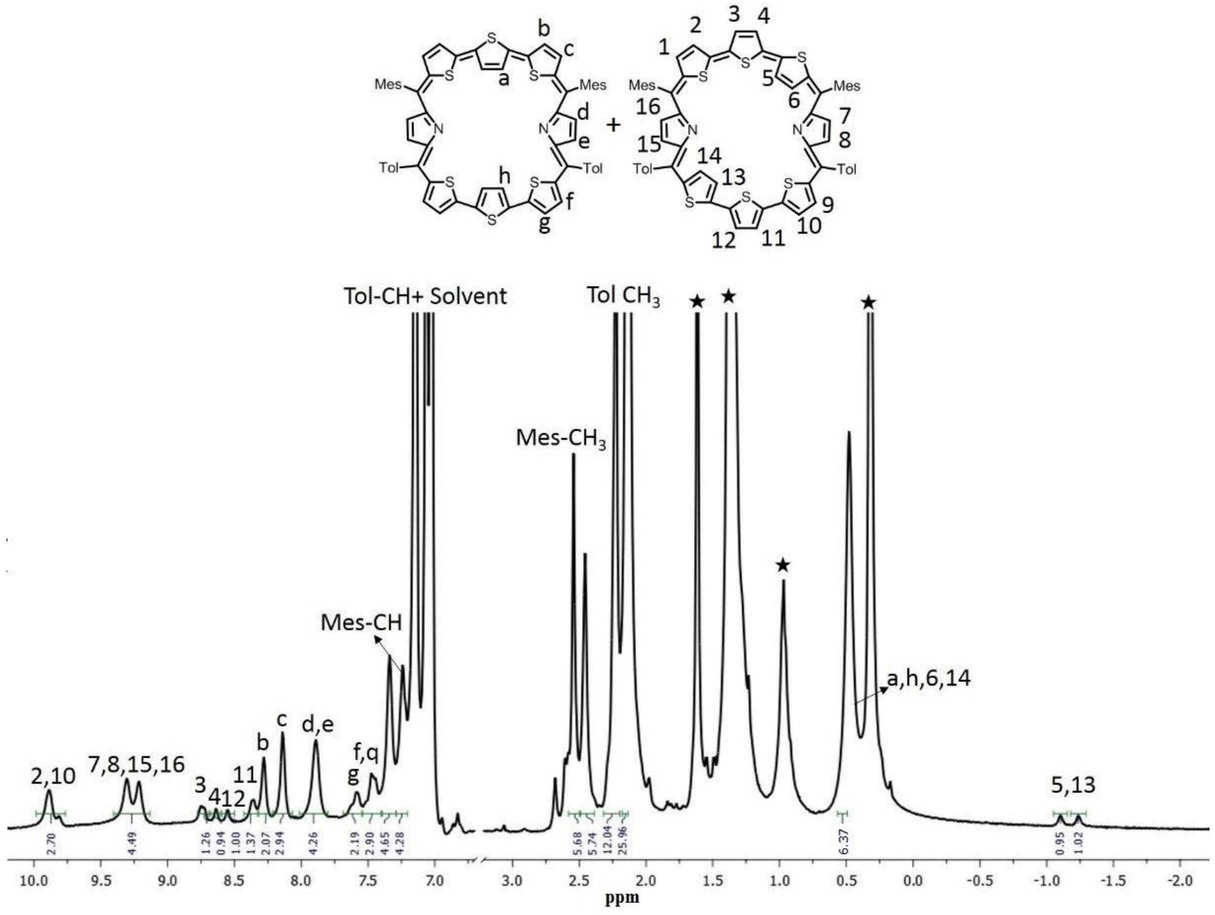

Figure S27: ^{1}H NMR spectrum of **11** in Toluene-D$_8$ at 298K (* residual solvent peaks).

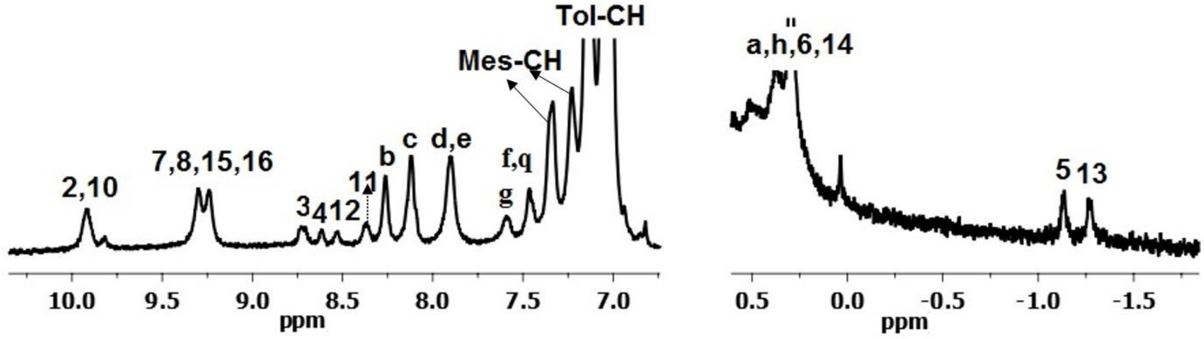

Figure S28: ^{1}H NMR spectrum of **11** in Toluene-D$_8$ at 298K with expansion in Aromatic region.

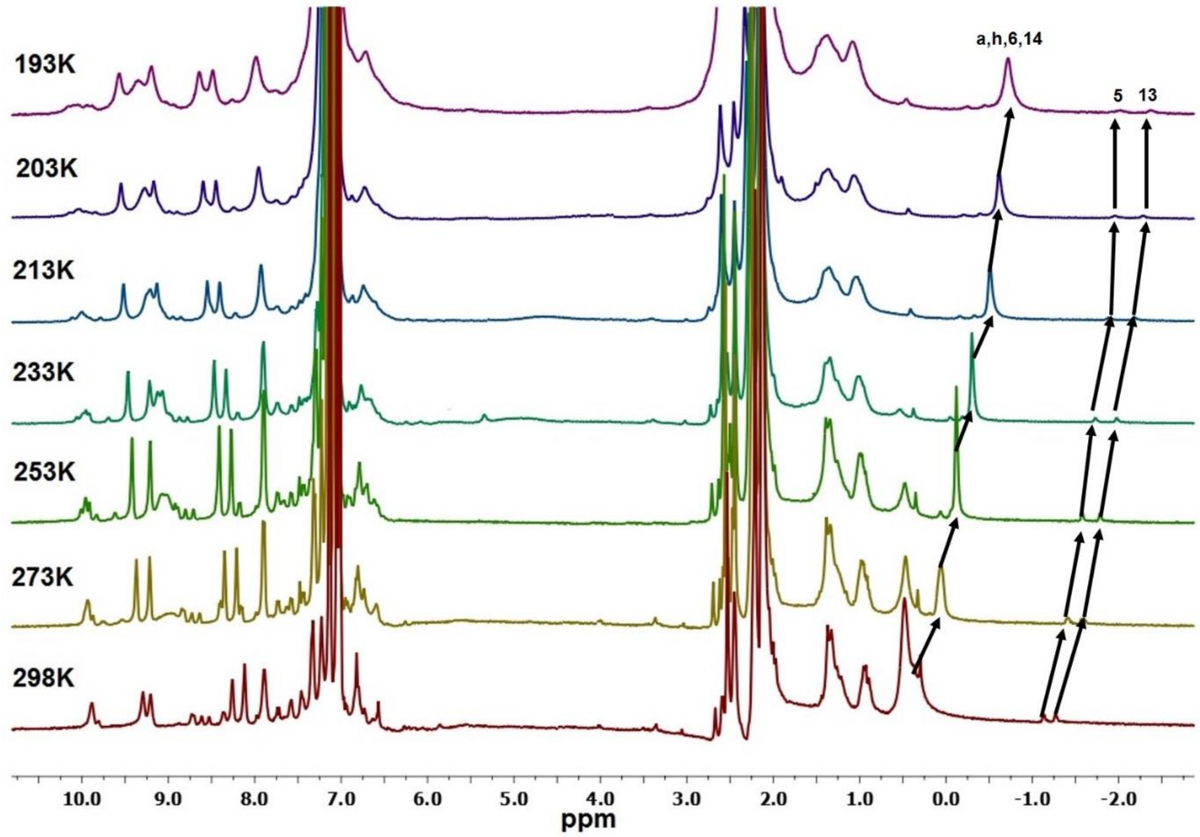

Figure S29: Low temperature (298K-193K) ^{1}H NMR spectrum of **11** in Toluene-D_8.

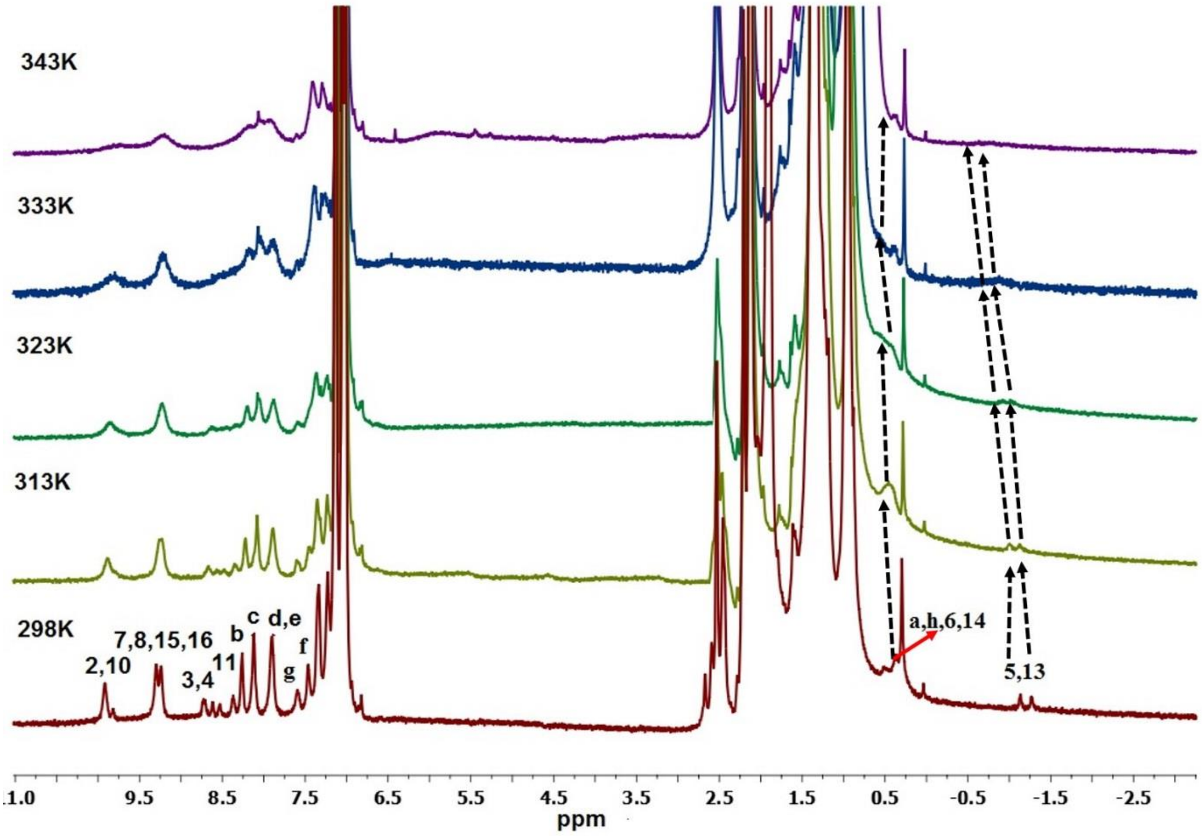

Figure S30: High temperature (298K-343K) ^{1}H NMR spectrum of **11** in Toluene-D_8.

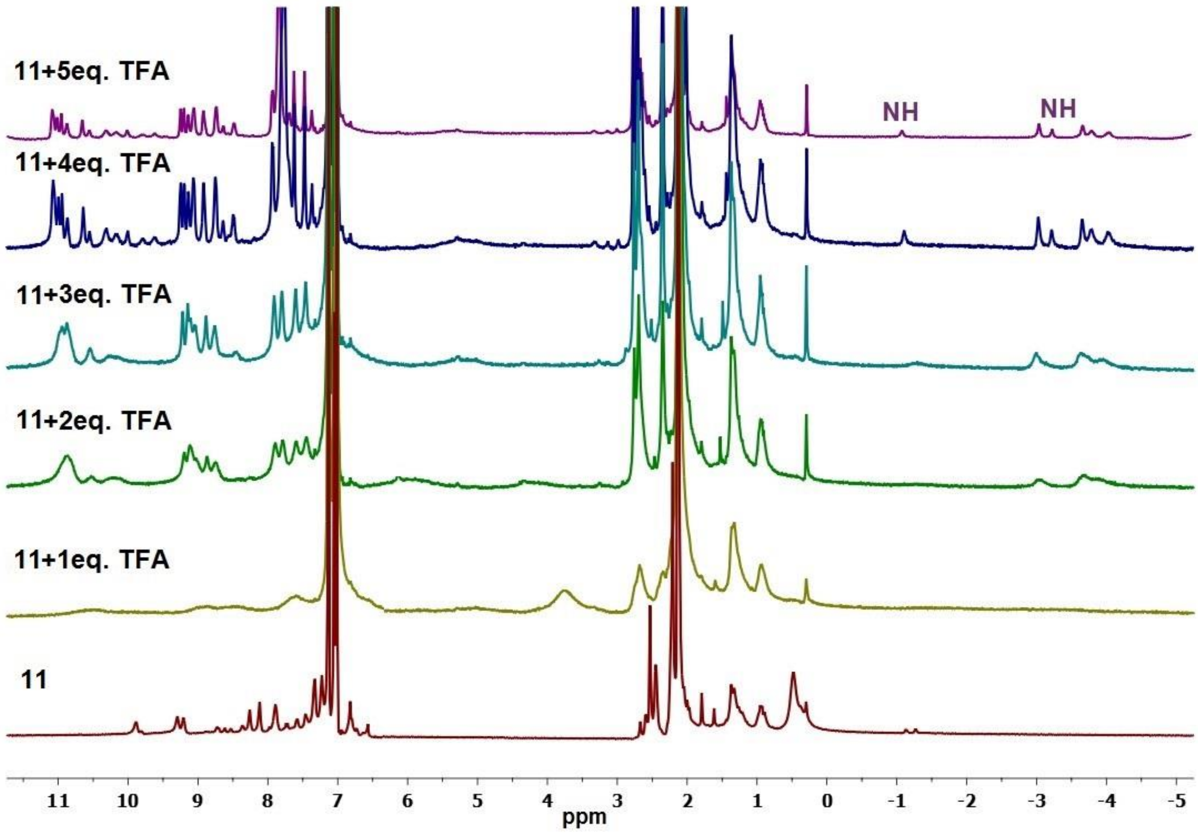

Figure S31: ^{1}H NMR spectrum of **11** in Toluene-D_8 at 298K with varying concentration of TFA.

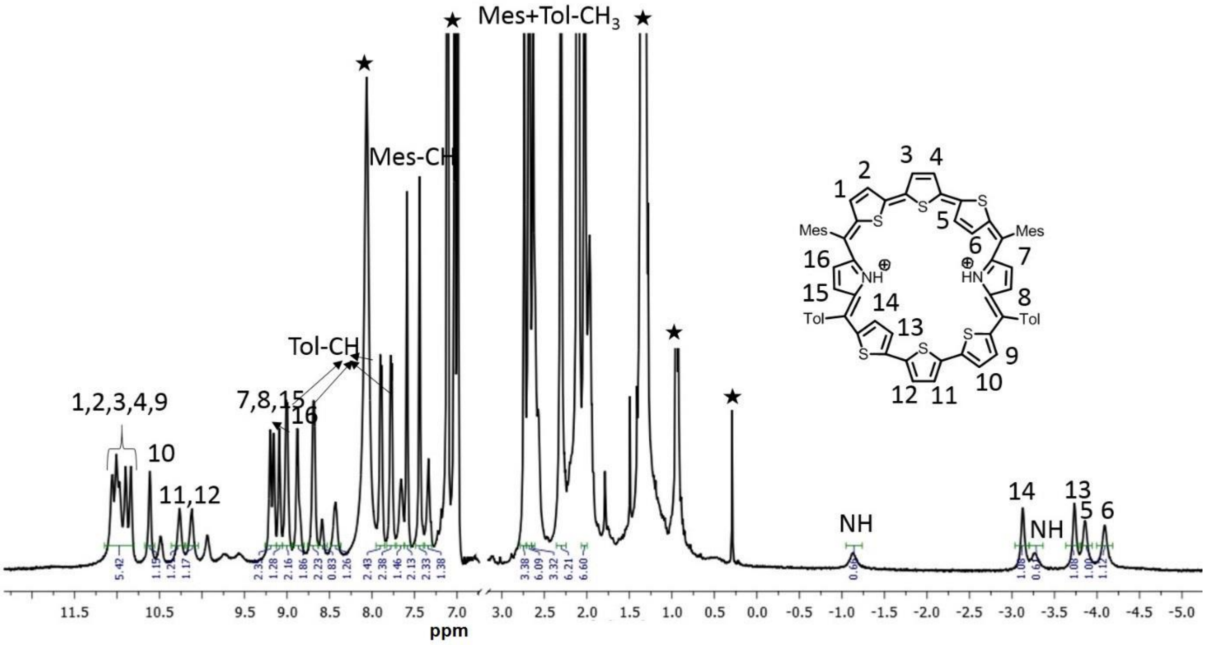

Figure S32: ^{1}H NMR spectrum of **11**.2H^{+} in Toluene-D$_8$ at 298K (* residual solvent peaks).

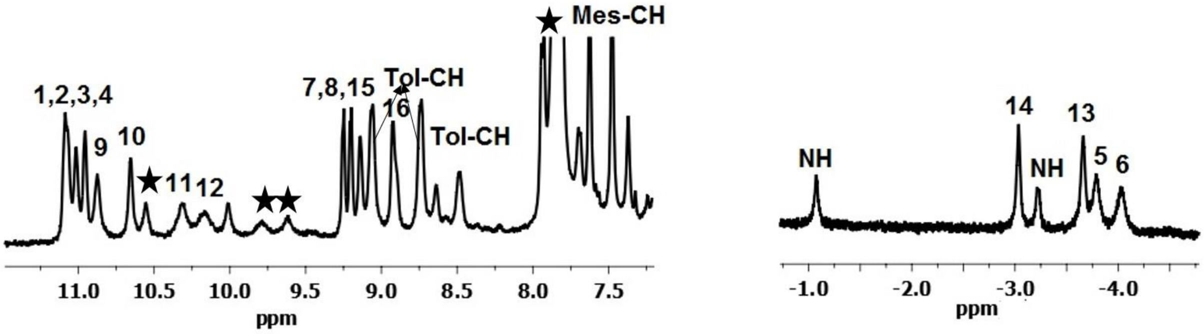

Figure S33: ^{1}H NMR spectrum of **11**.2H^{+} in Toluene-D$_8$ at 298K with expansion in the Aromatic region (* residual solvent peaks).

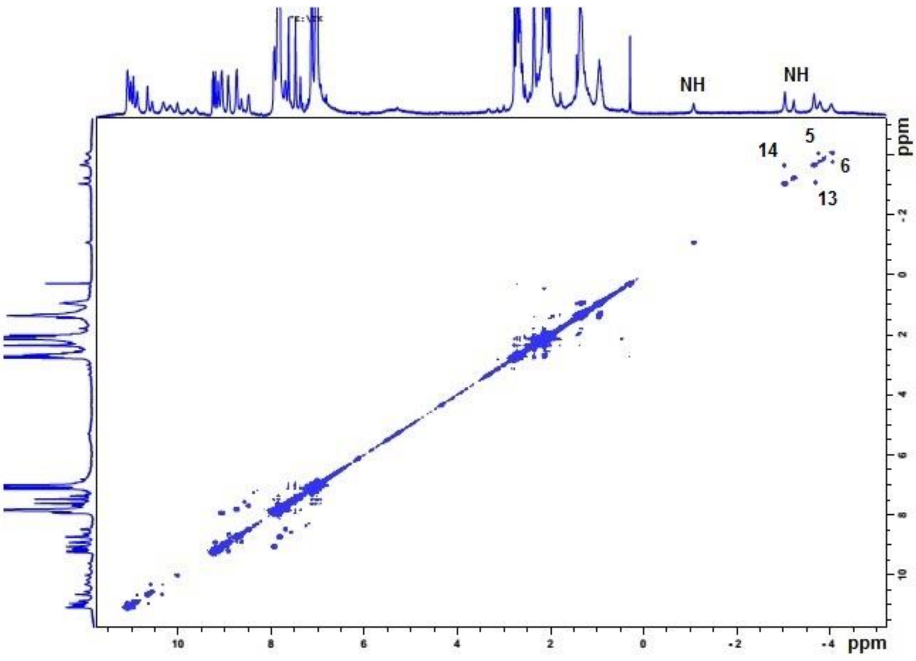

Figure S34: ^1H-^1H COSY spectrum of **11**.$2H^+$ at 298K in Toluene-D_8.

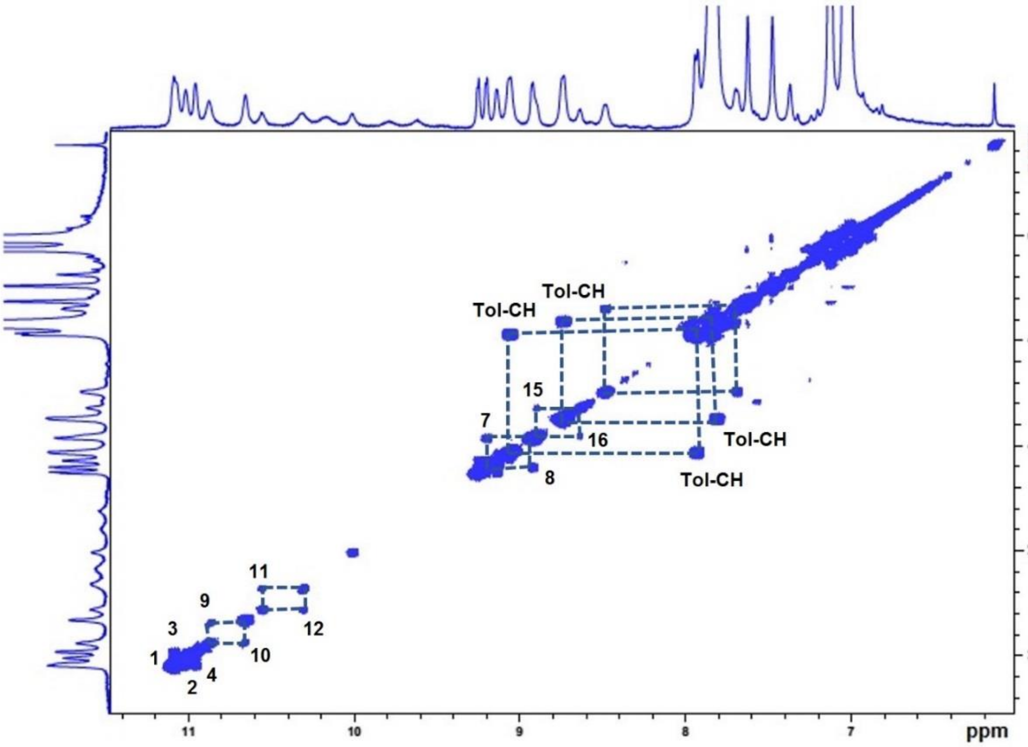

Figure S35: ^1H-^1H COSY spectrum of **11**.$2H^+$ at 298K in Toluene-D_8 with expansion in the Aromatic region.

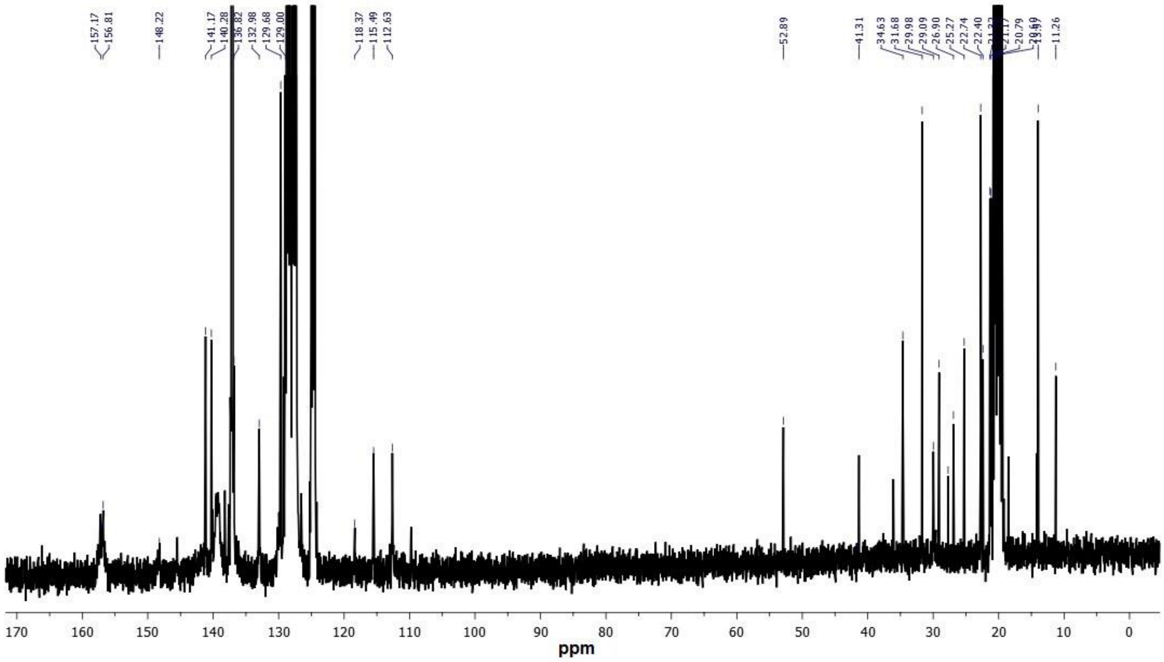

Figure S36: ^{13}C NMR spectrum of **11**.$2H^{+}$ in Toluene-D_8 at 298K.

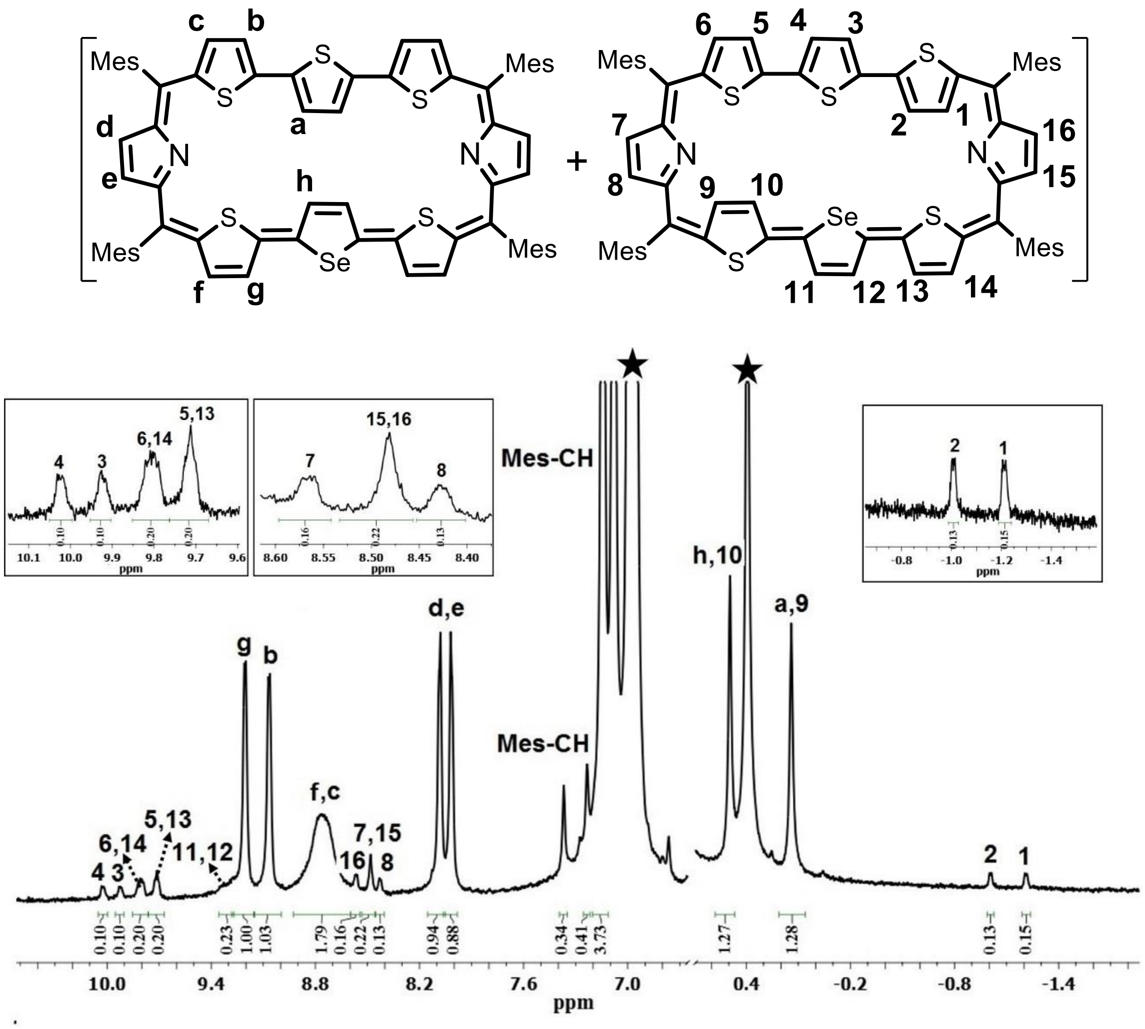

Figure S37: ^{1}H NMR spectrum of **13** in Toluene-D$_8$ at 298K.

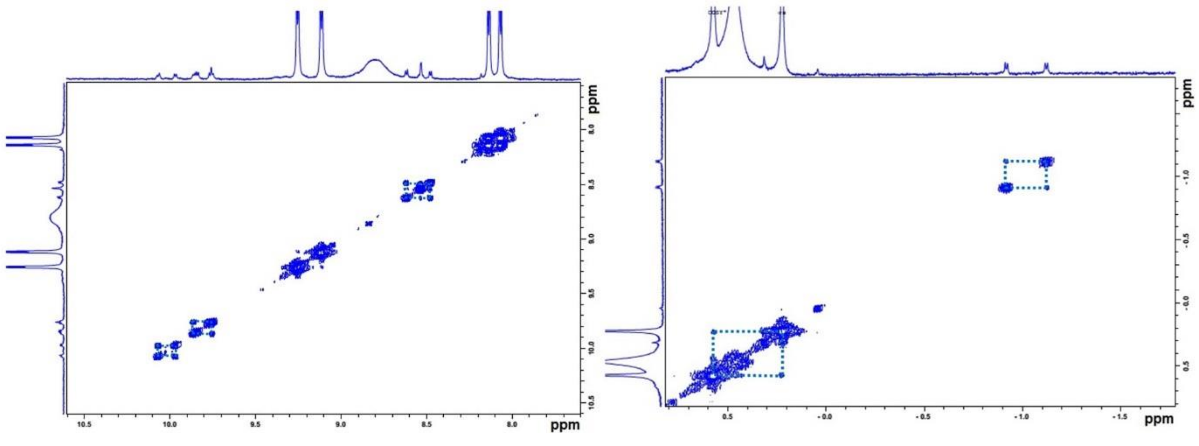

Figure S38: ^{1}H-^{1}H COSY spectrum of **13** at 298K in Toluene-D$_8$.

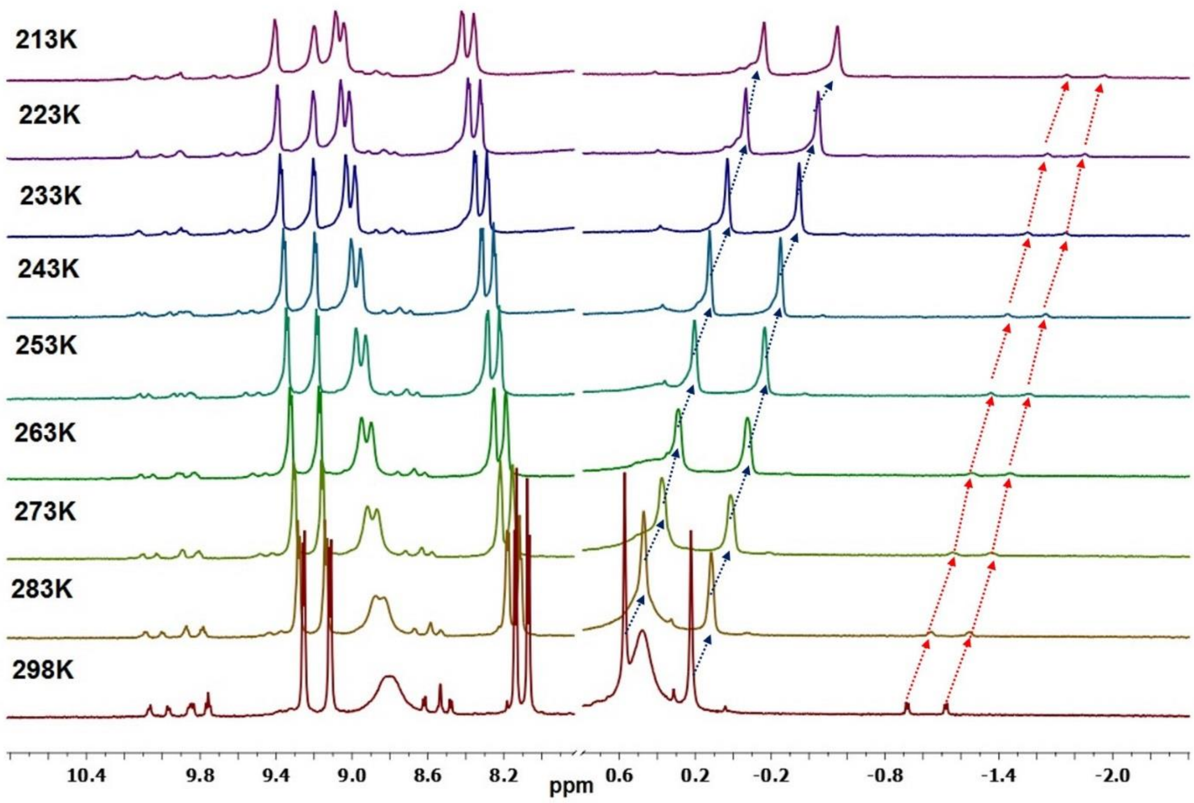

Figure S39: Low temperature (298K-193K) ^{1}H NMR spectrum of **13** in Toluene-D_8.

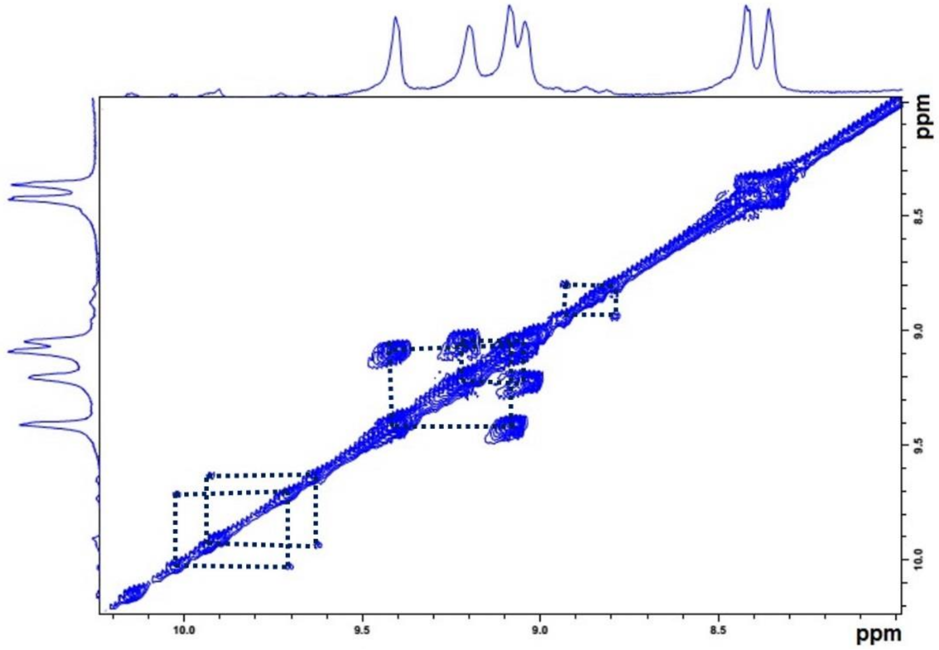

Figure S40: ^{1}H-^{1}H COSY spectrum of **13** at 213K in Toluene-D_8.

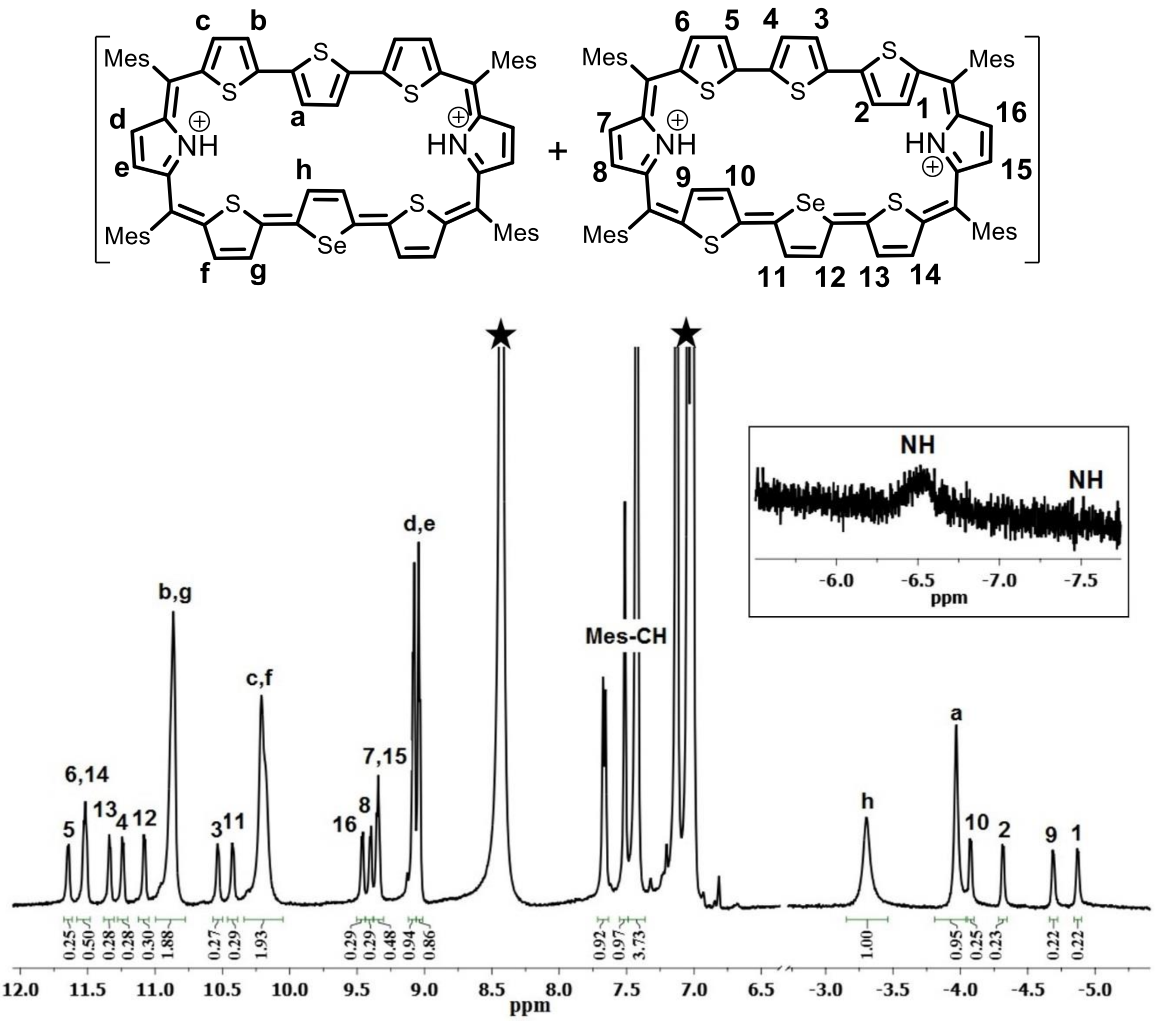

Figure S41: ^{1}H NMR spectrum of **13**.2H^{+} in Toluene-D$_8$ at 298K (* residual solvent peaks).

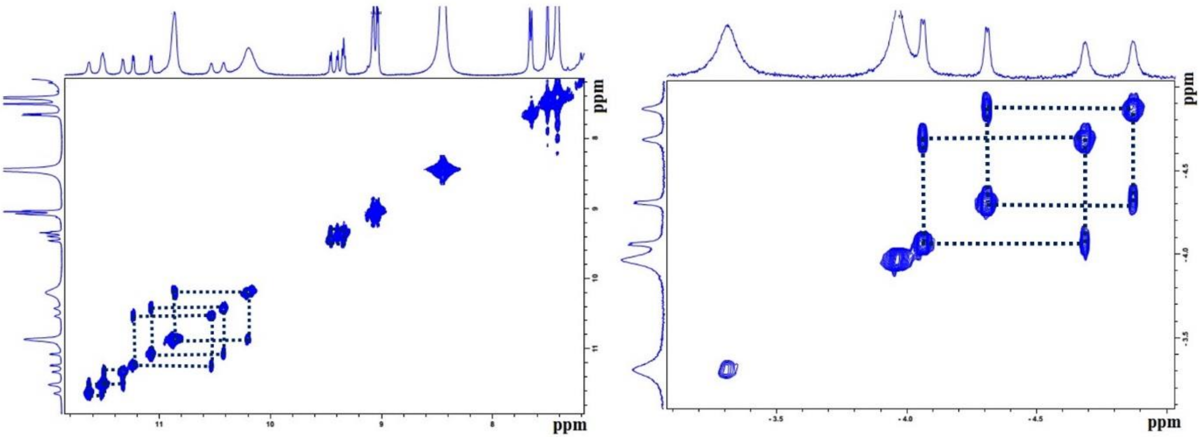

Figure S42: ^{1}H-^{1}H COSY spectrum of **13**.2H^{+} at 213K in Toluene-D$_8$.

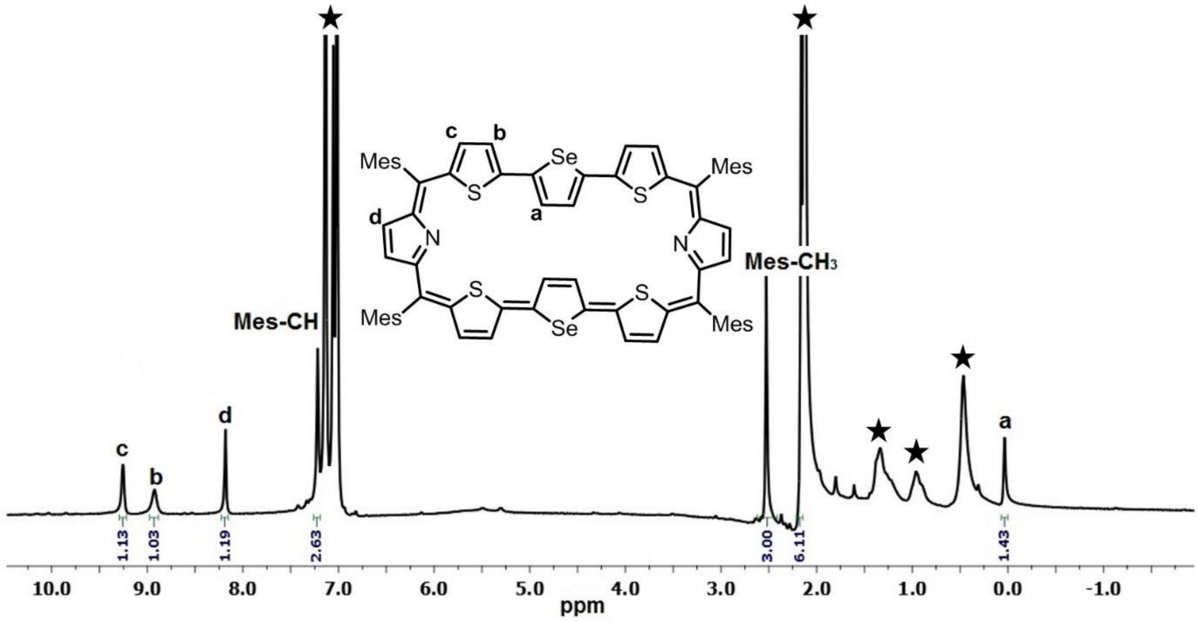

Figure S43: ^{1}H NMR spectrum of **15A** in Toluene-D$_8$ at 298K

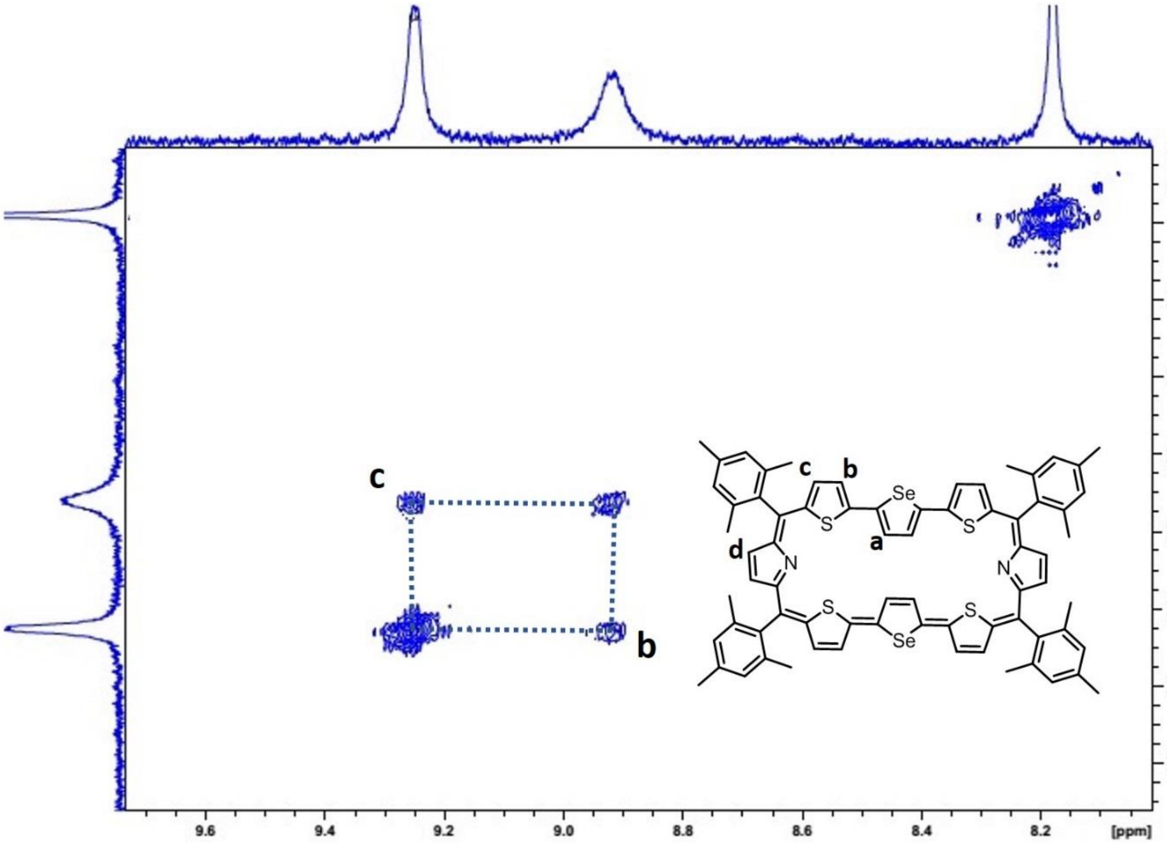

Figure S44: ^{1}H-^{1}H COSY spectrum of **15A** at 213K in Toluene-D$_8$.

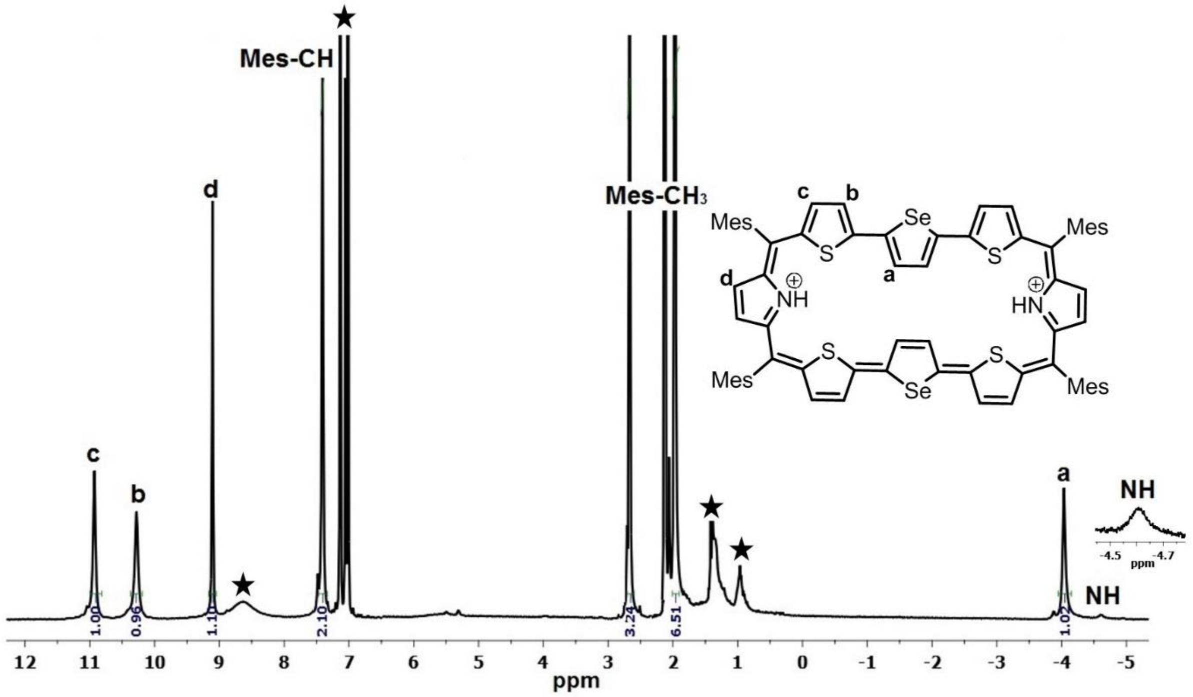

Figure S45: ^{1}H NMR spectrum of **15A**.2H$^+$ in Toluene-D$_8$ at 298K (* residual solvent peaks).

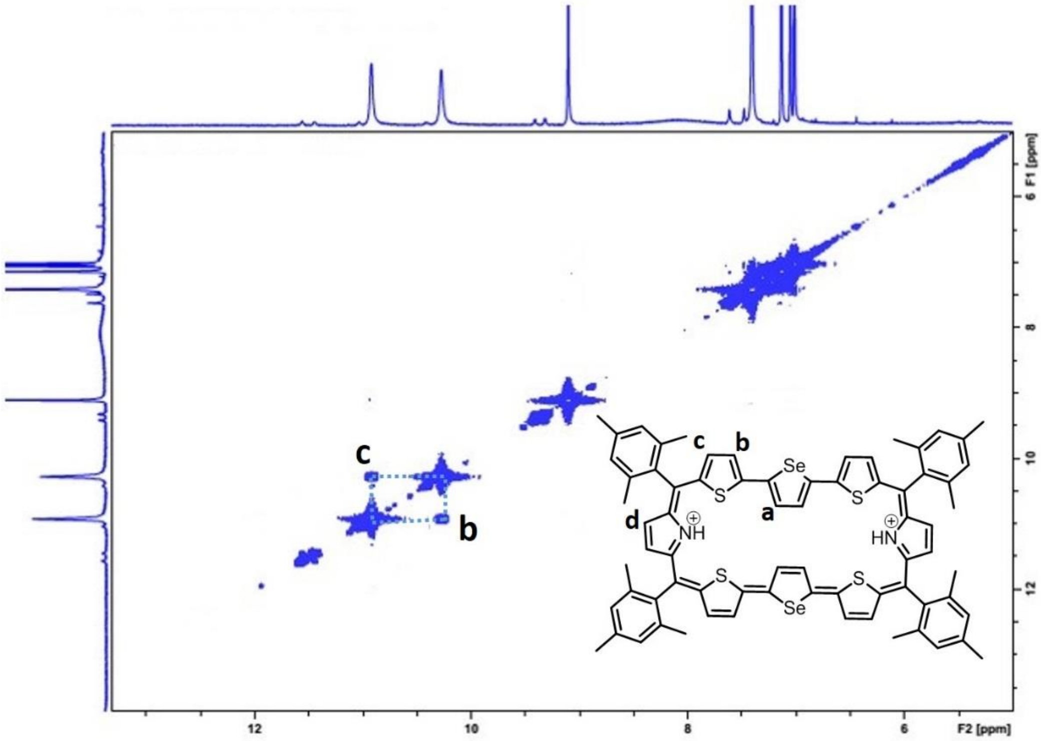

Figure S46: ^{1}H-^{1}H COSY spectrum of **15A**.2H$^+$ at 213K in Toluene-D$_8$.

<table>
<tr><th>10A</th><th>10B</th></tr>
<tr><td>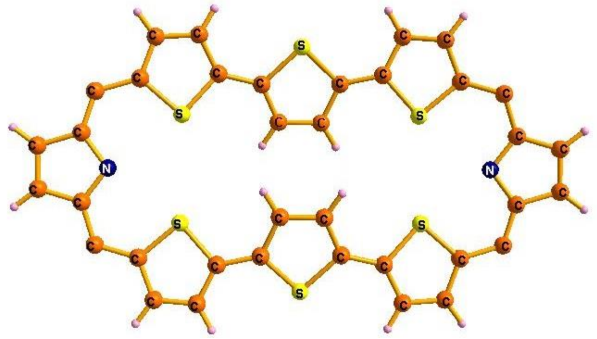
X-ray structure</td><td>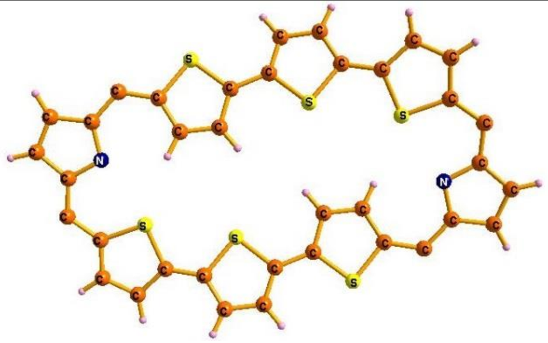
X-ray structure</td></tr>
<tr><td>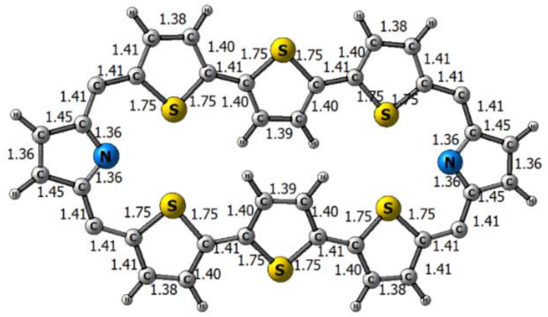
M06L/6-31G** level optimized geometry</td><td>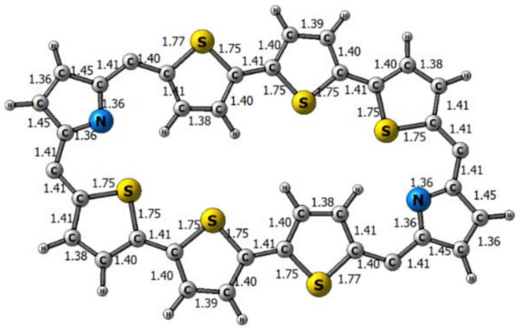
M06L/6-31G** level optimized geometry</td></tr>
<tr><td>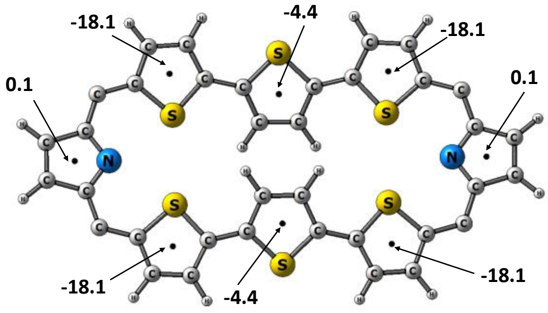
NICS(0) values at M06L/6-31G** level</td><td>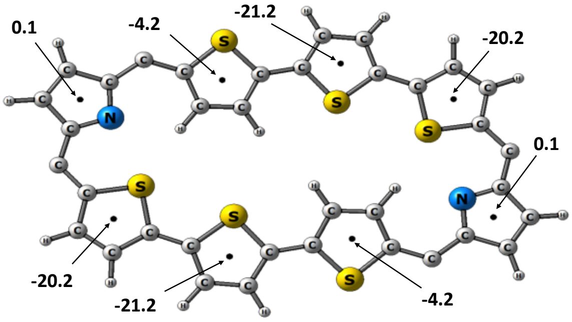
NICS(0) values at M06L/6-31G** level</td></tr>
<tr><td>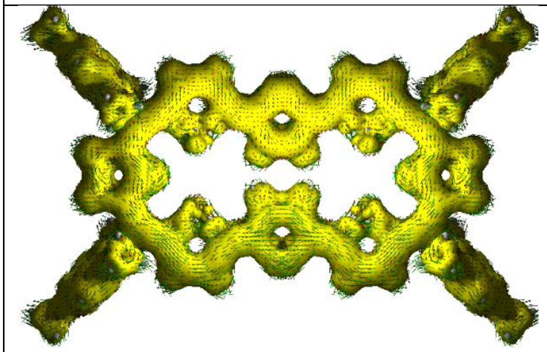
AICD Plot - the current density vectors plotted on to isosurface of value 0.026.</td><td>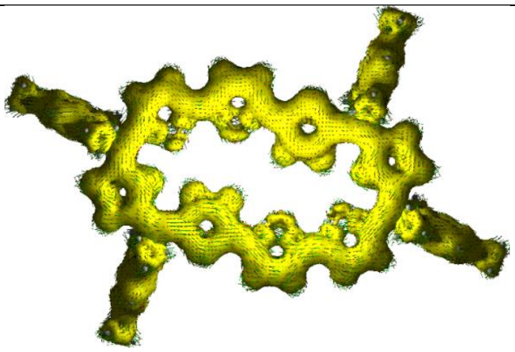
AICD Plot - the current density vectors plotted on to isosurface of value 0.026.</td></tr>
</table>

Chart S1a: X-ray structure, Optimized geometry, NICS (0) values and AICD plots for **10A** and **10B**

10B.2H$^+$:

10B.2H$^+$	10B.2H$^+$
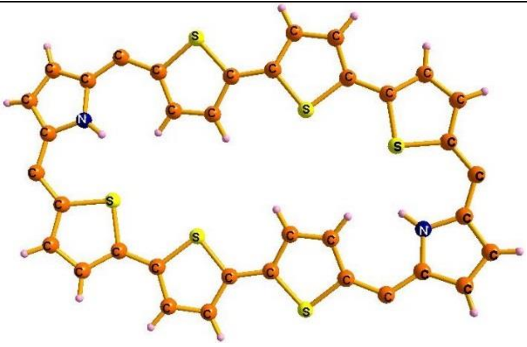 X-ray structure	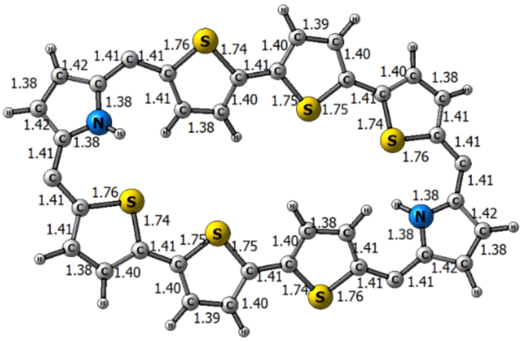 M06L/6-31G** level optimized geometry
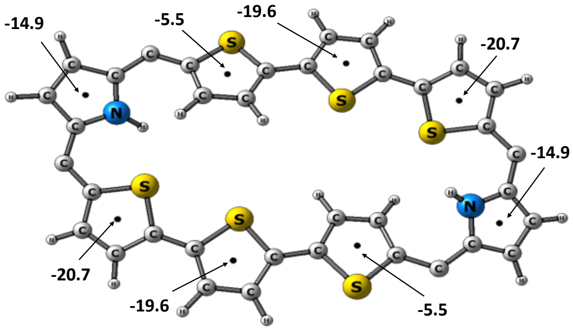 **NICS(0) values at M06L/6-31G** level**	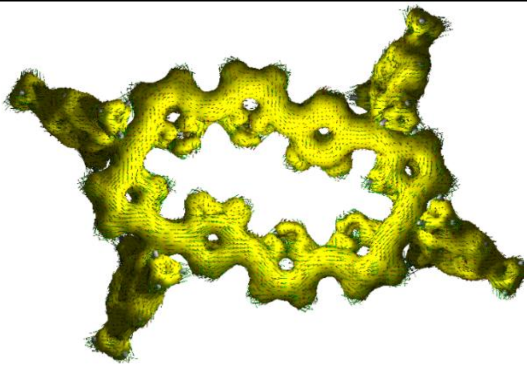 **AICD Plot - the current density vectors plotted on to isosurface of value 0.026.**

Chart S1b: X-ray structure, Optimized geometry, NICS (0) values and AICD plots for **10B**.2H$^+$

<table>
<tr><th>13A</th><th>13B</th></tr>
<tr><td>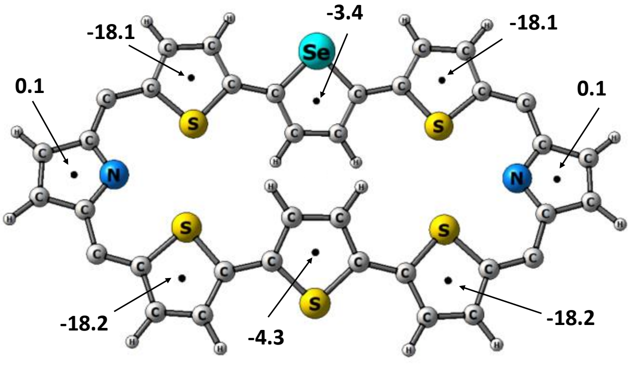

NICS(0) values at M06L/6-31G** level</td><td>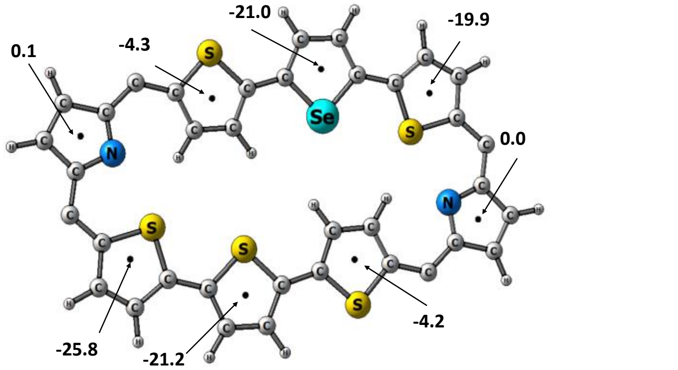

NICS(0) values at M06L/6-31G** level</td></tr>
<tr><td>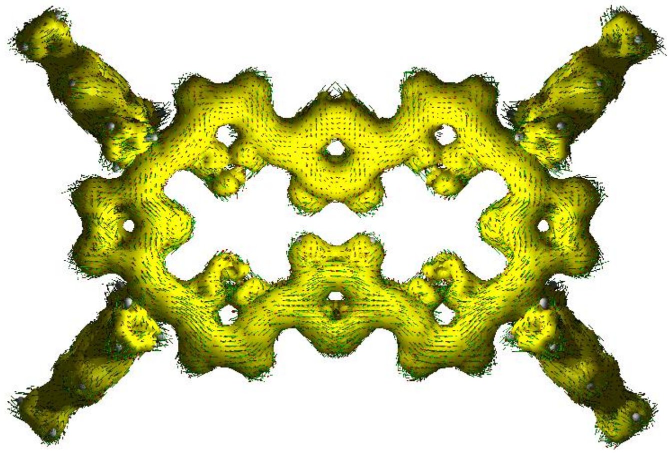
AICD Plot - the current density vectors plotted on to isosurface of value 0.026.</td><td>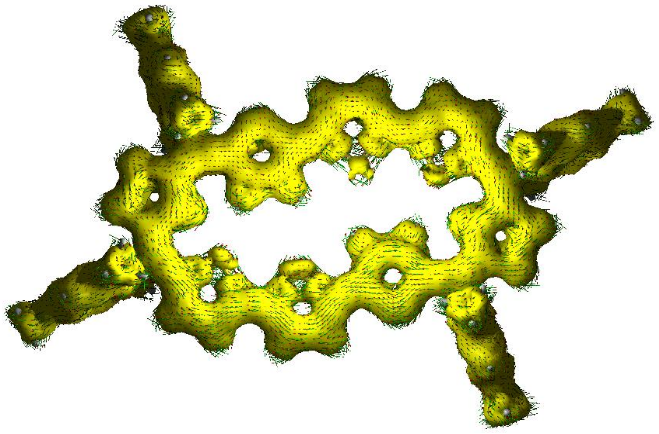
AICD Plot - the current density vectors plotted on to isosurface of value 0.026.</td></tr>
</table>

Chart S2a: X-ray structure, Optimized geometry, NICS (0) values and AICD plots for **13A**, **13B**

13A.2H$^+$	13B.2H$^+$
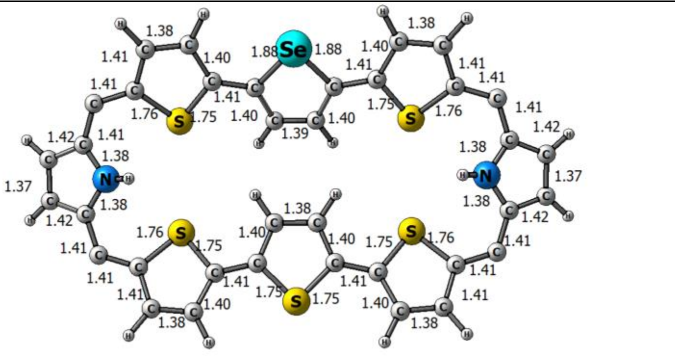 M06L/6-31G** level optimized geometry	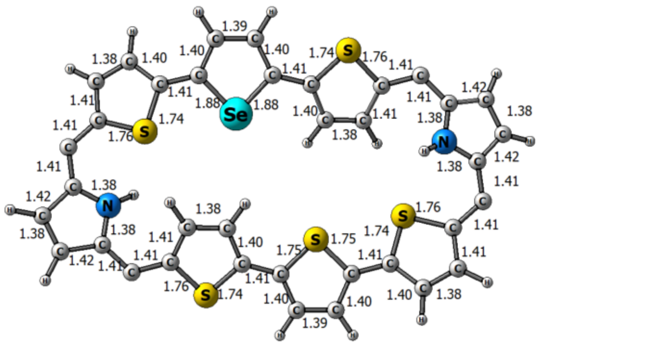 M06L/6-31G** level optimized geometry
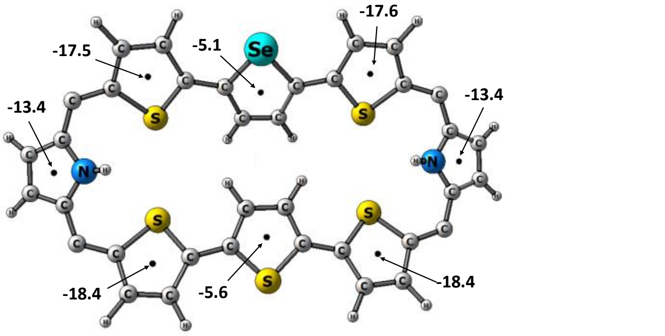 **NICS(0) values at M06L/6-31G** level**	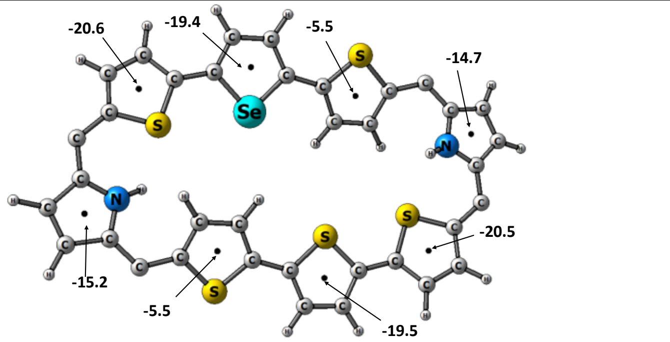 **NICS(0) values at M06L/6-31G** level**
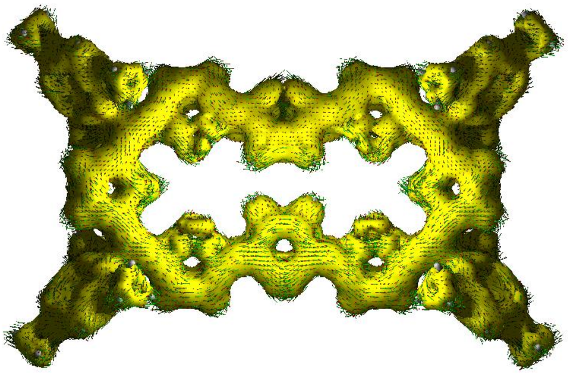 **AICD Plot - the current density vectors plotted on to isosurface of value 0.026.**	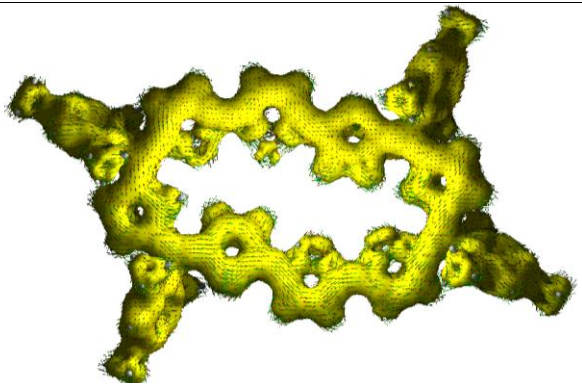 **AICD Plot - the current density vectors plotted on to isosurface of value 0.026.**

Chart S2b: X-ray structure, Optimized geometry, NICS (0) values and AICD plots for **13A.2H$^+$** and **13B**.2H$^+$

**15A**	**15A.2H^{+}**
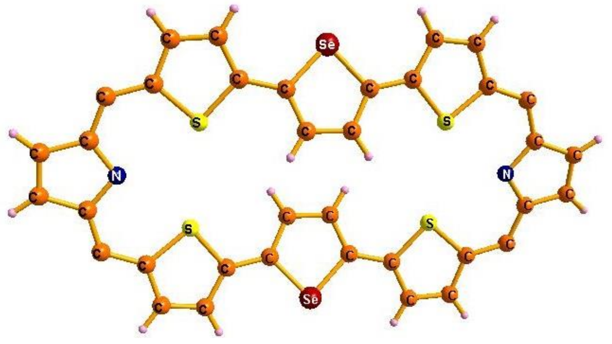<br>X-ray structure	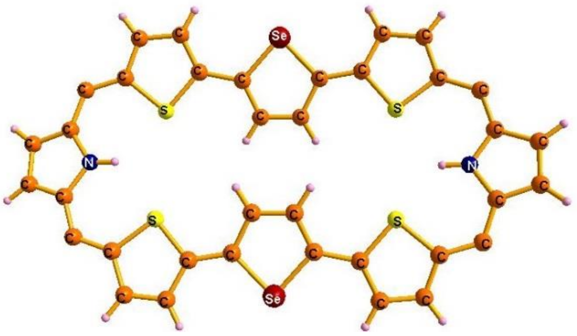<br>X-ray structure
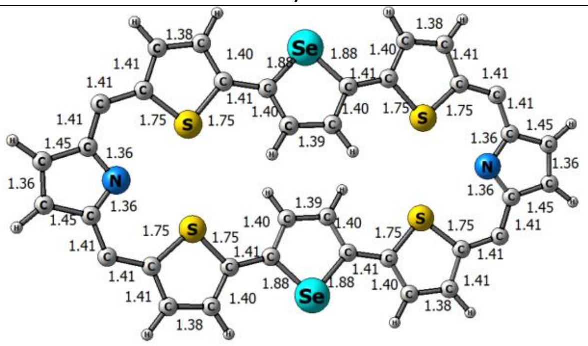<br>M06L/6-31G** level optimized geometry	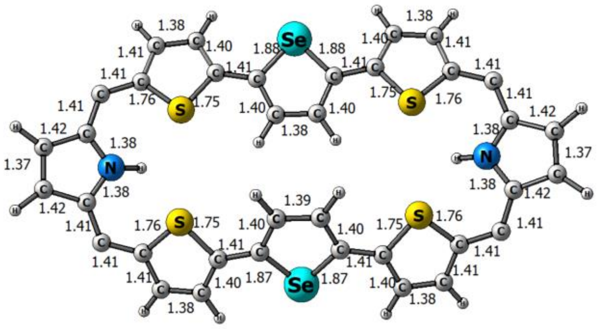<br>M06L/6-31G** level optimized geometry
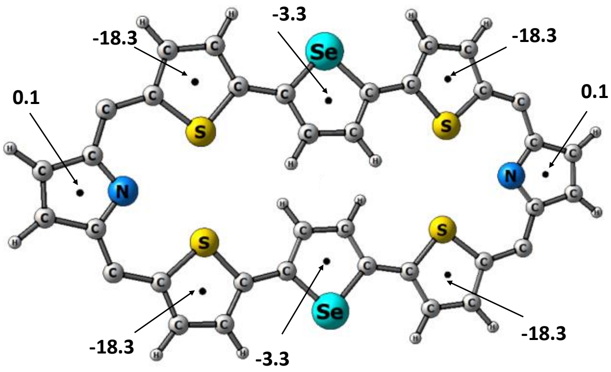<br>**NICS(0) values at M06L/6-31G** level**	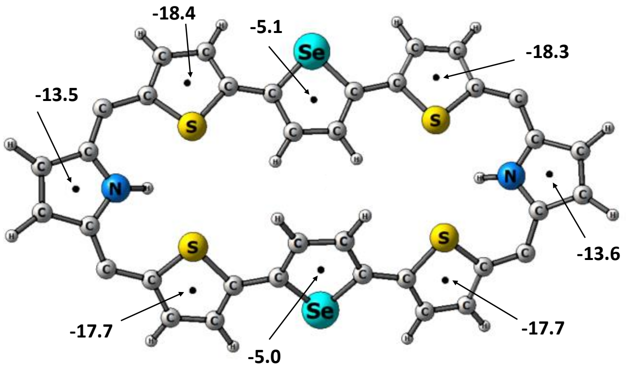<br>**NICS(0) values at M06L/6-31G** level**
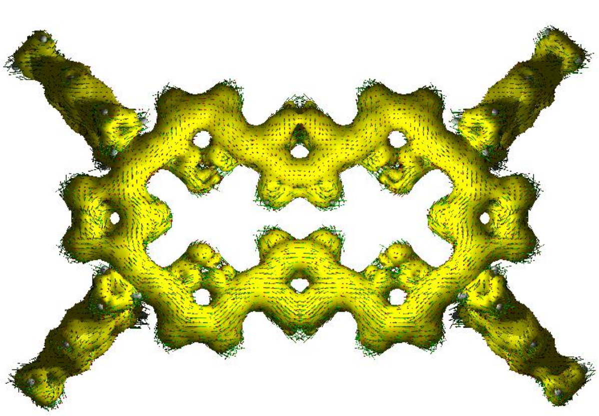<br>**AICD Plot - the current density vectors plotted on to isosurface of value 0.026.**	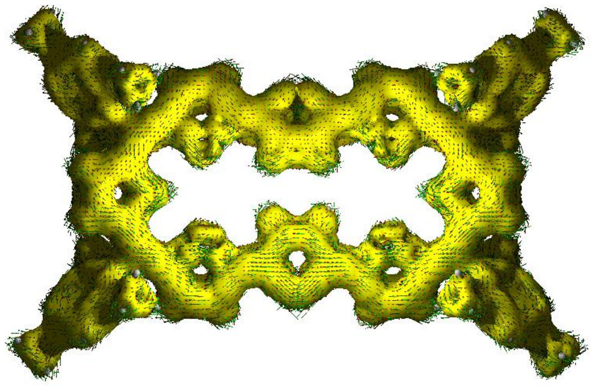<br>**AICD Plot - the current density vectors plotted on to isosurface of value 0.026.**

Chart S3: X-ray structure, Optimized geometry, NICS (0) values and AICD plots for **15A** and **15A**.2H^{+}

a)

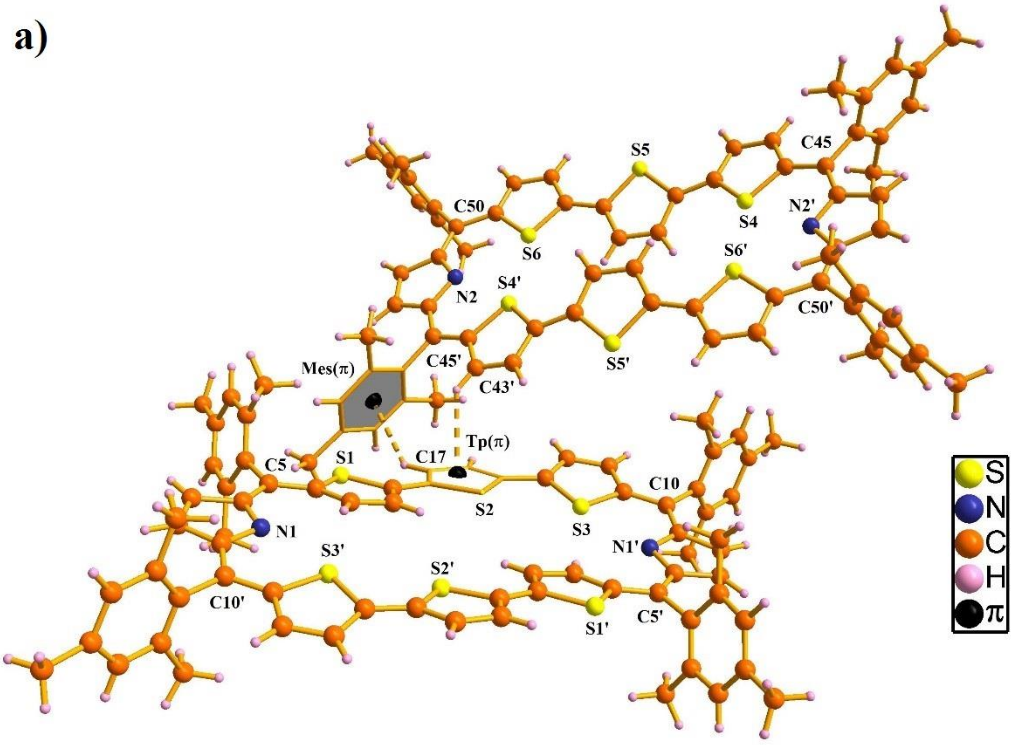

b)

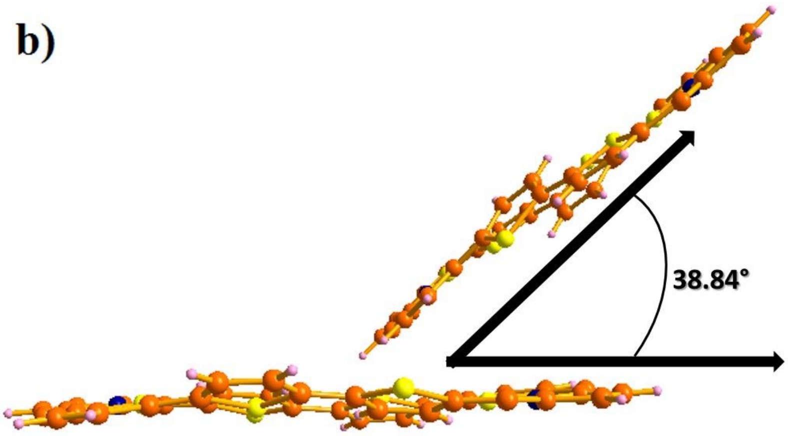

Figure S47: Single crystal X-ray structure of **10**. a) Top view and b) Side view (The *meso* mesityl substituents are omitted for clarity).

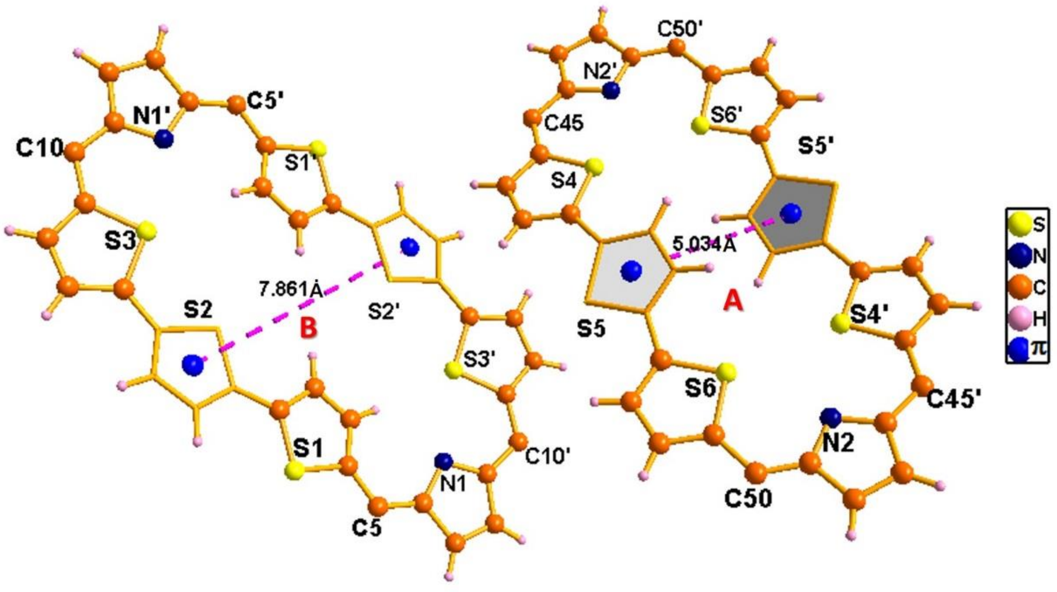

Figure S48: Distances between two middle thiophene centres of **10** (**A** and **B**). (The *meso* mesityl substituents are omitted for clarity).

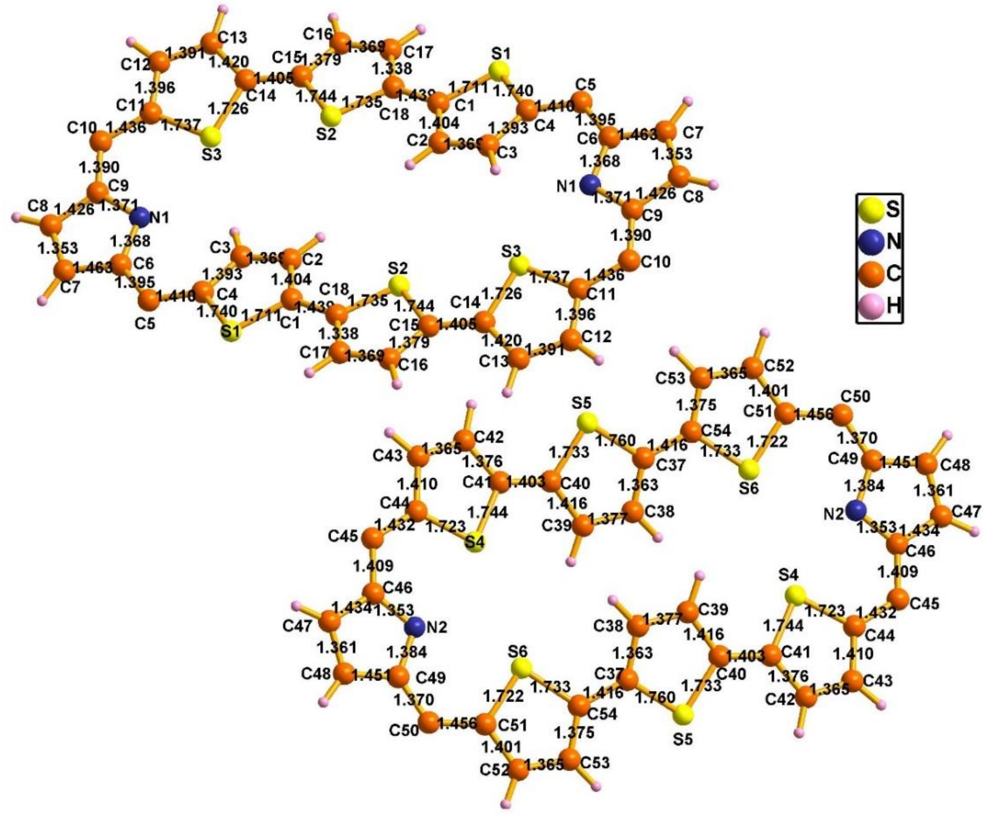

Figure S49: Bond length distances in **10**. (The *meso* mesityl substituents are omitted for clarity)

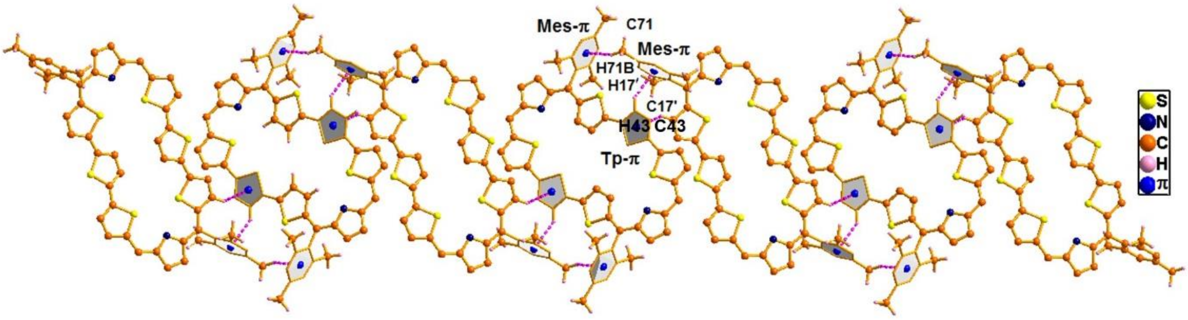

Mes(π)…C17’-H17’ : 2.71Å & 137.01°
Mes(π)…C71-H71B : 3.15Å & 150.91°
Tp(π)…C43-H43 : 2.83Å & 136.14°

Figure S50: One-dimensional array of **10**. (The *meso* mesityl substituents and hydrogen atoms which are not in part of interactions are omitted for clarity)

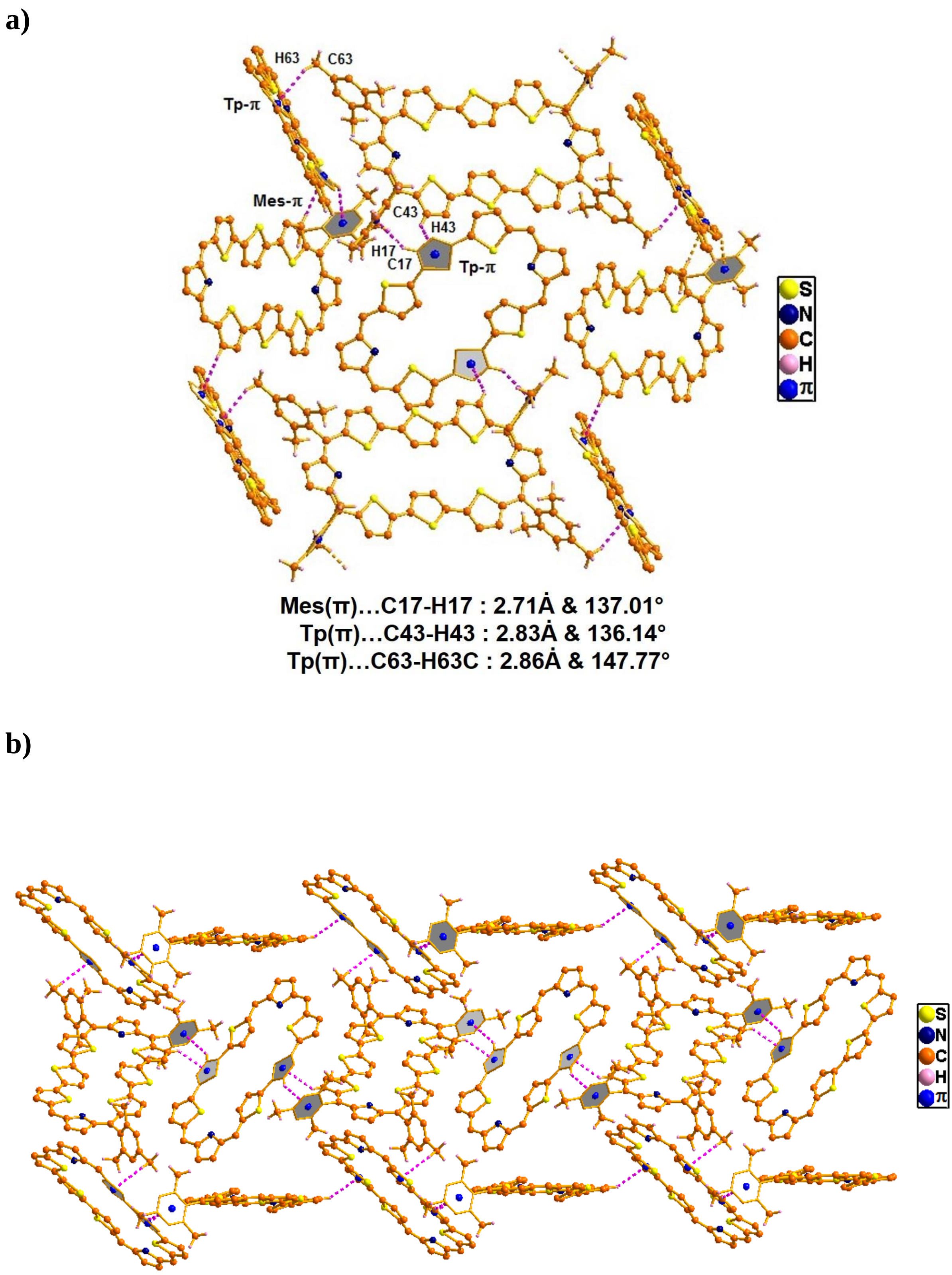

Figure S51: Two-dimensional array of **10**. (The *meso* mesityl substituents and hydrogen atoms which are not in part of interactions are omitted for clarity)

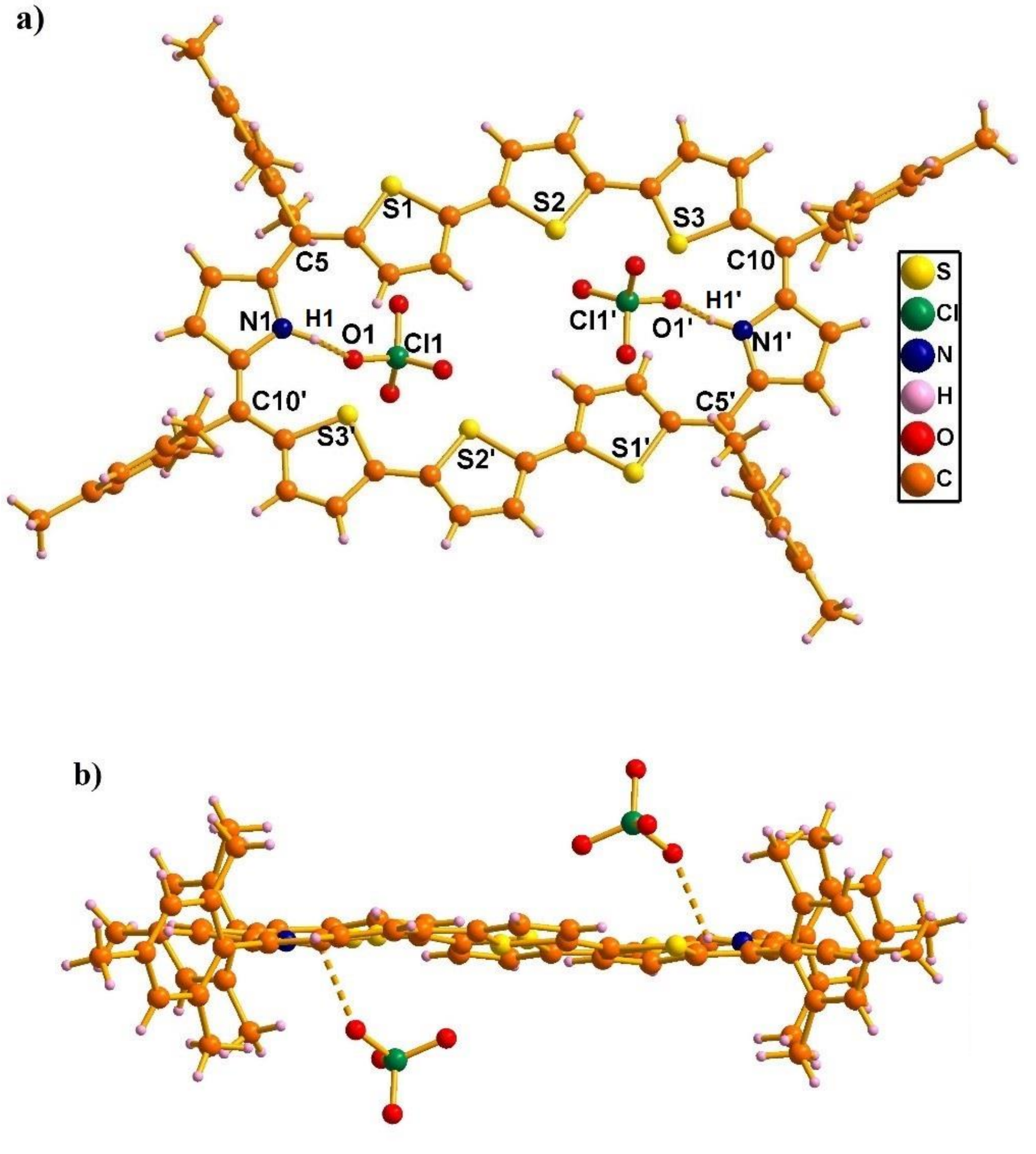

Figure S52: Single crystal X-ray structure (Top view and Side view) of **10B.2H$^+$**(**2ClO$_4^-$**).

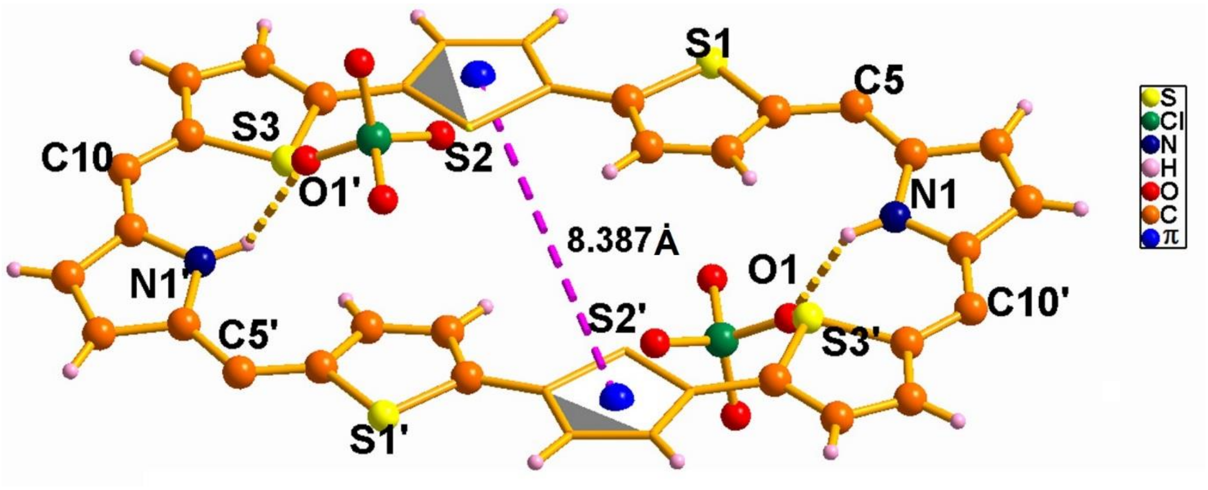

Figure S53: Distance between two middle thiophene centres of **10B**.2H$^+$. (The *meso* mesityl substituents are omitted for clarity).

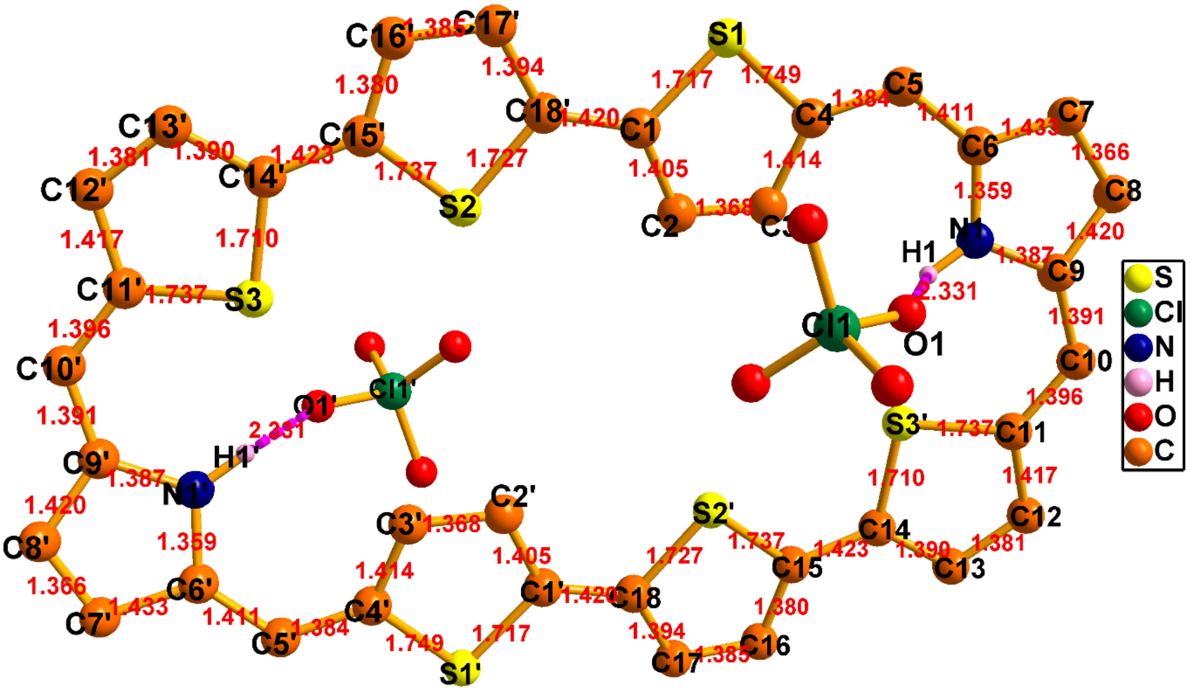

Figure S54: Bond length distances in **10B.**2H$^+$.(The *meso* mesityl substituents are omitted for clarity).

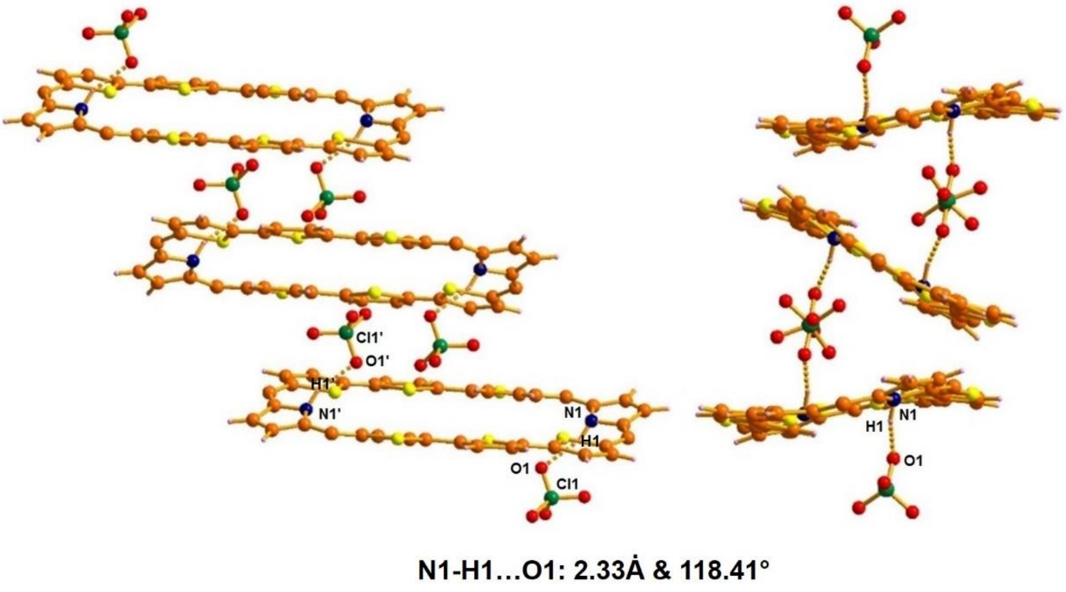

Figure S55: One-dimensional array of **10B.2H^{+}(2ClO$_4^{-}$)**. (The *meso* mesityl substituents are omitted for clarity).

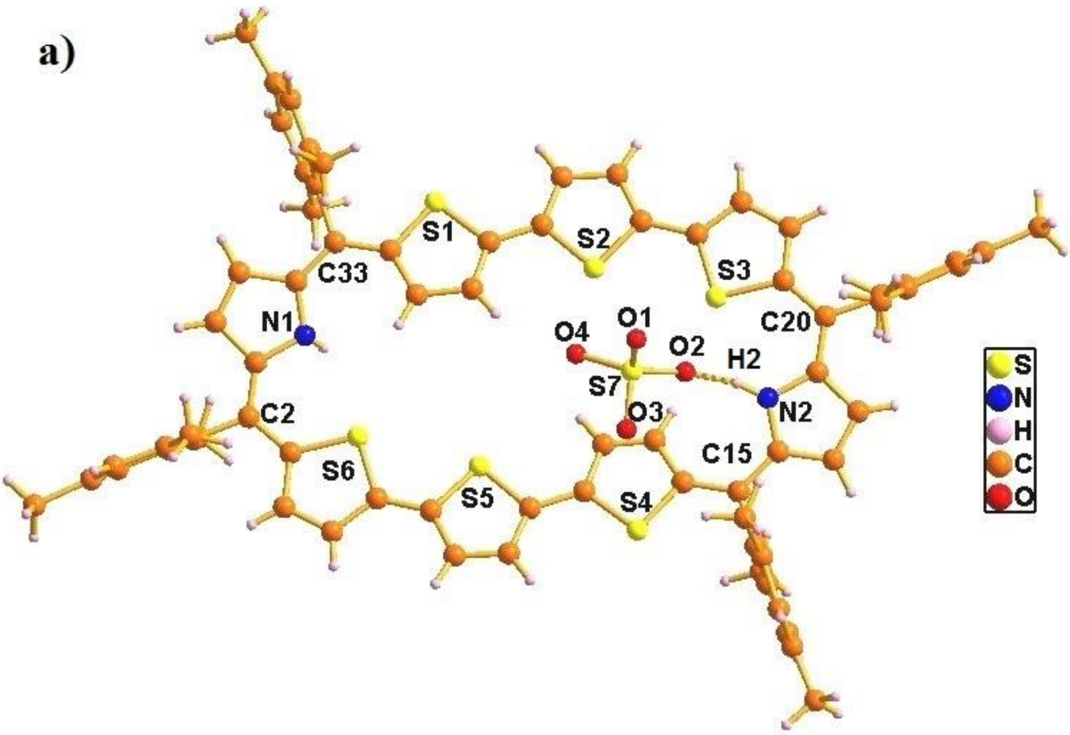

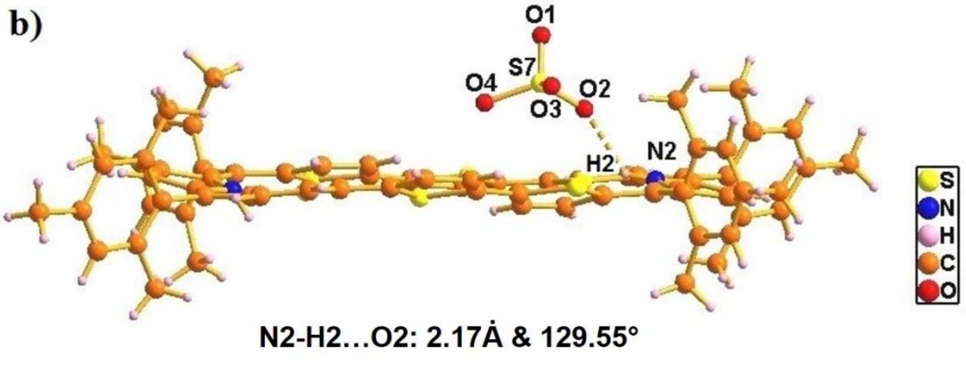

Figure S56: Single crystal X-ray structure of **10B.2H⁺**(SO_4^{2-}) a) Top view and b) Side view.

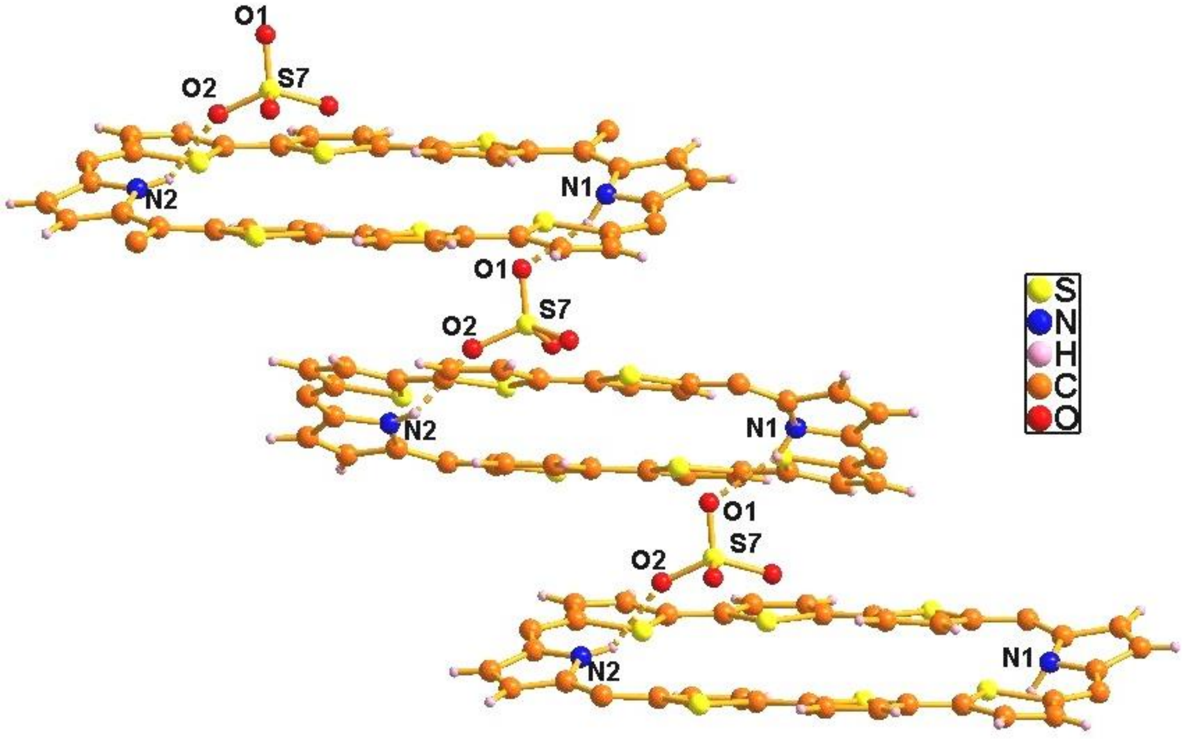

Figure S57: One-dimensional array of **10B**.2H^{+}(SO_4^{2-}). (The *meso* mesityl substituents are omitted for clarity).

TABLE S1**.** Crystallographic data for **10** and **10.**$2H^+$.

	**10**	**10.**$2H^+$	
T, K	100 K	100K	100K
Formula	C72 H60N2S6	C72 H62 N2 S6 Cl2 O8	C72H62N2S7O4
Formula weight	1145.58	1455.97	1351.60
Color and Habit	Dark brown	Dark green	Dark brown
Crystal system	Monoclinic	Triclinic	Monoclinic
Space group	$P2_1/n$	*P*-1	$P2_1/c$
a, Å	19.747(6)	14.477(3)	18.466(9)
b, Å	15.812(6)	17.107(3)	27.362(14)
c, Å	22.766(7)	24.756(5)	16.312(8)
α , deg	90	81.890(12)	90
β, deg	92.329(10)	79.104(12)	100.556(3)
γ, deg	90	76.504(12)	90
V, $Å^3$	7103(4)	5823.8(19)	8103.2(7)
Radiation (λ, Å)	Mo Kα (0.71073)	Mo Kα (0.71073)	Mo Kα (0.71073)
Z	4	1	4
d_{calcd}, g•cm^{-3}	1.071	1.245	1.108
μ, mm^{-1}	0.231	0.300	0.263
F(000)	2408	2282.0	2829
No. of unique reflns	13018	21219	17352
No. of params. refined	733	1352	824
GOF on F^2	0.906	0.949	1.149
$R1^a$ [$I > 2\sigma(I)$]	0.0794	0.0791	0.1460
$R1^a$ (all data)	0.1900	0.1622	0.4382
$wR2^b$ (all data)	0.1790	0.2061	0.4783

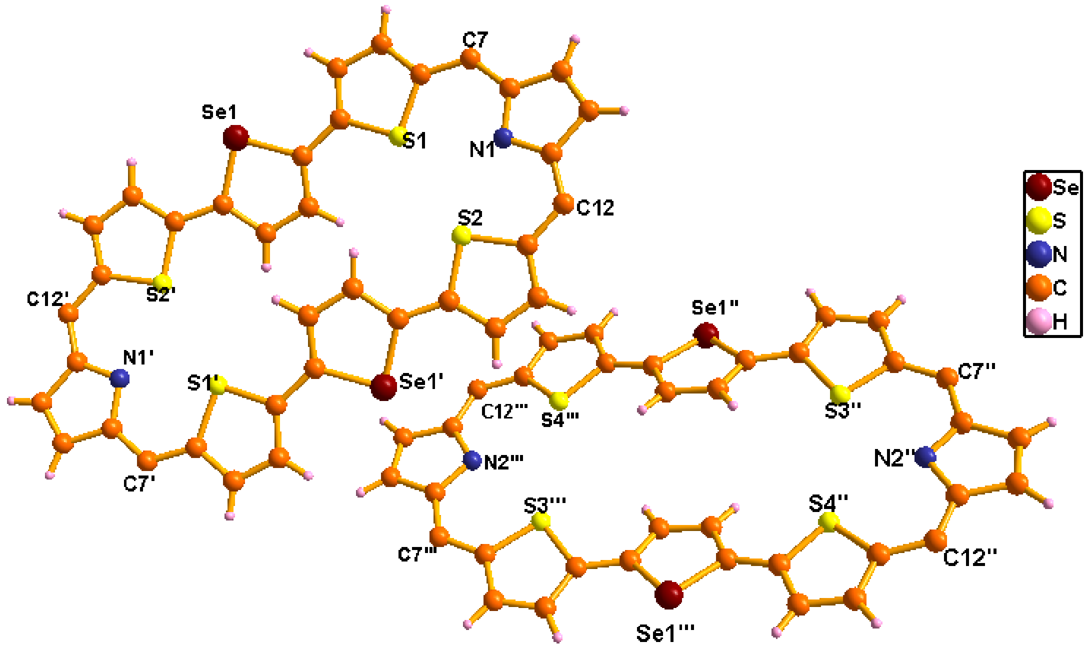

Figure S58: Single crystal X-ray structure of **15A** (Front View). (The *meso* mesityl substituents are omitted for clarity)

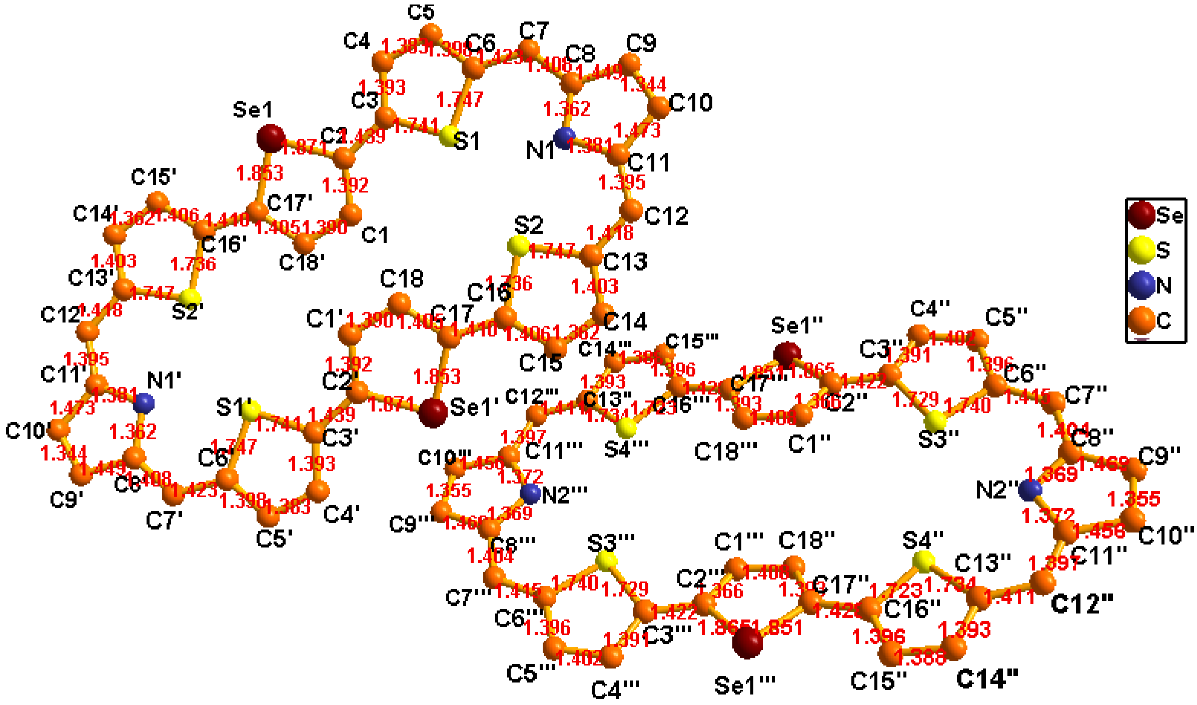

Figure S59: Bond length distances in **15A**. (The *meso* mesityl substituents are omitted for clarity).

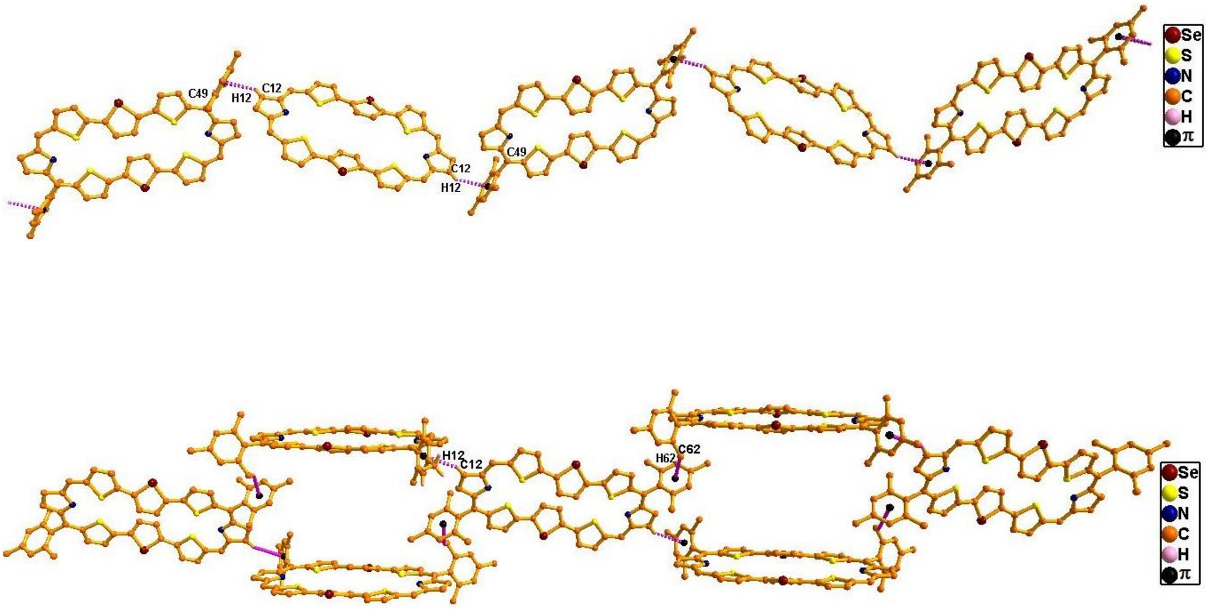

Mes(π)…C12-H12: 2.783Å & 141.82°

Mes(π)…C62-H62: 2.812Å & 136.29°

Figure S60: One-dimensional array of **15A**. (The *meso* mesityl substituents and hydrogen atoms which are not in part of interactions are omitted for clarity)

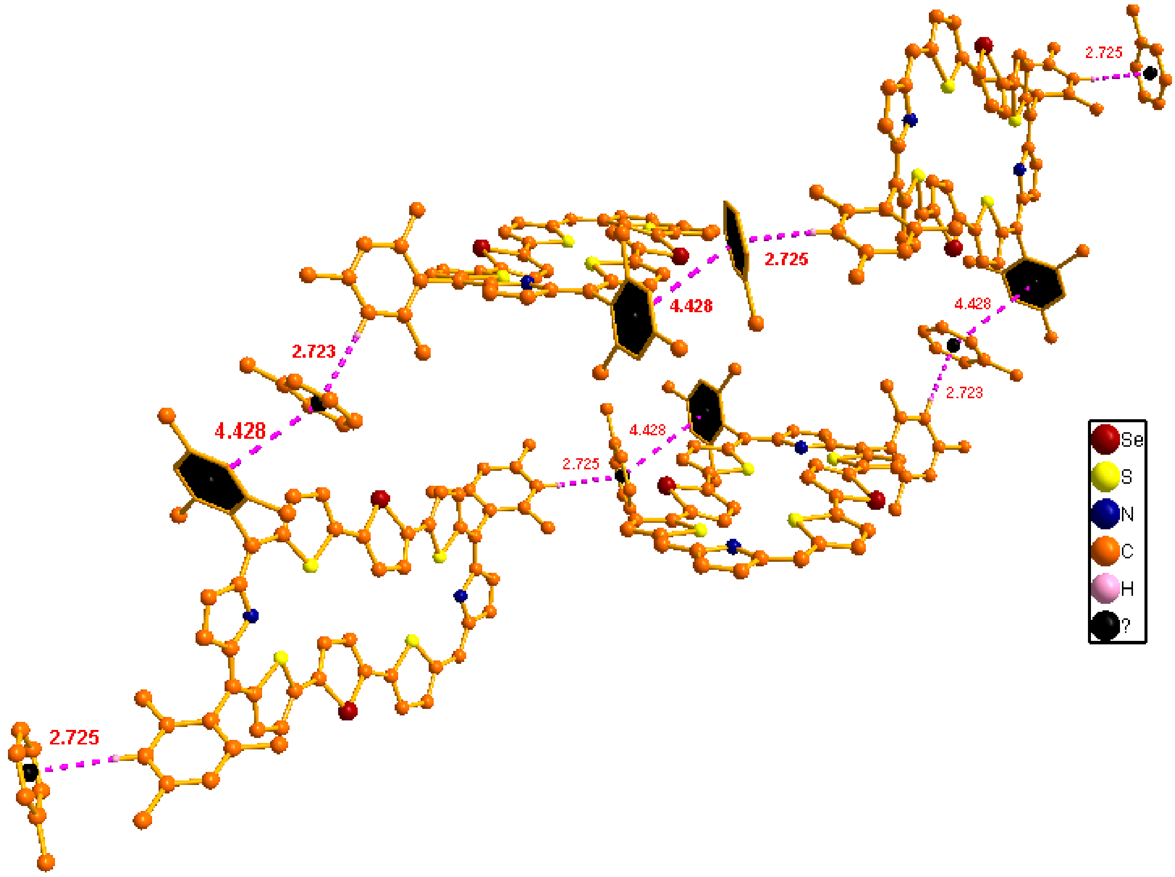

Figure S61: Two-dimensional array of **15A** (*Meso* mesityl substituents and hydrogens atoms which are not in part of interactions are omitted for clarity).

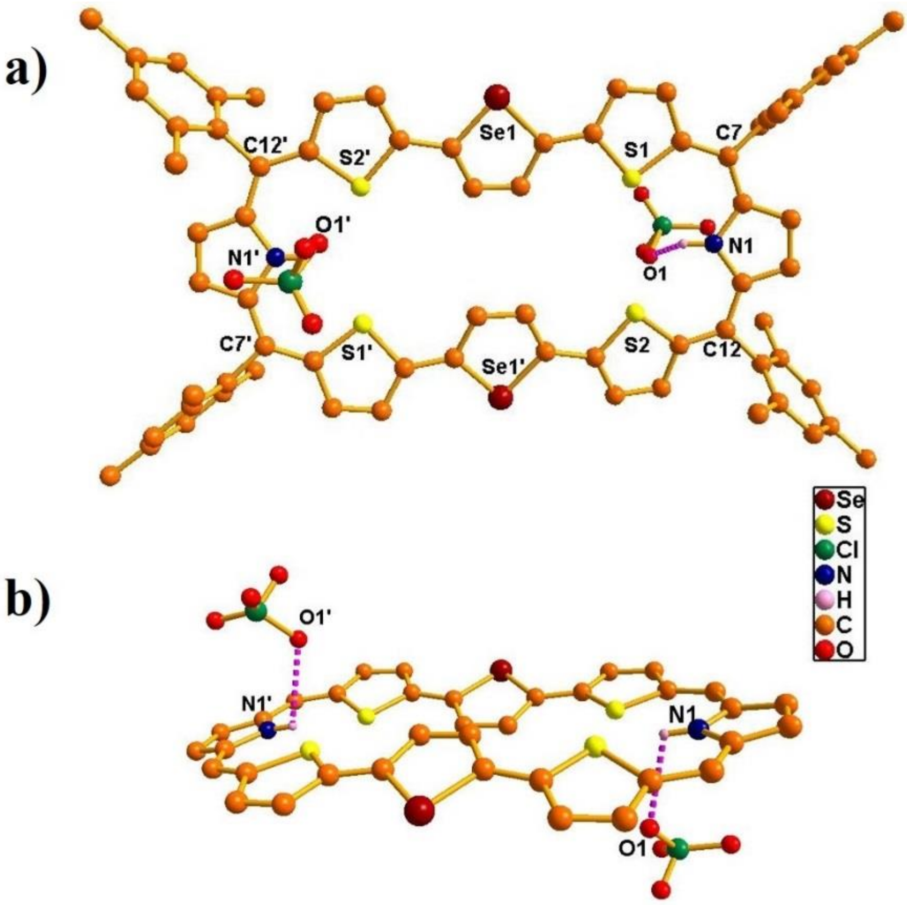

Figure S62: Single crystal X-ray structure of **15A**.2H$^+$(2ClO$_4^-$) a) Top view (hydrogen atoms are omitted) and b) Side view (*Meso* mesityl substituents and hydrogen atoms are omitted for clarity).

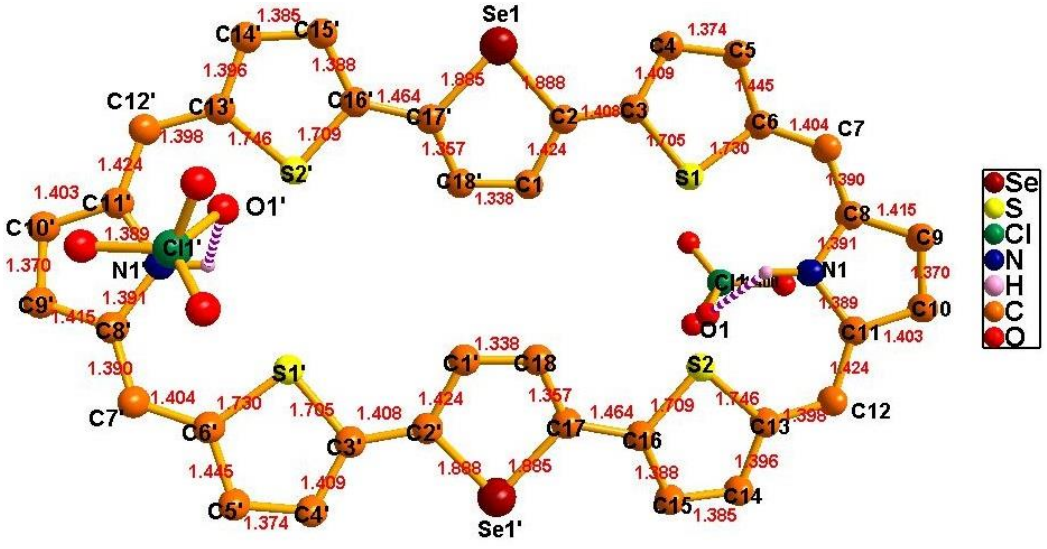

Figure S63: Bond length distances in **15A**.2H$^+$(2ClO$_4^-$). (The *meso* mesityl substituents are omitted for clarity)

TABLE S2**.** Crystallographic data for **15A** and **15A**.2H$^+$

	**15A**	**15A**.2H$^+$
T, K	100 K	113 K
Formula	$C_{72}H_{60}N_2S_4Se_2$	$C_{144}H_{124}Cl_6N_4O_{24}S_8Se_4$
Formula weight	1239.38	3079.48
Color and Habit	Dark brown	Golden
Crystal system	Monoclinic	triclinic
Space group	$P2_1/c$	*P*-1
a, Å	22.7958(4)	14.4054(5)
b, Å	11.8670(2)	18.9004(7)
c, Å	29.0481(4)	19.3632(6)
α , deg	90	107.869(3)
β, deg	93.89(10)	107.260(3)
γ, deg	90	105.339(3)
V, Å^3	7839.9(2)	4409.8(3)
Radiation (λ, Å)	CuKα (λ = 1.54184)	CuKα (λ = 1.54184)
Z	4	1
d_{calcd}, g•cm^{-3}	1.050	1.160
μ, mm^{-1}	2.436	3.180
F(000)	2552.0	1574.0
No. of unique reflns	56327	60529
No. of params. refined	14125	18011
GOF on F^2	1.056	1.609
*R*1[a] [$I > 2\sigma(I)$]	0.1062	0.1639
*R*1[a] (all data)	0.1207	0.2028
*wR*2[b] (all data)	0.3029	0.4114

TABLE S3. Total energy in a.u. and relative energy (E_{rel}) of '**B**' configuration with respect to '**A**' in kcal/mol.

Macrocycle	M06L/CC-pVTZ //M06L/6-31G**(a.u.)	E_{rel} (Kcal/mol)
**10A**	-5279.089075	0.0
**10B**	-5279.085954	2.0
**13A**	-7282.397194	0.0
**13B**	-7282.391794	3.4
**15A**	-9285.705583	0.0
***15B**	-9285.697999	4.8
***10A.2H$^+$**	-5279.873635	0.0
**10B.2H$^+$**	-5279.874464	-0.5
**13A.2H$^+$**	-7283.185147	0.0
**13B.2H$^+$**	-7283.179498	3.5
**15A.2H$^+$**	-9286.494571	0.0
***15B.2H$^+$**	-9286.485212	5.9

***10A**.2H$^+$, **15B** and **15B**.2H$^+$ were not formed in the reaction. As observed in other cases, the possible structures are proposed here and we have also done the energy minimized optimized structures of such macrocycles and their protonated derivatives.

<u>***10A**.2H$^+$</u>:

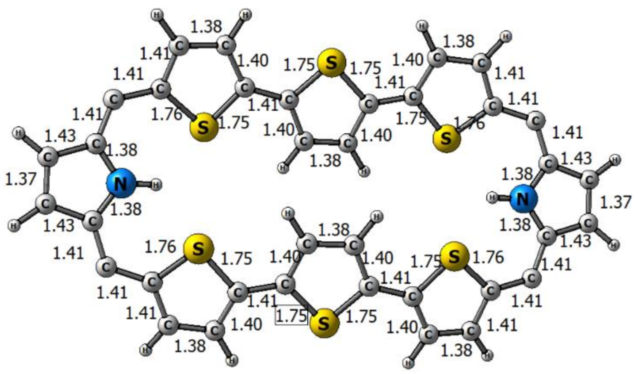

M06L/6-31G** level optimized geometry of **10A**.2H$^+$

***15B**:*

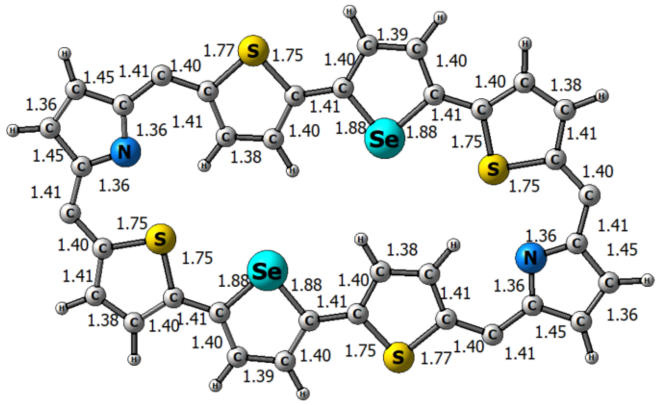

M06L/6-31G** level optimized geometry of **15B**

*__15B__.2H$^+$:

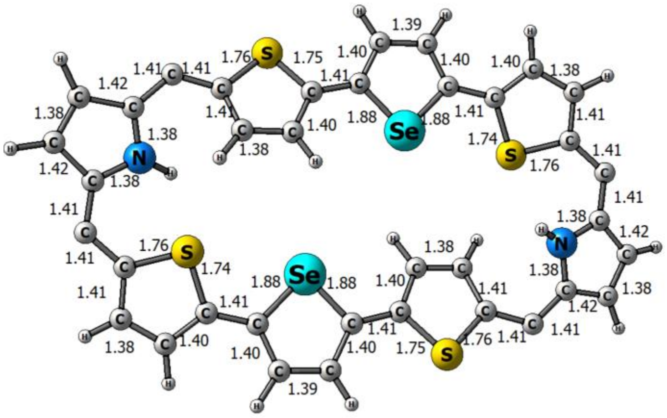

M06L/6-31G** level optimized geometry of **15B**.2H$^+$

Cartesian coordinates of optimized geometries at M06L/6-31G** level in Å.

10A:

S	3.800088000	1.667068000	0.298706000
S	3.800053000	-1.667062000	-0.298599000
S	-0.000018000	-3.856422000	-0.190764000
N	6.103972000	0.000011000	-0.000046000
C	8.024567000	-3.854724000	1.355709000
C	7.490391000	-3.505780000	0.101877000
C	7.909944000	-4.182803000	-1.057822000
C	6.477522000	-2.414735000	0.003154000
C	9.405274000	-5.560441000	0.290256000
C	5.126593000	2.793090000	0.092926000
C	6.926712000	1.080923000	-0.010499000
C	6.926702000	-1.080926000	0.010474000
C	6.477547000	2.414752000	-0.003127000
C	2.633254000	2.972354000	0.350497000
C	8.970397000	-4.878121000	1.426913000
H	9.375938000	-5.150996000	2.400668000
C	7.490435000	3.505766000	-0.101828000
C	4.634297000	4.114762000	0.050317000
H	5.304145000	4.959611000	-0.071742000
C	8.322264000	0.679176000	-0.011027000
H	9.170498000	1.353062000	-0.017610000
C	8.860741000	-5.197559000	-0.941956000
H	9.185602000	-5.717613000	-1.842574000
C	-1.261159000	-2.679415000	-0.504750000
C	7.909586000	4.183162000	1.057807000
C	8.322256000	-0.679190000	0.011075000

H	9.170478000	-1.353092000	0.017721000
C	2.633223000	-2.972358000	-0.350433000
C	5.126565000	-2.793088000	-0.092864000
C	3.260866000	4.213483000	0.182952000
H	2.712994000	5.150462000	0.182096000
C	7.580981000	-3.143638000	2.599099000
H	7.869131000	-2.086335000	2.582341000
H	8.018486000	-3.596369000	3.492133000
H	6.491029000	-3.160748000	2.707584000
C	3.260839000	-4.213482000	-0.182908000
H	2.712980000	-5.150469000	-0.182070000
C	4.634276000	-4.114758000	-0.050269000
H	5.304117000	-4.959619000	0.071747000
C	7.353136000	-3.815382000	-2.400858000
H	6.277788000	-4.017876000	-2.463579000
H	7.846249000	-4.373082000	-3.200590000
H	7.474326000	-2.746188000	-2.606456000
C	1.261142000	-2.679412000	-0.504661000
C	7.352278000	3.816240000	2.400774000
H	6.277113000	4.019734000	2.463408000
H	7.845805000	4.373503000	3.200556000
H	7.472460000	2.746938000	2.606387000
C	0.692820000	-1.450721000	-0.856507000
H	1.301300000	-0.591405000	-1.122402000
C	8.860452000	5.197848000	0.941964000
H	9.184993000	5.718173000	1.842541000
C	-0.692814000	-1.450725000	-0.856566000
H	-1.301275000	-0.591407000	-1.122495000
C	8.025081000	3.854296000	-1.355573000

C	8.970957000	4.877658000	-1.426758000
H	9.376839000	5.150239000	-2.400454000
C	7.582032000	3.142786000	-2.598915000
H	7.871617000	2.085863000	-2.582399000
H	8.018699000	3.596191000	-3.492018000
H	6.492035000	3.158447000	-2.707065000
C	10.447229000	-6.633316000	0.386789000
H	10.402447000	-7.156137000	1.346216000
H	11.457627000	-6.217127000	0.298581000
H	10.338981000	-7.375681000	-0.408759000
C	9.405446000	5.560325000	-0.290168000
C	10.447451000	6.633156000	-0.386646000
H	10.337785000	7.376780000	0.407536000
H	10.404225000	7.154438000	-1.346974000
H	11.457740000	6.217222000	-0.296024000
S	-3.800087000	-1.667075000	-0.298870000
S	-3.800050000	1.667074000	0.298754000
S	0.000019000	3.856429000	0.190767000
N	-6.103967000	-0.000009000	0.000049000
C	-8.024568000	3.854771000	-1.355518000
C	-7.490395000	3.505783000	-0.101698000
C	-7.909957000	4.182757000	1.058025000
C	-6.477521000	2.414739000	-0.003014000
C	-9.405290000	5.560439000	-0.290005000
C	-5.126594000	-2.793094000	-0.093090000
C	-6.926707000	-1.080923000	0.010443000
C	-6.926697000	1.080928000	-0.010394000
C	-6.477545000	-2.414753000	0.002991000
C	-2.633250000	-2.972360000	-0.350622000

C	-8.970405000	4.878166000	-1.426687000
H	-9.375944000	5.151075000	-2.400433000
C	-7.490433000	-3.505771000	0.101654000
C	-4.634305000	-4.114771000	-0.050529000
H	-5.304157000	-4.959622000	0.071499000
C	-8.322259000	-0.679175000	0.011035000
H	-9.170492000	-1.353062000	0.017606000
C	-8.860759000	5.197511000	0.942195000
H	-9.185628000	5.717529000	1.842832000
C	1.261169000	2.679415000	0.504687000
C	-7.909676000	-4.183042000	-1.058024000
C	-8.322250000	0.679191000	-0.011025000
H	-9.170472000	1.353093000	-0.017636000
C	-2.633219000	2.972371000	0.350543000
C	-5.126566000	2.793096000	0.093016000
C	-3.260870000	-4.213493000	-0.183138000
H	-2.713000000	-5.150474000	-0.182292000
C	-7.580975000	3.143735000	-2.598934000
H	-7.869114000	2.086428000	-2.582215000
H	-8.018486000	3.596493000	-3.491951000
H	-6.491024000	3.160861000	-2.707419000
C	-3.260844000	4.213496000	0.183068000
H	-2.712988000	5.150484000	0.182233000
C	-4.634283000	4.114769000	0.050458000
H	-5.304129000	4.959629000	-0.071532000
C	-7.353155000	3.815286000	2.401049000
H	-6.277807000	4.017776000	2.463782000
H	-7.846270000	4.372956000	3.200800000
H	-7.474346000	2.746085000	2.606606000

C	-1.261133000	2.679423000	0.504715000
C	-7.352462000	-3.815976000	-2.400991000
H	-6.277283000	-4.019374000	-2.463688000
H	-7.845982000	-4.373219000	-3.200791000
H	-7.472743000	-2.746667000	-2.606519000
C	-0.692799000	1.450734000	0.856548000
H	-1.301271000	0.591423000	1.122478000
C	-8.860538000	-5.197732000	-0.942219000
H	-9.185147000	-5.717961000	-1.842828000
C	0.692834000	1.450728000	0.856530000
H	1.301303000	0.591405000	1.122428000
C	-8.024985000	-3.854430000	1.355399000
C	-8.970864000	-4.877797000	1.426546000
H	-9.376673000	-5.150481000	2.400243000
C	-7.581847000	-3.143048000	2.598783000
H	-7.871529000	-2.086148000	2.582447000
H	-8.018369000	-3.596609000	3.491879000
H	-6.491836000	-3.158628000	2.706800000
C	-10.447250000	6.633312000	-0.386499000
H	-10.402513000	7.156124000	-1.345933000
H	-11.457644000	6.217123000	-0.298240000
H	-10.338967000	7.375683000	0.409037000
C	-9.405442000	-5.560338000	0.289919000
C	-10.447444000	-6.633176000	0.386349000
H	-10.337455000	-7.377051000	-0.407555000
H	-10.404556000	-7.154148000	1.346859000
H	-11.457714000	-6.217309000	0.295201000

10B

S	4.379702000	2.000111000	0.066479000
S	2.925616000	-3.965319000	-0.020517000
S	1.211660000	2.472032000	0.173309000
N	5.933684000	-0.304384000	-0.061100000
C	7.102997000	0.381715000	-0.044687000
C	9.176805000	2.644086000	-1.256485000
C	8.496691000	2.454883000	-0.039893000
C	-0.186275000	3.528146000	0.127830000
C	7.162774000	1.788340000	-0.028859000
C	6.004031000	4.019837000	-0.030789000
H	6.926157000	4.590375000	-0.075122000
C	2.320427000	3.822928000	0.079552000
C	6.022283000	2.608558000	-0.004418000
C	4.738273000	4.576506000	-0.001614000
H	4.543711000	5.643367000	-0.017914000
C	6.277939000	-1.616803000	-0.055833000
C	9.071390000	2.893507000	1.166850000
C	0.227163000	4.863088000	0.045234000
H	-0.482862000	5.682571000	0.006559000
C	7.723860000	-1.773648000	-0.034882000
H	8.253761000	-2.718749000	-0.029020000
C	3.715753000	3.616629000	0.053266000
C	1.605101000	5.025929000	0.022155000
H	2.100190000	5.988534000	-0.040475000
C	6.611220000	-6.134577000	-1.036730000
H	6.760627000	-6.717355000	-1.945200000
C	5.366240000	-2.697123000	-0.065382000
C	10.423497000	3.270865000	-1.243751000

H	10.944652000	3.421896000	-2.188576000
C	5.913933000	-4.082019000	0.025618000
C	8.242199000	-0.516388000	-0.031187000
H	9.283351000	-0.217062000	-0.014747000
C	6.103096000	-4.841516000	-1.145535000
C	6.935148000	-6.697583000	0.199803000
C	1.485495000	-2.985658000	-0.161995000
C	1.829393000	-1.631895000	-0.303094000
H	1.078129000	-0.856757000	-0.424095000
C	3.971534000	-2.545682000	-0.137972000
C	11.015512000	3.710350000	-0.059274000
C	6.238069000	-4.625040000	1.280992000
C	6.741035000	-5.926114000	1.344201000
H	6.987844000	-6.346688000	2.318369000
C	3.186535000	-1.386701000	-0.292181000
H	3.648741000	-0.415381000	-0.400120000
C	10.321109000	3.513308000	1.134736000
H	10.763858000	3.850660000	2.071375000
C	8.570136000	2.185022000	-2.548620000
H	8.446015000	1.096393000	-2.570520000
H	9.191017000	2.469407000	-3.401558000
H	7.571087000	2.608771000	-2.699069000
C	8.356020000	2.693052000	2.469331000
H	7.405283000	3.237544000	2.497724000
H	8.962916000	3.032729000	3.311973000
H	8.107895000	1.638503000	2.632356000
C	7.476438000	-8.092525000	0.286938000
H	6.769937000	-8.824627000	-0.118778000
H	7.691243000	-8.377668000	1.319866000

H	8.402619000	-8.201853000	-0.287465000
C	12.370117000	4.351560000	-0.066271000
H	13.165460000	3.605096000	0.043743000
H	12.485968000	5.062206000	0.756753000
H	12.561443000	4.883430000	-1.002385000
C	5.778078000	-4.260491000	-2.488732000
H	6.011779000	-4.963063000	-3.292107000
H	6.337753000	-3.336535000	-2.671882000
H	4.716903000	-3.998264000	-2.569282000
C	6.052157000	-3.820207000	2.532096000
H	6.714485000	-2.947017000	2.549677000
H	6.261102000	-4.417739000	3.422642000
H	5.031303000	-3.431737000	2.615381000
S	-4.379956000	-2.000044000	-0.063817000
S	-2.925761000	3.965316000	0.022851000
S	-1.211904000	-2.471987000	-0.168371000
N	-5.933980000	0.304493000	0.062925000
C	-7.103343000	-0.381595000	0.045606000
C	-9.178228000	-2.644171000	1.255658000
C	-8.497006000	-2.454777000	0.039713000
C	0.186008000	-3.528114000	-0.122809000
C	-7.163110000	-1.788173000	0.029941000
C	-6.004399000	-4.019686000	0.032556000
H	-6.926557000	-4.590211000	0.076244000
C	-2.320679000	-3.822882000	-0.074933000
C	-6.022582000	-2.608438000	0.006352000
C	-4.738612000	-4.576400000	0.004386000
H	-4.544095000	-5.643261000	0.020918000
C	-6.278158000	1.616869000	0.056621000

C	-9.070494000	-2.893398000	-1.167594000
C	-0.227420000	-4.863018000	-0.040116000
H	0.482579000	-5.682505000	-0.001154000
C	-7.724053000	1.773836000	0.034112000
H	-8.253888000	2.718984000	0.027344000
C	-3.716048000	-3.616558000	-0.049503000
C	-1.605386000	-5.025851000	-0.017221000
H	-2.100466000	-5.988455000	0.045446000
C	-6.617149000	6.134865000	1.029977000
H	-6.772969000	6.717516000	1.937324000
C	-5.366399000	2.697196000	0.066192000
C	-10.424767000	-3.271200000	1.241716000
H	-10.946777000	-3.422391000	2.186044000
C	-5.913626000	4.082120000	-0.027268000
C	-8.242450000	0.516591000	0.030285000
H	-9.283595000	0.217306000	0.012763000
C	-6.106075000	4.841947000	1.141676000
C	-6.937785000	6.696052000	-0.206805000
C	-1.485760000	2.985745000	0.166575000
C	-1.829846000	1.632088000	0.308521000
H	-1.078688000	0.857074000	0.431020000
C	-3.971836000	2.545814000	0.140715000
C	-11.015572000	-3.710763000	0.056652000
C	-6.238630000	4.622845000	-1.284775000
C	-6.743596000	5.921746000	-1.350801000
H	-6.994247000	6.338378000	-2.325764000
C	-3.186966000	1.386921000	0.296600000
H	-3.649360000	0.415759000	0.405189000
C	-10.320114000	-3.513472000	-1.136689000

H -10.761926000 -3.850841000 -2.073763000

C -8.572829000 -2.185079000 2.548375000

H -8.448752000 -1.096452000 2.570392000

H -9.194524000 -2.469469000 3.400718000

H -7.573921000 -2.608808000 2.699802000

C -8.353997000 -2.692620000 -2.469404000

H -7.402877000 -3.236487000 -2.496804000

H -8.959863000 -3.032764000 -3.312597000

H -8.106400000 -1.637924000 -2.632292000

C -7.453413000 8.099802000 -0.304115000

H -8.070773000 8.365338000 0.558867000

H -6.631607000 8.824547000 -0.340725000

H -8.051310000 8.249755000 -1.207165000

C -12.370026000 -4.352306000 0.062404000

H -13.165578000 -3.605736000 -0.045331000

H -12.485963000 -5.060826000 -0.762430000

H -12.560931000 -4.886667000 0.997193000

C -5.785682000 4.262930000 2.486834000

H -6.022883000 4.966391000 3.288412000

H -6.344935000 3.338611000 2.669386000

H -4.724462000 4.002110000 2.571550000

C -6.053540000 3.813949000 -2.533372000

H -6.711613000 2.937475000 -2.545186000

H -6.268189000 4.407049000 -3.425521000

H -5.031063000 3.430111000 -2.618562000

13A:

Se 0.000030000 -3.980374000 -0.094281000

S 3.833270000 -1.581551000 -0.251648000

S	-3.833567000	-1.582150000	-0.253211000
N	6.128392000	0.127208000	0.004396000
C	7.561133000	-3.360230000	0.086392000
C	6.530533000	-2.284054000	0.004525000
C	6.962909000	-0.943860000	0.005212000
C	7.976508000	-4.026546000	-1.082527000
C	5.185477000	-2.683010000	-0.070079000
C	9.516370000	-5.379586000	0.239733000
C	1.308781000	-2.666017000	-0.408592000
C	4.719876000	-4.015203000	-0.023840000
H	5.408172000	-4.846790000	0.084878000
C	3.348367000	-4.138715000	-0.133075000
H	2.816458000	-5.085878000	-0.126015000
C	-2.689852000	-2.910526000	-0.285224000
C	8.122343000	-3.699759000	1.329398000
C	8.947597000	-5.022070000	-0.984675000
H	9.272002000	-5.529329000	-1.892853000
C	2.689783000	-2.910109000	-0.284512000
C	-1.308863000	-2.666239000	-0.408949000
C	6.940220000	1.217110000	-0.015926000
C	9.089946000	-4.705508000	1.382802000
H	9.521391000	-4.968238000	2.348081000
C	0.693220000	-1.451471000	-0.725823000
H	1.281666000	-0.572105000	-0.975341000
C	6.478607000	2.545748000	-0.006198000
C	8.354285000	-0.527624000	-0.011093000
H	9.209574000	-1.192523000	-0.014824000
C	-0.693415000	-1.451594000	-0.726035000
H	-1.281938000	-0.572328000	-0.975726000

C	-5.185686000	-2.683752000	-0.071731000
C	7.480731000	3.646397000	-0.110537000
C	8.339731000	0.830519000	-0.032398000
H	9.180569000	1.513457000	-0.048944000
C	7.395536000	-3.661382000	-2.415903000
H	7.499891000	-2.589973000	-2.619328000
H	6.322004000	-3.877201000	-2.463264000
H	7.883740000	-4.209975000	-3.224891000
C	7.912010000	4.315755000	1.048762000
C	-3.348292000	-4.139181000	-0.133455000
H	-2.816233000	-5.086256000	-0.125826000
C	10.549195000	-6.462916000	0.317601000
H	11.368115000	-6.291548000	-0.388498000
H	10.124155000	-7.442157000	0.070600000
H	10.981481000	-6.536611000	1.318661000
C	7.691562000	-2.994878000	2.580744000
H	6.604425000	-3.030665000	2.710800000
H	7.960942000	-1.932741000	2.557171000
H	8.154349000	-3.438882000	3.465370000
C	8.000817000	4.004349000	-1.368064000
C	8.942729000	5.030602000	-1.443068000
H	9.342969000	5.305048000	-2.418605000
C	-4.719840000	-4.015842000	-0.024655000
H	-5.408044000	-4.847484000	0.084145000
C	9.384152000	5.710693000	-0.307103000
C	8.858788000	5.334304000	0.928938000
H	9.198309000	5.843959000	1.830059000
C	7.378424000	3.930983000	2.396310000
H	7.521334000	2.863078000	2.594766000

H	6.300446000	4.113653000	2.472568000
H	7.870898000	4.493236000	3.193235000
C	7.553002000	3.292727000	-2.609622000
H	6.461902000	3.298337000	-2.706999000
H	7.852860000	2.238573000	-2.599851000
H	7.976417000	3.753577000	-3.505288000
C	10.376660000	6.828321000	-0.416682000
H	9.886316000	7.774430000	-0.674135000
H	11.119027000	6.635731000	-1.196563000
H	10.909210000	6.989706000	0.524358000
S	-0.000322000	3.952163000	0.194829000
S	-3.806902000	1.776915000	0.303533000
S	3.806669000	1.777497000	0.305299000
N	-6.128670000	0.126434000	0.002173000
C	-7.481080000	3.645669000	-0.112322000
C	-6.478929000	2.544928000	-0.008297000
C	-6.940515000	1.216369000	-0.019334000
C	-7.916086000	4.311433000	1.048203000
C	-5.125240000	2.912355000	0.095351000
C	-9.374420000	5.719057000	-0.309267000
C	-1.261010000	2.776701000	0.513088000
C	-4.624742000	4.230550000	0.056158000
H	-5.288853000	5.079823000	-0.066181000
C	-3.250936000	4.319934000	0.191604000
H	-2.696580000	5.253077000	0.193219000
C	2.631759000	3.074897000	0.358693000
C	-7.988369000	4.015329000	-1.371066000
C	-8.855871000	5.335742000	0.928127000
H	-9.192393000	5.847440000	1.829263000

C	-2.632306000	3.074541000	0.358299000
C	1.260492000	2.776891000	0.513323000
C	-6.963151000	-0.944602000	0.001614000
C	-8.924195000	5.047796000	-1.446380000
H	-9.309429000	5.336602000	-2.423769000
C	-0.693384000	1.549116000	0.868280000
H	-1.301955000	0.691511000	1.139266000
C	-6.530798000	-2.284873000	0.001885000
C	-8.339945000	0.829721000	-0.038276000
H	-9.180706000	1.512669000	-0.056641000
C	0.692978000	1.549229000	0.868447000
H	1.301617000	0.691709000	1.139554000
C	5.124804000	2.913030000	0.096332000
C	-7.561478000	-3.360931000	0.083729000
C	-8.354523000	-0.528423000	-0.017047000
H	-9.209792000	-1.193350000	-0.022605000
C	-7.386039000	3.921983000	2.395789000
H	-7.533656000	2.854219000	2.591545000
H	-6.307441000	4.100012000	2.474226000
H	-7.877726000	4.484294000	3.193155000
C	-7.974053000	-4.029549000	-1.082969000
C	3.250140000	4.320271000	0.190868000
H	2.695555000	5.253281000	0.191396000
C	-10.405213000	6.802229000	-0.410589000
H	-11.419679000	6.397495000	-0.316045000
H	-10.287003000	7.549044000	0.379463000
H	-10.357448000	7.317759000	-1.373729000
C	-7.527905000	3.315940000	-2.614937000
H	-6.435921000	3.322683000	-2.701858000

H	-7.827577000	2.261710000	-2.618179000
H	-7.942921000	3.785023000	-3.510264000
C	-8.128348000	-3.695262000	1.327415000
C	-9.096919000	-4.697856000	1.381795000
H	-9.533336000	-4.953958000	2.346737000
C	4.623960000	4.231031000	0.055579000
H	5.287934000	5.080260000	-0.067586000
C	-9.519926000	-5.377045000	0.238425000
C	-8.948337000	-5.024023000	-0.984017000
H	-9.273201000	-5.532604000	-1.891137000
C	-7.391198000	-3.669493000	-2.416911000
H	-7.488194000	-2.597656000	-2.621558000
H	-6.319217000	-3.892960000	-2.464610000
H	-7.883329000	-4.215728000	-3.225126000
C	-7.701107000	-2.984080000	2.576416000
H	-6.614654000	-3.022831000	2.711449000
H	-7.966491000	-1.921160000	2.544874000
H	-8.169355000	-3.421145000	3.461614000
C	-10.541823000	-6.469829000	0.326078000
H	-10.078174000	-7.431384000	0.575797000
H	-11.285824000	-6.267307000	1.101756000
H	-11.069874000	-6.606622000	-0.621440000

13B:

S	4.440773000	2.008327000	0.033464000
S	2.976665000	-3.882427000	-0.044007000
S	1.257190000	2.468932000	0.058533000
N	6.016448000	-0.276290000	-0.103211000
C	6.363802000	-1.587280000	-0.100358000

C	5.982578000	-4.050861000	0.025354000
C	7.182058000	0.413899000	-0.053366000
C	7.229843000	1.820067000	-0.007677000
C	8.557747000	2.497270000	0.028018000
C	7.808953000	-1.740048000	-0.049370000
H	8.341459000	-2.683739000	-0.040471000
C	5.449418000	-2.666554000	-0.123333000
C	6.081018000	2.629922000	0.011763000
C	9.094341000	2.923871000	1.256707000
C	1.655756000	5.026630000	0.038288000
H	2.152705000	5.990289000	0.032292000
C	1.562889000	-2.878094000	-0.273193000
C	-0.138739000	3.529158000	0.042937000
C	6.052582000	4.040880000	0.022154000
H	6.971114000	4.618786000	0.011932000
C	3.765927000	3.620190000	0.046086000
C	4.060566000	-2.500853000	-0.245519000
C	8.323499000	-0.480847000	-0.024043000
H	9.363239000	-0.179105000	0.019268000
C	0.277519000	4.865479000	0.032997000
H	-0.430948000	5.687160000	0.023968000
C	4.782407000	4.587994000	0.040165000
H	4.580641000	5.653603000	0.047449000
C	10.343093000	3.545846000	1.269681000
H	10.759642000	3.866623000	2.223990000
C	2.369141000	3.821464000	0.049899000
C	6.329991000	-4.535450000	1.301871000
C	9.276288000	2.697121000	-1.164448000
C	3.308878000	-1.338031000	-0.503487000

H	3.799792000	-0.392078000	-0.682615000
C	6.133918000	-4.870515000	-1.107364000
C	8.344407000	2.699628000	2.535704000
H	7.391896000	3.241627000	2.547345000
H	8.927322000	3.026645000	3.400007000
H	8.094532000	1.642008000	2.674384000
C	6.814219000	-5.836872000	1.420792000
H	7.074902000	-6.210676000	2.410508000
C	6.971580000	-6.669715000	0.310732000
C	10.520969000	3.325600000	-1.106472000
H	11.075239000	3.478425000	-2.031941000
C	6.628104000	-6.165886000	-0.942551000
H	6.750045000	-6.796998000	-1.822107000
C	6.180810000	-3.666273000	2.514533000
H	6.869143000	-2.813719000	2.484273000
H	6.381398000	-4.227119000	3.430514000
H	5.173200000	-3.243113000	2.588077000
C	11.071804000	3.760487000	0.099174000
C	1.947882000	-1.548611000	-0.514189000
H	1.218945000	-0.766187000	-0.711504000
C	8.716462000	2.240279000	-2.478355000
H	8.610749000	1.150036000	-2.514129000
H	9.357968000	2.542655000	-3.309562000
H	7.715684000	2.649570000	-2.654817000
C	5.790053000	-4.357992000	-2.473755000
H	4.722625000	-4.125065000	-2.562247000
H	6.036442000	-5.091311000	-3.245256000
H	6.327677000	-3.431021000	-2.701540000
C	12.396783000	4.460052000	0.134150000

H	12.276712000	5.547163000	0.058352000
H	13.038891000	4.152443000	-0.695687000
H	12.932878000	4.265011000	1.067150000
C	7.499652000	-8.063421000	0.468476000
H	8.513913000	-8.065206000	0.882160000
H	7.531714000	-8.593014000	-0.486951000
H	6.882255000	-8.652589000	1.154760000
S	-4.426364000	-1.917295000	-0.108705000
S	-2.874339000	3.992971000	-0.015212000
Se	-1.218079000	-2.185892000	-0.285043000
N	-5.938910000	0.368897000	0.001136000
C	-6.259476000	1.687417000	-0.015202000
C	-5.854781000	4.146672000	-0.053134000
C	-7.118520000	-0.298424000	0.018096000
C	-7.196487000	-1.705566000	0.044093000
C	-8.534312000	-2.358229000	0.113164000
C	-7.702860000	1.867493000	-0.014987000
H	-8.217616000	2.820853000	-0.027732000
C	-5.328273000	2.750506000	-0.018573000
C	-6.060073000	-2.530142000	0.022114000
C	-9.121434000	-2.885588000	-1.052060000
C	-1.583626000	-4.847164000	0.005338000
H	-2.054268000	-5.818355000	0.121700000
C	-1.441682000	2.992797000	0.041485000
C	0.246352000	-3.363934000	-0.198675000
C	-6.027542000	-3.940059000	0.105209000
H	-6.943206000	-4.515924000	0.195429000
C	-3.744094000	-3.523293000	-0.047079000
C	-3.933607000	2.578310000	0.011907000

C	-8.242147000	0.618836000	0.007759000
H	-9.288439000	0.337373000	0.012733000
C	-0.205624000	-4.678871000	-0.044515000
H	0.492700000	-5.507873000	0.025387000
C	-4.757300000	-4.488547000	0.070717000
H	-4.554961000	-5.552586000	0.128385000
C	-10.380088000	-3.480980000	-0.965327000
H	-10.836004000	-3.878491000	-1.871587000
C	-2.345832000	-3.677946000	-0.093690000
C	-6.088738000	4.776044000	-1.287808000
C	-9.213199000	-2.434668000	1.342750000
C	-3.157581000	1.404791000	0.066156000
H	-3.628845000	0.433460000	0.105143000
C	-6.111177000	4.830087000	1.151218000
C	-8.415163000	-2.793123000	-2.371675000
H	-7.485250000	-3.373217000	-2.375880000
H	-9.044158000	-3.162910000	-3.184918000
H	-8.130843000	-1.760821000	-2.603128000
C	-6.573393000	6.085554000	-1.296811000
H	-6.753619000	6.572098000	-2.254717000
C	-6.833144000	6.783070000	-0.118376000
C	-10.469669000	-3.040898000	1.384917000
H	-10.992792000	-3.098312000	2.338895000
C	-6.595602000	6.135709000	1.096230000
H	-6.793681000	6.661333000	2.029908000
C	-5.829023000	4.050737000	-2.573794000
H	-6.448064000	3.150555000	-2.658979000
H	-6.037713000	4.686596000	-3.437382000
H	-4.788200000	3.715353000	-2.645214000

C	-11.070463000	-3.572702000	0.244010000
C	-1.797733000	1.636109000	0.082061000
H	-1.055437000	0.843980000	0.130208000
C	-8.599628000	-1.874372000	2.591090000
H	-8.489547000	-0.785449000	2.532636000
H	-9.208267000	-2.103595000	3.469105000
H	-7.593590000	-2.273569000	2.760257000
C	-5.874130000	4.158444000	2.470274000
H	-4.831387000	3.839269000	2.579163000
H	-6.110777000	4.824553000	3.303304000
H	-6.484303000	3.254309000	2.575508000
C	-12.408128000	-4.244595000	0.317384000
H	-12.304375000	-5.330022000	0.431342000
H	-12.993424000	-3.888568000	1.169423000
H	-12.995390000	-4.077294000	-0.590041000
C	-7.356176000	8.187346000	-0.147487000
H	-8.337177000	8.261611000	0.334144000
H	-6.693345000	8.875355000	0.387904000
H	-7.461694000	8.557021000	-1.170492000

15A:

Se	0.000138000	-4.076203000	0.093491000
S	-3.840307000	-1.692663000	0.257900000
S	3.840459000	-1.692519000	0.258962000
N	-6.153625000	-0.000182000	-0.000642000
C	-7.552861000	-3.501319000	-0.095727000
C	-6.532596000	-2.415526000	-0.008137000
C	-6.977463000	-1.080340000	-0.010973000
C	-7.976518000	-4.163238000	1.072615000

C	-5.184401000	-2.803612000	0.072287000
C	-9.487767000	-5.539163000	-0.258938000
C	-1.308192000	-2.764337000	0.416257000
C	-4.710186000	-4.132285000	0.026513000
H	-5.392697000	-4.968338000	-0.084030000
C	-3.338027000	-4.246367000	0.138049000
H	-2.799222000	-5.189632000	0.131535000
C	2.688772000	-3.013340000	0.291741000
C	-8.095249000	-3.854534000	-1.343179000
C	-8.937422000	-5.168105000	0.969869000
H	-9.268224000	-5.672222000	1.877489000
C	-2.688574000	-3.013443000	0.291159000
C	1.308356000	-2.764295000	0.416544000
C	-6.977523000	1.079961000	0.010372000
C	-9.053201000	-4.869239000	-1.401389000
H	-9.470388000	-5.142440000	-2.370028000
C	-0.693664000	-1.551489000	0.739998000
H	-1.282589000	-0.674742000	0.997313000
C	-6.532736000	2.415136000	0.006978000
C	-8.373110000	-0.679287000	-0.009920000
H	-9.221108000	-1.353423000	-0.015850000
C	0.693713000	-1.551472000	0.740165000
H	1.282553000	-0.674712000	0.997627000
C	5.184646000	-2.803376000	0.073526000
C	-7.553083000	3.500880000	0.094607000
C	-8.373119000	0.678811000	0.010606000
H	-9.221120000	1.352908000	0.017384000
C	-7.414576000	-3.783668000	2.410078000
H	-7.539278000	-2.713509000	2.608688000

H	-6.337992000	-3.980786000	2.467997000
H	-7.900951000	-4.337654000	3.216479000
C	-7.976793000	4.162431000	-1.072373000
C	3.338324000	-4.246223000	0.138663000
H	2.799574000	-5.189518000	0.131888000
C	-10.509363000	-6.632707000	-0.342063000
H	-11.334996000	-6.468112000	0.357760000
H	-10.076460000	-7.607265000	-0.090143000
H	-10.933549000	-6.712679000	-1.346107000
C	-7.655468000	-3.154160000	-2.593925000
H	-6.566485000	-3.180036000	-2.710048000
H	-7.935711000	-2.094646000	-2.581518000
H	-8.102509000	-3.608956000	-3.481164000
C	-8.089195000	3.859960000	1.344507000
C	-9.041803000	4.877901000	1.404629000
H	-9.449415000	5.158434000	2.375339000
C	4.710504000	-4.132090000	0.027510000
H	5.393050000	-4.968152000	-0.082922000
C	-9.480925000	5.545045000	0.260661000
C	-8.933892000	5.172405000	-0.967598000
H	-9.261563000	5.681000000	-1.873731000
C	-7.416281000	3.784025000	-2.410745000
H	-7.542049000	2.714163000	-2.610238000
H	-6.339625000	3.980453000	-2.469768000
H	-7.903210000	4.339025000	-3.216116000
C	-7.641245000	3.164590000	2.595153000
H	-6.551930000	3.195761000	2.707140000
H	-7.916479000	2.103766000	2.585944000
H	-8.087067000	3.619216000	3.483081000

C	-10.529694000	6.612296000	0.345340000
H	-10.422301000	7.349901000	-0.454770000
H	-10.492243000	7.141895000	1.301347000
H	-11.537207000	6.189570000	0.255362000
Se	-0.000153000	4.076131000	-0.094894000
S	3.840272000	1.692598000	-0.258631000
S	-3.840415000	1.692391000	-0.259081000
N	6.153603000	0.000142000	0.000751000
C	7.552664000	3.501360000	0.096169000
C	6.532481000	2.415505000	0.008250000
C	6.977390000	1.080362000	0.011544000
C	7.977712000	4.162295000	-1.072217000
C	5.184316000	2.803583000	-0.072893000
C	9.487393000	5.539308000	0.259985000
C	1.308164000	2.764216000	-0.417457000
C	4.710126000	4.132275000	-0.027777000
H	5.392622000	4.968356000	0.082618000
C	3.337983000	4.246335000	-0.139633000
H	2.799187000	5.189607000	-0.133559000
C	-2.688782000	3.013230000	-0.292728000
C	8.093550000	3.855611000	1.343971000
C	8.938507000	5.167235000	-0.969169000
H	9.270367000	5.670620000	-1.876809000
C	2.688552000	3.013367000	-0.292364000
C	-1.308363000	2.764148000	-0.417614000
C	6.977549000	-1.079943000	-0.009651000
C	9.051456000	4.870344000	1.402477000
H	9.467504000	5.144338000	2.371381000
C	0.693648000	1.551310000	-0.740941000

H	1.282572000	0.674530000	-0.998140000
C	6.532832000	-2.415181000	-0.006618000
C	8.373047000	0.679366000	0.011582000
H	9.221012000	1.353538000	0.018168000
C	-0.693743000	1.551270000	-0.741010000
H	-1.282591000	0.674456000	-0.998271000
C	-5.184580000	2.803311000	-0.073873000
C	7.553288000	-3.500820000	-0.093884000
C	8.373130000	-0.678733000	-0.008926000
H	9.221169000	-1.352787000	-0.014996000
C	7.417337000	3.781574000	-2.410007000
H	7.543478000	2.711485000	-2.608108000
H	6.340573000	3.977386000	-2.468884000
H	7.903750000	4.335804000	-3.216216000
C	7.973705000	-4.165152000	1.072666000
C	-3.338327000	4.246172000	-0.140230000
H	-2.799607000	5.189486000	-0.134142000
C	10.508841000	6.632966000	0.343430000
H	11.334876000	6.468302000	-0.355896000
H	10.075974000	7.607427000	0.091062000
H	10.932448000	6.713219000	1.347697000
C	7.652304000	3.156239000	2.594762000
H	6.563161000	3.181858000	2.709398000
H	7.932927000	2.096809000	2.583731000
H	8.098002000	3.611989000	3.482188000
C	8.092895000	-3.857022000	-1.343186000
C	9.045506000	-4.874898000	-1.403079000
H	9.455759000	-5.153188000	-2.373325000
C	-4.710495000	4.132063000	-0.028769000

H	-5.393056000	4.968164000	0.081238000
C	9.481382000	-5.544829000	-0.259434000
C	8.931012000	-5.175024000	0.968123000
H	9.256263000	-5.685668000	1.873972000
C	7.409485000	-3.790199000	2.410465000
H	7.528795000	-2.719711000	2.610407000
H	6.333899000	-3.992929000	2.468234000
H	7.898544000	-4.342731000	3.216247000
C	7.648426000	-3.158704000	-2.593430000
H	6.559422000	-3.189632000	-2.708497000
H	7.923586000	-2.097894000	-2.580883000
H	8.096734000	-3.611227000	-3.481181000
C	10.530313000	-6.611932000	-0.343899000
H	10.489425000	-7.145430000	-1.297622000
H	11.537975000	-6.188342000	-0.259803000
H	10.426399000	-7.346311000	0.459599000

15B:

S	4.492642000	-1.929906000	-0.074071000
S	2.916985000	3.922123000	0.013912000
Se	1.273206000	-2.195155000	-0.167789000
N	6.016243000	0.345245000	0.059299000
C	6.335224000	1.663354000	0.053710000
C	5.911889000	4.120484000	-0.033754000
C	7.195479000	-0.322659000	0.043396000
C	7.268225000	-1.729808000	0.029964000
C	8.604486000	-2.389453000	0.046349000
C	7.778112000	1.844173000	0.032104000
H	8.292412000	2.797933000	0.026006000

C	5.398454000	2.723360000	0.063361000
C	6.127850000	-2.549894000	0.006616000
C	9.171002000	-2.857398000	-1.153777000
C	1.650685000	-4.865106000	-0.008024000
H	2.125539000	-5.839250000	0.053928000
C	1.506027000	2.897740000	0.159081000
C	-0.186919000	-3.381101000	-0.117228000
C	6.093403000	-3.961333000	0.046710000
H	7.009268000	-4.541581000	0.099053000
C	3.807905000	-3.536234000	-0.042143000
C	4.008343000	2.536167000	0.139918000
C	8.318417000	0.595159000	0.028555000
H	9.364850000	0.314621000	0.011226000
C	0.271371000	-4.699911000	-0.031789000
H	-0.423093000	-5.534142000	0.007501000
C	4.821212000	-4.506262000	0.022943000
H	4.617823000	-5.571253000	0.052536000
C	10.427965000	-3.461531000	-1.117629000
H	10.867917000	-3.813633000	-2.050157000
C	2.408456000	-3.690393000	-0.064983000
C	6.235597000	4.660866000	-1.291636000
C	9.301509000	-2.532194000	1.259564000
C	3.259148000	1.354537000	0.301682000
H	3.750747000	0.400441000	0.423720000
C	6.074730000	4.892277000	1.132094000
C	8.444256000	-2.694103000	-2.455218000
H	7.504941000	-3.258632000	-2.468953000
H	9.053285000	-3.036219000	-3.295326000
H	8.173279000	-1.647965000	-2.635061000

C	6.708723000	5.971601000	-1.361556000
H	6.957498000	6.388230000	-2.336975000
C	6.872771000	6.757752000	-0.221057000
C	10.555380000	-3.145005000	1.250886000
H	11.092870000	-3.253737000	2.192305000
C	6.554792000	6.196549000	1.016512000
H	6.687712000	6.788762000	1.921299000
C	6.082214000	3.840131000	-2.536869000
H	6.767240000	2.984432000	-2.540653000
H	6.283354000	4.434990000	-3.430978000
H	5.072659000	3.424262000	-2.625327000
C	11.135998000	-3.619171000	0.074526000
C	1.896850000	1.557281000	0.308953000
H	1.172725000	0.755205000	0.432705000
C	8.709080000	-2.033256000	2.543558000
H	8.589510000	-0.943881000	2.535671000
H	9.336686000	-2.296145000	3.398454000
H	7.709733000	-2.448279000	2.714723000
C	5.757127000	4.314120000	2.478322000
H	4.701838000	4.028589000	2.556534000
H	5.971108000	5.028089000	3.277126000
H	6.336917000	3.404675000	2.671340000
C	12.470975000	-4.300134000	0.092561000
H	12.363375000	-5.388208000	0.172914000
H	13.078978000	-3.976332000	0.941593000
H	13.037007000	-4.105418000	-0.822774000
C	7.354266000	8.173287000	-0.322355000
H	7.969206000	8.454347000	0.537359000
H	6.515410000	8.878252000	-0.355479000

H	7.944278000	8.336163000	-1.228295000
S	-4.492665000	1.929710000	0.074193000
S	-2.916994000	-3.921843000	-0.014335000
Se	-1.273154000	2.195207000	0.167139000
N	-6.016344000	-0.345308000	-0.059614000
C	-6.335329000	-1.663416000	-0.054080000
C	-5.911788000	-4.120493000	0.033555000
C	-7.195587000	0.322556000	-0.043444000
C	-7.268301000	1.729692000	-0.029752000
C	-8.604544000	2.389376000	-0.045864000
C	-7.778204000	-1.844271000	-0.032202000
H	-8.292478000	-2.798046000	-0.026068000
C	-5.398507000	-2.723338000	-0.063868000
C	-6.127899000	2.549721000	-0.006349000
C	-9.170896000	2.857121000	1.154416000
C	-1.650930000	4.865162000	0.008151000
H	-2.125900000	5.839269000	-0.053452000
C	-1.506096000	-2.897434000	-0.159847000
C	0.186836000	3.381346000	0.116718000
C	-6.093484000	3.961161000	-0.046162000
H	-7.009365000	4.541397000	-0.098304000
C	-3.807987000	3.536079000	0.042276000
C	-4.008427000	-2.535996000	-0.140716000
C	-8.318525000	-0.595265000	-0.028651000
H	-9.364959000	-0.314738000	-0.011222000
C	-0.271603000	4.700123000	0.031686000
H	0.422760000	5.534441000	-0.007468000
C	-4.821315000	4.506100000	-0.022474000
H	-4.617944000	5.571096000	-0.051934000

C	-10.427838000	3.461308000	1.118535000
H	-10.867660000	3.813258000	2.051181000
C	-2.408561000	3.690351000	0.064892000
C	-6.235486000	-4.660615000	1.291562000
C	-9.301712000	2.532361000	-1.258965000
C	-3.259330000	-1.354369000	-0.303044000
H	-3.750979000	-0.400319000	-0.425490000
C	-6.074524000	-4.892574000	-1.132116000
C	-8.443998000	2.693577000	2.455742000
H	-7.504696000	3.258121000	2.469489000
H	-9.052940000	3.035511000	3.295985000
H	-8.172981000	1.647410000	2.635345000
C	-6.708451000	-5.971388000	1.361775000
H	-6.957200000	-6.387819000	2.337287000
C	-6.872385000	-6.757824000	0.221450000
C	-10.555557000	3.145219000	-1.250020000
H	-11.093158000	3.254144000	-2.191352000
C	-6.554455000	-6.196872000	-1.016240000
H	-6.687314000	-6.789305000	-1.920891000
C	-6.082222000	-3.839574000	2.536609000
H	-6.767338000	-2.983947000	2.540176000
H	-6.283320000	-4.434250000	3.430849000
H	-5.072711000	-3.423578000	2.624995000
C	-11.136011000	3.619196000	-0.073504000
C	-1.896998000	-1.557043000	-0.310275000
H	-1.172917000	-0.754974000	-0.434364000
C	-8.709471000	2.033631000	-2.543127000
H	-8.590042000	0.944240000	-2.535502000
H	-9.337129000	2.296789000	-3.397902000

H	-7.710091000	2.448562000	-2.714301000
C	-5.756992000	-4.314684000	-2.478475000
H	-4.701717000	-4.029118000	-2.556775000
H	-5.970957000	-5.028834000	-3.277123000
H	-6.336834000	-3.405310000	-2.671671000
C	-12.470959000	4.300222000	-0.091252000
H	-12.363323000	5.388299000	-0.171508000
H	-13.079113000	3.976539000	-0.940221000
H	-13.036853000	4.105435000	0.824153000
C	-7.353702000	-8.173394000	0.323088000
H	-7.967709000	-8.455079000	-0.537084000
H	-6.514734000	-8.878167000	0.357504000
H	-7.944598000	-8.335864000	1.228528000

10A.2H^{+}:

S	3.815164000	1.829511000	0.675963000
S	3.814200000	-1.829318000	-0.675492000
S	-0.000448000	-3.961217000	-0.244757000
N	6.154090000	0.000008000	-0.000374000
C	8.145435000	-3.713721000	1.373572000
C	7.497819000	-3.540013000	0.131584000
C	7.799097000	-4.385079000	-0.958406000
C	6.482456000	-2.468245000	-0.034236000
C	9.419775000	-5.570881000	0.430412000
C	5.134821000	2.867284000	0.159747000
C	6.935509000	1.135904000	0.023775000
C	6.934998000	-1.136270000	-0.023609000
C	6.483343000	2.468027000	0.034115000
C	2.626227000	3.084131000	0.423849000

C	9.088217000	-4.732491000	1.497400000
H	9.572910000	-4.881795000	2.460448000
C	7.498950000	3.539508000	-0.131449000
C	4.628936000	4.152127000	-0.117789000
H	5.283906000	4.952840000	-0.443200000
C	8.288380000	0.684930000	0.014731000
H	9.142984000	1.346993000	0.034694000
C	8.761694000	-5.377631000	-0.786463000
H	9.009017000	-6.017854000	-1.631243000
C	-1.254936000	-2.795576000	-0.593584000
C	7.798663000	4.385880000	0.957917000
C	8.288074000	-0.685892000	-0.012666000
H	9.142390000	-1.348372000	-0.031125000
C	2.625290000	-3.084121000	-0.424129000
C	5.133955000	-2.867406000	-0.160225000
C	3.256048000	4.267580000	0.018040000
H	2.703572000	5.178642000	-0.185034000
C	7.812187000	-2.859196000	2.562138000
H	8.261124000	-1.861451000	2.493994000
H	8.181308000	-3.313207000	3.483685000
H	6.732756000	-2.710017000	2.671601000
C	3.255188000	-4.267794000	-0.019096000
H	2.702750000	-5.179000000	0.183433000
C	4.628093000	-4.152434000	0.116584000
H	5.283113000	-4.953368000	0.441351000
C	7.131263000	-4.217639000	-2.292344000
H	6.105761000	-4.605437000	-2.291887000
H	7.676207000	-4.753890000	-3.071339000
H	7.067180000	-3.166498000	-2.592035000

C	1.253994000	-2.795562000	-0.593714000
C	7.128927000	4.220385000	2.291153000
H	6.103823000	4.609212000	2.289053000
H	7.673428000	4.756874000	3.070294000
H	7.063424000	3.169612000	2.591806000
C	0.690915000	-1.574190000	-0.987888000
H	1.296863000	-0.715386000	-1.260602000
C	8.761885000	5.377975000	0.786254000
H	9.008056000	6.019178000	1.630613000
C	-0.691908000	-1.574202000	-0.987835000
H	-1.297897000	-0.715400000	-1.260469000
C	8.148763000	3.711482000	-1.372652000
C	9.091974000	4.729765000	-1.496223000
H	9.578238000	4.877740000	-2.458694000
C	7.817452000	2.855504000	-2.560735000
H	8.269293000	1.859085000	-2.492286000
H	8.185072000	3.310131000	-3.482586000
H	6.738442000	2.703201000	-2.669828000
C	10.464301000	-6.630860000	0.580155000
H	10.525709000	-6.997922000	1.607183000
H	11.455153000	-6.241562000	0.321138000
H	10.276121000	-7.482130000	-0.078137000
C	9.422015000	5.569479000	-0.429687000
C	10.467088000	6.628891000	-0.579549000
H	10.290374000	7.471633000	0.092638000
H	10.515050000	7.009623000	-1.602382000
H	11.460549000	6.233561000	-0.340449000
S	-3.815166000	-1.829501000	-0.675964000
S	-3.814183000	1.829307000	0.675411000

S	0.000455000	3.961241000	0.244772000
N	-6.154084000	-0.000002000	0.000411000
C	-8.145684000	3.713468000	-1.373477000
C	-7.497820000	3.540011000	-0.131590000
C	-7.798879000	4.385284000	0.958295000
C	-6.482448000	2.468251000	0.034237000
C	-9.419821000	5.570827000	-0.430438000
C	-5.134812000	-2.867263000	-0.159693000
C	-6.935507000	-1.135894000	-0.023767000
C	-6.934990000	1.136278000	0.023609000
C	-6.483338000	-2.468014000	-0.034082000
C	-2.626220000	-3.084104000	-0.423807000
C	-9.088489000	4.732215000	-1.497323000
H	-9.573390000	4.881314000	-2.460298000
C	-7.498941000	-3.539496000	0.131477000
C	-4.628918000	-4.152092000	0.117889000
H	-5.283883000	-4.952796000	0.443331000
C	-8.288377000	-0.684916000	-0.014791000
H	-9.142982000	-1.346977000	-0.034804000
C	-8.761497000	5.377817000	0.786345000
H	-9.008638000	6.018216000	1.631043000
C	1.254941000	2.795600000	0.593601000
C	-7.798549000	-4.385952000	-0.957858000
C	-8.288068000	0.685907000	0.012585000
H	-9.142382000	1.348391000	0.030975000
C	-2.625283000	3.084130000	0.424102000
C	-5.133948000	2.867413000	0.160208000
C	-3.256030000	-4.267541000	-0.017946000
H	-2.703547000	-5.178592000	0.185159000

C	-7.812733000	2.858622000	-2.561894000
H	-8.261710000	1.860918000	-2.493379000
H	-8.182028000	3.312407000	-3.483482000
H	-6.733334000	2.709351000	-2.671546000
C	-3.255188000	4.267811000	0.019101000
H	-2.702755000	5.179027000	-0.183398000
C	-4.628094000	4.152452000	-0.116572000
H	-5.283122000	4.953395000	-0.441299000
C	-7.130746000	4.218109000	2.292116000
H	-6.105229000	4.605865000	2.291337000
H	-7.675488000	4.754550000	3.071121000
H	-7.066638000	3.167029000	2.592020000
C	-1.253988000	2.795576000	0.593692000
C	-7.128690000	-4.220533000	-2.291041000
H	-6.103536000	-4.609227000	-2.288785000
H	-7.673041000	-4.757182000	-3.070177000
H	-7.063289000	-3.169786000	-2.591809000
C	-0.690909000	1.574197000	0.987845000
H	-1.296858000	0.715386000	1.260534000
C	-8.761766000	-5.378046000	-0.786208000
H	-9.007851000	-6.019316000	-1.630543000
C	0.691912000	1.574215000	0.987814000
H	1.297900000	0.715410000	1.260441000
C	-8.148856000	-3.711390000	1.372631000
C	-9.092065000	-4.729682000	1.496194000
H	-9.578411000	-4.877595000	2.458633000
C	-7.817678000	-2.855309000	2.560677000
H	-8.269605000	-1.858935000	2.492136000
H	-8.185306000	-3.309907000	3.482539000

H	-6.738687000	-2.702902000	2.669816000
C	-10.464370000	6.630784000	-0.580182000
H	-10.525989000	6.997634000	-1.607274000
H	-11.455171000	6.241549000	-0.320877000
H	-10.276045000	7.482189000	0.077894000
C	-9.421999000	-5.569475000	0.429694000
C	-10.467063000	-6.628901000	0.579521000
H	-10.289738000	-7.472099000	-0.091938000
H	-10.515757000	-7.008916000	1.602583000
H	-11.460385000	-6.233865000	0.339358000
H	5.146531000	0.000388000	-0.001895000
H	-5.146525000	-0.000396000	0.002062000

10B.2H+:

S	4.273081000	2.118787000	-0.189486000
S	3.057144000	-3.953631000	0.183114000
S	1.085099000	2.532758000	0.076699000
N	5.981758000	-0.310636000	0.004289000
H	5.146528000	-0.068389000	0.517527000
C	6.356005000	-1.625670000	-0.197809000
C	6.087567000	-4.083780000	-0.089783000
C	7.079385000	0.523226000	-0.108167000
C	7.072455000	1.926930000	-0.011638000
C	8.390217000	2.613960000	0.005626000
C	7.749164000	-1.614362000	-0.475680000
H	8.320915000	-2.496166000	-0.731114000
C	5.487066000	-2.731245000	-0.174900000
C	5.910906000	2.720297000	0.042031000
C	9.213318000	2.511080000	1.147150000

C	1.460102000	5.091582000	-0.079504000
H	1.947304000	6.057486000	-0.142522000
C	1.612397000	-3.030098000	-0.126742000
C	-0.314910000	3.578831000	0.023337000
C	5.862695000	4.117684000	0.222574000
H	6.768878000	4.690003000	0.388257000
C	3.578137000	3.704601000	0.004186000
C	4.084901000	-2.587613000	-0.221510000
C	8.185817000	-0.311273000	-0.428623000
H	9.178594000	0.061945000	-0.640345000
C	0.083880000	4.916852000	-0.076592000
H	-0.629930000	5.732473000	-0.115191000
C	4.590121000	4.658212000	0.188684000
H	4.376209000	5.710714000	0.333087000
C	10.448114000	3.156274000	1.139747000
H	11.078672000	3.088785000	2.024371000
C	2.178011000	3.890773000	-0.007796000
C	6.723999000	-4.491845000	1.102497000
C	8.817007000	3.344792000	-1.123062000
C	3.291675000	-1.479684000	-0.585627000
H	3.711823000	-0.559535000	-0.973651000
C	6.024816000	-4.951474000	-1.203296000
C	8.775038000	1.748605000	2.363727000
H	7.751256000	2.001571000	2.659809000
H	9.427603000	1.959341000	3.212902000
H	8.796592000	0.664254000	2.201524000
C	7.266244000	-5.773612000	1.164150000
H	7.734932000	-6.099375000	2.090830000
C	7.221389000	-6.650656000	0.078041000

C	10.067922000	3.958963000	-1.084774000
H	10.409548000	4.503256000	-1.963263000
C	6.599163000	-6.215719000	-1.094816000
H	6.567962000	-6.879363000	-1.956968000
C	6.790185000	-3.592857000	2.302958000
H	7.500291000	-2.769272000	2.161897000
H	7.110617000	-4.145707000	3.187834000
H	5.820102000	-3.136585000	2.529121000
C	10.897323000	3.885727000	0.036305000
C	1.934478000	-1.721650000	-0.520061000
H	1.176908000	-0.997885000	-0.804657000
C	7.978453000	3.436999000	-2.364690000
H	7.537181000	2.472075000	-2.635740000
H	8.573897000	3.781317000	-3.212242000
H	7.144968000	4.140065000	-2.250820000
C	5.406728000	-4.517844000	-2.500029000
H	4.316674000	-4.424801000	-2.431130000
H	5.619726000	-5.236839000	-3.293055000
H	5.783992000	-3.542307000	-2.825210000
C	12.219762000	4.584393000	0.063473000
H	12.121847000	5.590441000	0.486189000
H	12.638744000	4.699014000	-0.938728000
H	12.947255000	4.051461000	0.680221000
C	7.847253000	-8.006829000	0.157761000
H	8.885435000	-7.978539000	-0.191227000
H	7.322918000	-8.733113000	-0.467733000
H	7.865942000	-8.385697000	1.182066000
S	-4.273102000	-2.118823000	0.189576000
S	-3.057158000	3.953626000	-0.182973000

S	-1.085115000	-2.532775000	-0.076509000
N	-5.981785000	0.310648000	-0.004269000
H	-5.146485000	0.068404000	-0.517387000
C	-6.356024000	1.625666000	0.197905000
C	-6.087571000	4.083788000	0.089959000
C	-7.079394000	-0.523228000	0.108159000
C	-7.072465000	-1.926933000	0.011563000
C	-8.390233000	-2.613944000	-0.005845000
C	-7.749189000	1.614343000	0.475751000
H	-8.320948000	2.496135000	0.731207000
C	-5.487085000	2.731243000	0.175032000
C	-5.910922000	-2.720309000	-0.042049000
C	-9.213221000	-2.510993000	-1.147450000
C	-1.460114000	-5.091614000	0.079487000
H	-1.947314000	-6.057525000	0.142423000
C	-1.612413000	3.030089000	0.126879000
C	0.314896000	-3.578851000	-0.023226000
C	-5.862707000	-4.117695000	-0.222621000
H	-6.768885000	-4.690005000	-0.388370000
C	-3.578153000	-3.704629000	-0.004140000
C	-4.084921000	2.587611000	0.221657000
C	-8.185834000	0.311254000	0.428636000
H	-9.178617000	-0.061975000	0.640313000
C	-0.083893000	-4.916881000	0.076602000
H	0.629920000	-5.732502000	0.115140000
C	-4.590139000	-4.658231000	-0.188697000
H	-4.376226000	-5.710730000	-0.333114000
C	-10.448009000	-3.156202000	-1.140216000
H	-11.078476000	-3.088664000	-2.024901000

C	-2.178028000	-3.890801000	0.007864000
C	-6.724019000	4.491899000	-1.102303000
C	-8.817135000	-3.344859000	1.122746000
C	-3.291695000	1.479685000	0.585785000
H	-3.711838000	0.559544000	0.973835000
C	-6.024750000	4.951460000	1.203478000
C	-8.774831000	-1.748452000	-2.363947000
H	-7.751004000	-2.001362000	-2.659921000
H	-9.427289000	-1.959190000	-3.213203000
H	-8.796456000	-0.664107000	-2.201714000
C	-7.266202000	5.773692000	-1.163919000
H	-7.734899000	6.099489000	-2.090582000
C	-7.221278000	6.650721000	-0.077797000
C	-10.068041000	-3.959042000	1.084287000
H	-10.409752000	-4.503394000	1.962706000
C	-6.599042000	6.215738000	1.095034000
H	-6.567788000	6.879368000	1.957195000
C	-6.790280000	3.592945000	-2.302784000
H	-7.500422000	2.769389000	-2.161745000
H	-7.110702000	4.145842000	-3.187636000
H	-5.820230000	3.136630000	-2.528984000
C	-10.897325000	-3.885738000	-0.036870000
C	-1.934498000	1.721648000	0.520219000
H	-1.176930000	0.997884000	0.804825000
C	-7.978706000	-3.437167000	2.364452000
H	-7.537164000	-2.472348000	2.635429000
H	-8.574317000	-3.781218000	3.211997000
H	-7.145428000	-4.140502000	2.250725000
C	-5.406634000	4.517802000	2.500188000

H	-4.316572000	4.424846000	2.431280000
H	-5.619684000	5.236742000	3.293252000
H	-5.783822000	3.542227000	2.825327000
C	-12.219756000	-4.584412000	-0.064229000
H	-12.121806000	-5.590391000	-0.487103000
H	-12.638798000	-4.699197000	0.937928000
H	-12.947216000	-4.051387000	-0.680936000
C	-7.847084000	8.006921000	-0.157498000
H	-8.885497000	7.978523000	0.190796000
H	-7.323148000	8.733013000	0.468549000
H	-7.865120000	8.386099000	-1.181702000

13A.2H^{+}:

S	0.002121000	4.072687000	0.343935000
S	3.776679000	1.906711000	-0.158806000
S	-3.773811000	1.918070000	-0.182343000
N	6.191359000	0.122792000	-0.008242000
C	7.519423000	3.671149000	-0.061733000
C	6.496097000	2.595257000	-0.041004000
C	6.945766000	1.269155000	-0.179840000
C	8.425116000	3.802068000	1.014526000
C	5.147722000	2.972196000	0.111481000
C	9.465904000	5.700138000	-0.114805000
C	1.254657000	2.856702000	0.254258000
C	4.678973000	4.256886000	0.453217000
H	5.369629000	5.065872000	0.664735000
C	3.301139000	4.361889000	0.521959000
H	2.775227000	5.270297000	0.795034000
C	-2.629442000	3.165201000	0.247053000

C	7.583395000	4.550649000	-1.162649000
C	9.374270000	4.819611000	0.967430000
H	10.061645000	4.932353000	1.803980000
C	2.632153000	3.161426000	0.248100000
C	-1.252208000	2.858957000	0.251060000
C	6.979999000	-0.998960000	-0.184016000
C	8.564397000	5.541974000	-1.168085000
H	8.631117000	6.206482000	-2.027439000
C	0.690493000	1.577222000	0.170719000
H	1.297292000	0.676906000	0.156903000
C	6.551220000	-2.330405000	-0.039537000
C	8.253103000	0.839451000	-0.540441000
H	9.056939000	1.514254000	-0.801227000
C	-0.690187000	1.578473000	0.167971000
H	-1.298820000	0.679427000	0.152649000
C	-5.145199000	2.975590000	0.117628000
C	7.590220000	-3.376862000	0.085697000
C	8.275435000	-0.534198000	-0.536217000
H	9.100187000	-1.184936000	-0.792608000
C	8.360850000	2.895898000	2.210039000
H	8.766672000	1.899618000	1.996748000
H	7.332935000	2.747942000	2.557951000
H	8.937809000	3.305516000	3.041226000
C	7.706794000	-4.374964000	-0.911295000
C	-3.298443000	4.359689000	0.546167000
H	-2.772306000	5.262682000	0.836209000
C	10.495380000	6.785315000	-0.131307000
H	11.489428000	6.399471000	0.111992000
H	10.269103000	7.554695000	0.614416000

H	10.551911000	7.277221000	-1.104246000
C	6.653607000	4.419251000	-2.334008000
H	5.657256000	4.822258000	-2.117459000
H	6.510005000	3.375516000	-2.631958000
H	7.037991000	4.963469000	-3.198660000
C	8.469222000	-3.367344000	1.192473000
C	9.441516000	-4.361751000	1.282269000
H	10.105443000	-4.367794000	2.144593000
C	-4.676445000	4.253763000	0.483498000
H	-5.367148000	5.056588000	0.717301000
C	9.584744000	-5.350816000	0.307693000
C	8.709436000	-5.331617000	-0.782452000
H	8.819156000	-6.081312000	-1.563929000
C	6.819842000	-4.397761000	-2.123091000
H	6.613815000	-3.393381000	-2.505771000
H	5.847579000	-4.859982000	-1.916276000
H	7.280999000	-4.973605000	-2.927764000
C	8.358930000	-2.349404000	2.291886000
H	7.318405000	-2.104222000	2.527051000
H	8.855806000	-1.406453000	2.034515000
H	8.829269000	-2.716509000	3.206264000
C	10.631628000	-6.411471000	0.428865000
H	10.186413000	-7.372693000	0.707610000
H	11.375836000	-6.164296000	1.188408000
H	11.151848000	-6.572606000	-0.519221000
Se	0.000997000	-3.941066000	0.323630000
S	-3.831398000	-1.745986000	-0.614574000
S	3.846608000	-1.747858000	-0.565572000
N	-6.181081000	0.125233000	-0.056033000

C	-7.584158000	-3.372643000	-0.005112000
C	-6.542209000	-2.327656000	-0.117908000
C	-6.968840000	-0.994294000	-0.249966000
C	-7.696378000	-4.365995000	-1.006250000
C	-5.188284000	-2.725189000	-0.065540000
C	-9.578752000	-5.348155000	0.200787000
C	-1.295816000	-2.798488000	-0.403833000
C	-4.716165000	-3.974325000	0.376892000
H	-5.397987000	-4.730030000	0.751107000
C	-3.342304000	-4.118589000	0.307796000
H	-2.810310000	-5.013743000	0.614540000
C	2.690341000	-3.000172000	-0.176718000
C	-8.465097000	-3.370705000	1.101159000
C	-8.698559000	-5.325081000	-0.884218000
H	-8.801170000	-6.075501000	-1.665903000
C	-2.680510000	-3.000549000	-0.216483000
C	1.308499000	-2.798606000	-0.384427000
C	-6.938345000	1.272939000	-0.202420000
C	-9.435779000	-4.366282000	1.183887000
H	-10.096605000	-4.381823000	2.048520000
C	-0.682269000	-1.745478000	-1.089816000
H	-1.266395000	-0.999517000	-1.622586000
C	-6.493192000	2.597051000	-0.030166000
C	-8.264732000	-0.524877000	-0.594079000
H	-9.088649000	-1.171933000	-0.862073000
C	0.705171000	-1.745780000	-1.079821000
H	1.297175000	-1.000062000	-1.604222000
C	5.196131000	-2.725089000	0.004855000
C	-7.527309000	3.661774000	0.012061000

C	-8.245227000	0.848635000	-0.570034000
H	-9.050804000	1.527242000	-0.815050000
C	-6.801274000	-4.387473000	-2.211976000
H	-6.583958000	-3.382215000	-2.585762000
H	-5.834600000	-4.859741000	-2.001543000
H	-7.261509000	-4.953533000	-3.024111000
C	-8.410768000	3.730824000	1.110939000
C	3.344769000	-4.115641000	0.362186000
H	2.808552000	-5.009046000	0.666670000
C	-10.656370000	-6.380421000	0.293364000
H	-11.551885000	-6.057989000	-0.249452000
H	-10.346207000	-7.331333000	-0.146303000
H	-10.957824000	-6.561527000	1.327380000
C	-8.352339000	-2.363381000	2.210289000
H	-7.311199000	-2.124264000	2.449270000
H	-8.845251000	-1.416049000	1.961424000
H	-8.825297000	-2.737482000	3.120469000
C	-7.628000000	4.588701000	-1.047918000
C	-8.622761000	5.562780000	-0.989084000
H	-8.718899000	6.261818000	-1.817852000
C	4.717668000	-3.971374000	0.448534000
H	5.394363000	-4.725615000	0.834787000
C	-9.503802000	5.660665000	0.090610000
C	-9.376327000	4.735372000	1.128867000
H	-10.048501000	4.799720000	1.982544000
C	-8.312292000	2.774143000	2.264368000
H	-8.730078000	1.789277000	2.022448000
H	-7.274908000	2.607982000	2.573486000
H	-8.861126000	3.150304000	3.129779000

C	-6.721447000	4.520123000	-2.242461000
H	-5.724699000	4.923146000	-2.027906000
H	-6.573941000	3.492253000	-2.589670000
H	-7.128391000	5.098512000	-3.073951000
C	-10.538698000	6.739422000	0.143286000
H	-10.129253000	7.652865000	0.588771000
H	-10.896564000	7.006437000	-0.853842000
H	-11.399124000	6.447213000	0.749390000
H	-5.310234000	0.094094000	0.457088000
H	5.320332000	0.098620000	0.504841000

13B.2H^{+}:

S	4.332891000	-2.015202000	0.172480000
S	3.007778000	3.983828000	-0.208776000
Se	1.097846000	-2.223703000	-0.130938000
N	6.006433000	0.382604000	-0.064271000
H	5.200519000	0.130571000	-0.617837000
C	6.345383000	1.702269000	0.165628000
C	6.019813000	4.155497000	0.122836000
C	7.115829000	-0.431667000	0.078396000
C	7.125000000	-1.837264000	-0.004199000
C	8.444613000	-2.517441000	0.008258000
C	7.730150000	1.715676000	0.484193000
H	8.275595000	2.605130000	0.769097000
C	5.449302000	2.786534000	0.145248000
C	5.963644000	-2.631311000	-0.055268000
C	9.297465000	-2.403793000	-1.110832000
C	1.452501000	-4.894055000	0.097351000
H	1.918205000	-5.869809000	0.184949000

C	1.570151000	3.021686000	0.009864000
C	-0.366370000	-3.396088000	-0.057247000
C	5.899419000	-4.030895000	-0.215661000
H	6.799080000	-4.615586000	-0.374855000
C	3.617937000	-3.591990000	-0.002356000
C	4.049527000	2.613707000	0.145216000
C	8.195367000	0.421671000	0.436286000
H	9.188134000	0.066471000	0.677453000
C	0.074856000	-4.715525000	0.080514000
H	-0.625146000	-5.543789000	0.131545000
C	4.620358000	-4.558968000	-0.171770000
H	4.397277000	-5.611497000	-0.301901000
C	10.533904000	-3.044509000	-1.075438000
H	11.186622000	-2.969721000	-1.943220000
C	2.213764000	-3.724302000	0.006887000
C	6.658411000	4.626486000	-1.044497000
C	8.846096000	-3.252836000	1.144008000
C	3.264249000	1.474438000	0.420944000
H	3.686061000	0.541176000	0.772024000
C	5.926470000	4.974139000	1.270121000
C	8.889023000	-1.636069000	-2.334520000
H	7.869387000	-1.880179000	-2.651208000
H	9.556653000	-1.850917000	-3.170880000
H	8.916691000	-0.552290000	-2.170322000
C	7.171390000	5.921744000	-1.047975000
H	7.642688000	6.295304000	-1.955129000
C	7.094668000	6.752328000	0.072372000
C	10.100199000	-3.861144000	1.134271000
H	10.422291000	-4.407708000	2.018705000

C	6.471584000	6.255146000	1.219486000
H	6.415874000	6.882008000	2.107572000
C	6.759862000	3.776379000	-2.277397000
H	7.479127000	2.957941000	-2.154180000
H	7.088445000	4.367348000	-3.134189000
H	5.800873000	3.315712000	-2.539308000
C	10.957550000	-3.778754000	0.034953000
C	1.905229000	1.696365000	0.329220000
H	1.157589000	0.933838000	0.527979000
C	7.980478000	-3.353698000	2.366554000
H	7.505130000	-2.399068000	2.615002000
H	8.565746000	-3.670396000	3.231856000
H	7.171001000	-4.082660000	2.243178000
C	5.304630000	4.473518000	2.540645000
H	4.217917000	4.357552000	2.454942000
H	5.491079000	5.164583000	3.364620000
H	5.701801000	3.494932000	2.831180000
C	12.282783000	-4.472355000	0.037923000
H	12.196426000	-5.481725000	-0.379418000
H	12.682671000	-4.578850000	1.048823000
H	13.020057000	-3.940959000	-0.568354000
C	7.689292000	8.124788000	0.054356000
H	8.733652000	8.102153000	0.384810000
H	7.159251000	8.805551000	0.724506000
H	7.682950000	8.556527000	-0.949060000
S	-4.336236000	2.128622000	-0.259479000
S	-3.100145000	-3.855022000	0.185387000
S	-1.131253000	2.520627000	-0.044881000
N	-6.059192000	-0.285951000	-0.023139000

H	-5.210757000	-0.046049000	0.469330000
C	-6.440856000	-1.599320000	-0.217790000
C	-6.154901000	-4.055640000	-0.070063000
C	-7.152773000	0.552629000	-0.121953000
C	-7.134114000	1.956305000	-0.019766000
C	-8.445716000	2.651995000	0.031639000
C	-7.837188000	-1.582934000	-0.476687000
H	-8.416664000	-2.462430000	-0.722354000
C	-5.568091000	-2.702903000	-0.196172000
C	-5.964808000	2.739201000	0.017610000
C	-9.250770000	2.532556000	1.184770000
C	-1.503499000	5.084164000	-0.117471000
H	-1.989476000	6.052296000	-0.146041000
C	-1.683276000	-2.907661000	-0.182747000
C	0.270219000	3.566617000	-0.074579000
C	-5.900610000	4.132856000	0.221352000
H	-6.798100000	4.709619000	0.416354000
C	-3.623966000	3.704476000	-0.048764000
C	-4.168997000	-2.539302000	-0.274937000
C	-8.267279000	-0.277255000	-0.426410000
H	-9.261304000	0.099999000	-0.624786000
C	-0.128243000	4.907772000	-0.128080000
H	0.586254000	5.723546000	-0.145268000
C	-4.624503000	4.662928000	0.173145000
H	-4.398864000	5.710592000	0.333832000
C	-10.480342000	3.187021000	1.210858000
H	-11.096270000	3.107408000	2.104731000
C	-2.223406000	3.882864000	-0.077433000
C	-6.811547000	-4.425606000	1.125202000

C	-8.885826000	3.408694000	-1.074964000
C	-3.411301000	-1.431189000	-0.707134000
H	-3.862738000	-0.546398000	-1.139344000
C	-6.062019000	-4.965734000	-1.148085000
C	-8.798694000	1.745099000	2.380458000
H	-7.764275000	1.975976000	2.656805000
H	-9.428448000	1.955337000	3.246832000
H	-8.843172000	0.663527000	2.204500000
C	-7.344273000	-5.708581000	1.224206000
H	-7.826224000	-6.004556000	2.154059000
C	-7.272462000	-6.625003000	0.172287000
C	-10.131258000	4.030924000	-1.003454000
H	-10.483445000	4.594856000	-1.865217000
C	-6.630133000	-6.229102000	-1.003302000
H	-6.578048000	-6.923988000	-1.839428000
C	-6.905133000	-3.488939000	2.295228000
H	-7.656521000	-2.705795000	2.138531000
H	-7.187458000	-4.026504000	3.202307000
H	-5.955498000	-2.979677000	2.492182000
C	-10.942384000	3.941485000	0.129751000
C	-2.048357000	-1.632155000	-0.642073000
H	-1.311992000	-0.907971000	-0.979551000
C	-8.068323000	3.519770000	-2.329079000
H	-7.631442000	2.559349000	-2.622044000
H	-8.678239000	3.876481000	-3.161076000
H	-7.233394000	4.221714000	-2.219001000
C	-5.419024000	-4.583153000	-2.449031000
H	-4.325116000	-4.560947000	-2.380544000
H	-5.676215000	-5.296717000	-3.233944000

H	-5.732840000	-3.589716000	-2.785683000
C	-12.258640000	4.649291000	0.193074000
H	-12.145246000	5.647052000	0.631304000
H	-12.694692000	4.784644000	-0.799208000
H	-12.978979000	4.111071000	0.813599000
C	-7.890566000	-7.981656000	0.290954000
H	-8.932602000	-7.966126000	-0.047299000
H	-7.369143000	-8.720502000	-0.321920000
H	-7.897569000	-8.335358000	1.324460000

15A.2H^{+}:

Se	0.000558000	4.193413000	0.304719000
S	3.810454000	1.823468000	-0.228211000
S	-3.809987000	1.823940000	-0.226323000
N	6.206704000	-0.006044000	-0.065313000
C	7.590666000	3.515101000	-0.037613000
C	6.543675000	2.461997000	-0.071665000
C	6.975787000	1.131400000	-0.225168000
C	8.461648000	3.595221000	1.070551000
C	5.201017000	2.859779000	0.066391000
C	9.580413000	5.500842000	0.032792000
C	1.301195000	2.848942000	0.168716000
C	4.755225000	4.150437000	0.419080000
H	5.460279000	4.942154000	0.647565000
C	3.380950000	4.281879000	0.472545000
H	2.869413000	5.197957000	0.750323000
C	-2.685510000	3.100176000	0.177877000
C	7.708182000	4.426128000	-1.108944000
C	9.432733000	4.594216000	1.085265000

H	10.090370000	4.671816000	1.949111000
C	2.686328000	3.099590000	0.177298000
C	-1.300438000	2.849252000	0.168994000
C	6.985879000	-1.135820000	-0.235505000
C	8.707849000	5.395764000	-1.052618000
H	8.811773000	6.087965000	-1.886182000
C	0.691092000	1.593724000	0.052625000
H	1.279912000	0.681155000	0.008476000
C	6.548915000	-2.463546000	-0.084589000
C	8.280890000	0.689894000	-0.577722000
H	9.094289000	1.357195000	-0.827622000
C	-0.690648000	1.593886000	0.052792000
H	-1.279682000	0.681447000	0.008762000
C	-5.200273000	2.860587000	0.068236000
C	7.582934000	-3.514716000	0.046118000
C	8.287935000	-0.683860000	-0.579257000
H	9.107545000	-1.342870000	-0.830889000
C	8.337481000	2.660492000	2.239309000
H	8.733437000	1.662519000	2.014988000
H	7.295343000	2.522813000	2.546800000
H	8.891426000	3.038288000	3.100717000
C	7.695635000	-4.517287000	-0.946564000
C	-3.379864000	4.282800000	0.472473000
H	-2.868076000	5.199066000	0.749165000
C	10.657206000	6.538837000	0.057345000
H	11.585656000	6.148913000	-0.374343000
H	10.890411000	6.856976000	1.076134000
H	10.382876000	7.422290000	-0.523615000
C	6.808181000	4.351397000	-2.308015000

H	5.812636000	4.762582000	-2.103343000
H	6.656310000	3.320837000	-2.645224000
H	7.223628000	4.918391000	-3.143138000
C	8.460254000	-3.505058000	1.154050000
C	9.427972000	-4.503606000	1.248734000
H	10.090867000	-4.509394000	2.111868000
C	-4.754170000	4.151465000	0.419715000
H	-5.459119000	4.943394000	0.647685000
C	9.567886000	-5.496891000	0.278070000
C	8.693684000	-5.478103000	-0.812985000
H	8.800450000	-6.231600000	-1.591214000
C	6.808364000	-4.541021000	-2.158051000
H	6.609760000	-3.537488000	-2.546850000
H	5.832680000	-4.994447000	-1.947878000
H	7.264813000	-5.125335000	-2.959285000
C	8.353624000	-2.482300000	2.249480000
H	7.314286000	-2.226343000	2.478333000
H	8.860874000	-1.544952000	1.991642000
H	8.815649000	-2.850753000	3.167576000
C	10.610110000	-6.561617000	0.404221000
H	10.160188000	-7.520855000	0.682183000
H	11.352533000	-6.316520000	1.166186000
H	11.133248000	-6.725655000	-0.541779000
Se	-0.000061000	-4.045146000	0.333714000
S	-3.843836000	-1.866475000	-0.596210000
S	3.844522000	-1.867102000	-0.594365000
N	-6.206141000	-0.005412000	-0.065608000
C	-7.582644000	-3.514099000	0.041781000
C	-6.548649000	-2.462804000	-0.088021000

C	-6.985464000	-1.134869000	-0.237274000
C	-7.693046000	-4.518205000	-0.948536000
C	-5.192292000	-2.850244000	-0.035110000
C	-9.559842000	-5.503182000	0.280105000
C	-1.301395000	-2.902004000	-0.383381000
C	-4.710911000	-4.092112000	0.417552000
H	-5.386727000	-4.848843000	0.800450000
C	-3.336552000	-4.227730000	0.345374000
H	-2.797424000	-5.116401000	0.658484000
C	2.684328000	-3.109823000	-0.188562000
C	-8.456783000	-3.507624000	1.153186000
C	-8.686642000	-5.484088000	-0.810584000
H	-8.787877000	-6.243330000	-1.583888000
C	-2.684027000	-3.109429000	-0.190000000
C	1.301831000	-2.902216000	-0.382693000
C	-6.975141000	1.132345000	-0.223757000
C	-9.418942000	-4.510063000	1.252111000
H	-10.074505000	-4.521785000	2.120809000
C	-0.693275000	-1.847009000	-1.071137000
H	-1.281612000	-1.101534000	-1.599962000
C	-6.542959000	2.462790000	-0.068802000
C	-8.287497000	-0.682275000	-0.580255000
H	-9.107190000	-1.340842000	-0.832695000
C	0.694243000	-1.847135000	-1.070791000
H	1.282978000	-1.101768000	-1.599327000
C	5.192533000	-2.850860000	-0.032233000
C	-7.590373000	3.515518000	-0.033407000
C	-8.280358000	0.691473000	-0.576780000
H	-9.093771000	1.359193000	-0.825642000

C	-6.804867000	-4.544594000	-2.159267000
H	-6.600359000	-3.541518000	-2.545982000
H	-5.832013000	-5.004119000	-1.949112000
H	-7.263895000	-5.124871000	-2.961990000
C	-8.463826000	3.591825000	1.072649000
C	3.336433000	-4.228143000	0.347291000
H	2.797021000	-5.116735000	0.660135000
C	-10.628292000	-6.543287000	0.390164000
H	-11.529474000	-6.234542000	-0.151208000
H	-10.312539000	-7.496468000	-0.040540000
H	-10.922484000	-6.715340000	1.427817000
C	-8.345550000	-2.487033000	2.250193000
H	-7.304819000	-2.239498000	2.482365000
H	-8.844379000	-1.545315000	1.991935000
H	-8.813105000	-2.852923000	3.166476000
C	-7.713501000	4.422293000	-1.108062000
C	-8.720615000	5.384112000	-1.056335000
H	-8.833665000	6.067700000	-1.895791000
C	4.710754000	-4.092610000	0.420301000
H	5.386315000	-4.849327000	0.803633000
C	-9.592705000	5.488754000	0.029925000
C	-9.442893000	4.583403000	1.082735000
H	-10.107971000	4.653276000	1.941536000
C	-8.340790000	2.655708000	2.240406000
H	-8.740080000	1.659095000	2.015890000
H	-7.298618000	2.514864000	2.546159000
H	-8.892332000	3.034420000	3.102965000
C	-6.816473000	4.344501000	-2.309146000
H	-5.818298000	4.749846000	-2.105768000

H	-6.670716000	3.313607000	-2.648267000
H	-7.230530000	4.914958000	-3.142577000
C	-10.641631000	6.554346000	0.074270000
H	-10.243288000	7.477577000	0.509534000
H	-11.005607000	6.806337000	-0.924525000
H	-11.496576000	6.257054000	0.685689000
H	-5.330728000	-0.019195000	0.440564000
H	5.331261000	-0.018982000	0.440833000

15B.2H$^+$:

S	-4.387283000	2.024777000	0.229014000
S	-3.060028000	-3.884458000	-0.204514000
Se	-1.134493000	2.207994000	-0.020511000
N	-6.084370000	-0.355997000	-0.033064000
H	-5.261914000	-0.110659000	-0.565154000
C	-6.436547000	-1.672437000	0.191953000
C	-6.100252000	-4.126227000	0.106685000
C	-7.187181000	0.467515000	0.091729000
C	-7.179789000	1.873028000	-0.000062000
C	-8.492129000	2.566131000	-0.021348000
C	-7.825694000	-1.675104000	0.488689000
H	-8.383344000	-2.559807000	0.764564000
C	-5.539939000	-2.757456000	0.176083000
C	-6.008717000	2.653453000	-0.037886000
C	-9.326702000	2.441054000	-1.153235000
C	-1.485562000	4.884730000	0.129542000
H	-1.949898000	5.863439000	0.185441000
C	-1.650949000	-2.895160000	0.080569000
C	0.331903000	3.379936000	0.029343000

C	-5.926946000	4.049664000	-0.219644000
H	-6.817481000	4.640545000	-0.405081000
C	-3.653668000	3.591356000	0.039950000
C	-4.142680000	-2.565397000	0.213412000
C	-8.279200000	-0.376652000	0.433930000
H	-9.272761000	-0.013334000	0.659274000
C	-0.108908000	4.703629000	0.125451000
H	0.592100000	5.532044000	0.156683000
C	-4.643762000	4.565275000	-0.163068000
H	-4.407794000	5.613064000	-0.307827000
C	-10.557273000	3.093826000	-1.150492000
H	-11.195380000	3.010514000	-2.028305000
C	-2.249253000	3.714541000	0.065771000
C	-6.757006000	-4.554464000	-1.068210000
C	-8.906249000	3.325386000	1.094208000
C	-3.393276000	-1.424082000	0.568051000
H	-3.848166000	-0.528977000	0.972700000
C	-5.980091000	-4.992149000	1.217416000
C	-8.904924000	1.650338000	-2.357821000
H	-7.875286000	1.874334000	-2.656412000
H	-9.551740000	1.864419000	-3.210587000
H	-8.953171000	0.569287000	-2.180563000
C	-7.261659000	-5.851761000	-1.115280000
H	-7.744578000	-6.192389000	-2.029230000
C	-7.160859000	-6.726681000	-0.030924000
C	-10.154153000	3.944977000	1.051903000
H	-10.486321000	4.509818000	1.920994000
C	-6.519724000	-6.272811000	1.124233000
H	-6.445573000	-6.934767000	1.985060000

C	-6.881607000	-3.661453000	-2.268767000
H	-7.632540000	-2.875970000	-2.122396000
H	-7.181862000	-4.232070000	-3.149478000
H	-5.938918000	-3.155981000	-2.505348000
C	-10.993153000	3.851179000	-0.060551000
C	-2.028837000	-1.601716000	0.475101000
H	-1.302740000	-0.834551000	0.731974000
C	-8.061616000	3.439958000	2.330165000
H	-7.594527000	2.487217000	2.600273000
H	-8.660672000	3.770961000	3.180556000
H	-7.247366000	4.164267000	2.210926000
C	-5.339039000	-4.543067000	2.497834000
H	-4.249391000	-4.460387000	2.410920000
H	-5.544443000	-5.249420000	3.304185000
H	-5.703683000	-3.560339000	2.814875000
C	-12.311549000	4.556889000	-0.092721000
H	-12.207733000	5.559258000	-0.522771000
H	-12.729341000	4.681852000	0.908783000
H	-13.042279000	4.023514000	-0.705132000
C	-7.748341000	-8.100618000	-0.094510000
H	-8.788324000	-8.096369000	0.250289000
H	-7.206056000	-8.803935000	0.541647000
H	-7.753595000	-8.492822000	-1.114037000
S	4.387272000	-2.024771000	-0.229017000
S	3.060032000	3.884448000	0.204596000
Se	1.134499000	-2.208018000	0.020577000
N	6.084377000	0.356015000	0.033097000
H	5.261914000	0.110672000	0.565173000
C	6.436566000	1.672456000	-0.191892000

C	6.100259000	4.126252000	-0.106649000
C	7.187181000	-0.467503000	-0.091717000
C	7.179782000	-1.873017000	0.000037000
C	8.492118000	-2.566126000	0.021293000
C	7.825710000	1.675119000	-0.488644000
H	8.383364000	2.559822000	-0.764513000
C	5.539957000	2.757473000	-0.176009000
C	6.008714000	-2.653447000	0.037841000
C	9.326762000	-2.440967000	1.153116000
C	1.485574000	-4.884744000	-0.129625000
H	1.949912000	-5.863448000	-0.185586000
C	1.650965000	2.895128000	-0.080475000
C	-0.331892000	-3.379963000	-0.029305000
C	5.926959000	-4.049669000	0.219510000
H	6.817503000	-4.640554000	0.404891000
C	3.653675000	-3.591364000	-0.040013000
C	4.142700000	2.565391000	-0.213299000
C	8.279209000	0.376664000	-0.433895000
H	9.272766000	0.013342000	-0.659245000
C	0.108920000	-4.703649000	-0.125493000
H	-0.592085000	-5.532064000	-0.156752000
C	4.643779000	-4.565287000	0.162928000
H	4.407822000	-5.613086000	0.307630000
C	10.557330000	-3.093746000	1.150344000
H	11.195498000	-3.010362000	2.028105000
C	2.249261000	-3.714555000	-0.065809000
C	6.757036000	4.554519000	1.068226000
C	8.906154000	-3.325476000	-1.094226000
C	3.393314000	1.424037000	-0.567852000

H	3.848222000	0.528909000	-0.972435000
C	5.980064000	4.992153000	-1.217393000
C	8.905070000	-1.650131000	2.357652000
H	7.875460000	-1.874112000	2.656350000
H	9.551958000	-1.864110000	3.210389000
H	8.953284000	-0.569097000	2.180273000
C	7.261676000	5.851821000	1.115260000
H	7.744611000	6.192472000	2.029193000
C	7.160844000	6.726721000	0.030888000
C	10.154054000	-3.945077000	-1.051953000
H	10.486155000	-4.510000000	-1.921017000
C	6.519688000	6.272823000	-1.124245000
H	6.445512000	6.934762000	-1.985084000
C	6.881668000	3.661535000	2.268800000
H	7.632606000	2.876057000	2.122434000
H	7.181932000	4.232176000	3.149493000
H	5.938987000	3.156059000	2.505409000
C	10.993130000	-3.851193000	0.060436000
C	2.028871000	1.601661000	-0.474919000
H	1.302784000	0.834471000	-0.731750000
C	8.061415000	-3.440166000	-2.330098000
H	7.594290000	-2.487454000	-2.600250000
H	8.660397000	-3.771240000	-3.180513000
H	7.247183000	-4.164473000	-2.210719000
C	5.338990000	4.543044000	-2.497790000
H	4.249341000	4.460386000	-2.410861000
H	5.544397000	5.249370000	-3.304165000
H	5.703614000	3.560300000	-2.814808000
C	12.311522000	-4.556912000	0.092576000

H	12.207726000	-5.559245000	0.522718000
H	12.729245000	-4.681963000	-0.908945000
H	13.042298000	-4.023493000	0.704893000
C	7.748314000	8.100664000	0.094439000
